# Correlating genomic copy number alterations with clinicopathologic findings in 75 cases of hepatocellular carcinoma

**DOI:** 10.1186/s12920-021-00998-9

**Published:** 2021-06-08

**Authors:** Gang Peng, Hongyan Chai, Weizhen Ji, Yufei Lu, Shengming Wu, Hongyu Zhao, Peining Li, Qiping Hu

**Affiliations:** 1 Department of Biostatistics, School of Public Health, Yale University, New Haven, CT USA; 2 Department of Genetics, School of Medicine, Yale University, New Haven, CT USA; 3 Department of Cell Biology and Genetics, School of Pre-Clinical Medicine, Guangxi Medical University, Nanning, Guangxi People’s Republic of China; 4 Department of Pathology, Affiliated Tumor Hospital of Guangxi Medical University, Nanning, Guangxi People’s Republic of China

**Keywords:** Hepatocellular carcinoma (HCC), Array comparative genomic hybridization (aCGH), Copy number aberrations (CNAs), Edmondson-Steiner (E-S) grading, Barcelona-Clinic Liver Cancer (BCLC) stages

## Abstract

**Background:**

Oligonucleotide array comparative genomic hybridization (aCGH) analysis has been used for detecting somatic copy number alterations (CNAs) in various types of tumors. This study aimed to assess the clinical utility of aCGH for cases of hepatocellular carcinoma (HCC) and to evaluate the correlation between CNAs and clinicopathologic findings.

**Methods:**

aCGH was performed on 75 HCC cases with paired DNA samples from tumor and adjacent nontumor tissues. Survival outcomes from these cases were analyzed based on Barcelona-Clinic Liver Cancer Stage (BCLC), Edmondson-Steiner grade (E-S), and recurrence status. Correlation of CNAs with clinicopathologic findings was analyzed by Wilcoxon rank test and clustering vs. K means.

**Results:**

The survival outcomes indicated that BCLC stages and recurrence status could be predictors and E-S grades could be a modifier for HCC. The most common CNAs involved gains of 1q and 8q and a loss of 16q (50%), losses of 4q and 17p and a gain of 5p (40%), and losses of 8p and 13q (30%). Analyses of genomic profiles and clusters identified that losses of 4q13.2q35.2 and 10q22.3q26.13 seen in cases of stage A, grade III and nonrecurrence were likely correlated with good survival, while loss of 1p36.31p22.1 and gains of 2q11.2q21.2 and 20p13p11.1 seen in cases of stage C, grade III and recurrence were possibly correlated with worst prognosis.

**Conclusions:**

These results indicated that aCGH analysis could be used to detect recurrent CNAs and involved key genes and pathways in patients with HCC. Further analysis on a large case series to validate the correlation of CNAs with clinicopathologic findings of HCC could provide information to interpret CNAs and predict prognosis.

**Supplementary information:**

The online version contains supplementary material available at 10.1186/s12920-021-00998-9.

## Background

Hepatocellular carcinoma (HCC) is the seventh most common cancer with estimated 0.84 million new cases per year and the fifth leading cause of cancer-related mortality with estimated 0.78 million deaths per year worldwide [1]. Through understanding of genetic defects affecting tumorigenesis and progression of HCC could be useful for accurate diagnosis, precisive treatment, and prognosis prediction. Earlier studies for genome-wide copy number alterations (CNAs) in HCC had been performed using BAC clone array-based comparative genomic hybridization (aCGH) or single-nucleotide polymorphism arrays [2–6]. In the past decade, integrated genomic analyses using aCGH, gene expression array, whole exome and genome sequencing have been applied to identify key genes and core pathways for the initiation and progression of HCC [7–11]. Recurrent CNAs correlating with stages and prognosis of HCC [12–16] and associating with infections by Hepatitis B or C virus [17, 18] have been reported. Accumulated genetic and genomic data could be translated to clinical service for better genetic diagnosis and prognosis prediction. However, the interpretation of CNAs for HCC in a diagnostic setting could still be a challenge due to the lack of consensus on the correlation of CNAs with clinicopathologic findings.

In the present study, we performed aCGH analysis on 75 pairs of tumor and adjacent nontumor tissues from HCC patients. Genomic profiles and clusters of CNAs for all cases and cases with different clinicopathologic findings were compared to evaluate correlations of CNAs with tumor stages, grades and recurrence. These results further confirmed the diagnostic value of aCGH and provided preliminary data for a large-scale analysis of genomic and clinicopathologic correlation for HCC.

## Methods

### Patients and sample collection

We selected 79 HCC patients with pathologic diagnosis, surgical treatment, and clinic follow-up results in the Affiliated Tumor Hospital of Guangxi Medical University from 2014 to 2016. Among them, 72 were male and seven were female; 67 were less than 60 years old and 12 were more than 60 years old, 35 were grade II and 44 were grade III by Edmondson-Steiner (E-S) grading; 32 were stage A, 12 were stage B, and 35 were stage C by Barcelona-Clinic Liver Cancer Stage (BCLC) classification; 29 with recurrence and 50 without recurrence. As of June 30, 2018, 31 of the 79 patients were alive, 38 died and 10 lost to follow-up. Formalin-fixed paraffin embedded (FFPE) tumor tissue blocks from these patients were collected from the tumor biobank of Guangxi Medical University. This research project was approved by the Medical Ethics Committee of Guangxi Medical University and research applications of residual specimens followed university policies and laboratory standards [19].

### aCGH analysis

For each case, FFPE blocks of tumor tissue and adjacent nontumor tissue were dissected as a pair of test and control. DNA was extracted from dissected tissues using Qiagen tissue kit following the manufacturer’s instructions (Qiagen, Chatsworth, CA). Oligonucleotide aCGH assay was performed as previously described [20]. Briefly, differentially labeled test or control DNA and gender-matched normal reference DNA were co-hybridized to a SurePrint G3 Human CGH 8 × 60 K Microarray slide (Agilent Technologies). Post-hybridization image capture, signal feature extraction and copy number analysis were performed using Agilent’s Cytogenomics 2.5 [21, 22]. Benign copy number variants recognized in the Database of Genomic Variants (http://dgv.tcag.ca/dgv/app/home) were excluded. CNAs detected from the tumor tissue and undetected in the paired adjacent nontumor tissue were considered pathogenic. The nucleotide positions for CNAs were designated according to the NCBI36/hg18 assembly in the Human Genome browser (http://genome.ucsc.edu/). The smallest overlap regions (SORs) were defined as the shared overlapped regions of CNAs. A relative frequency for each SOR was calculated by the number of cases with overlapped CNAs divided by the number of all cases. Genomic profiles for SORs of CNAs in all cases and in cases by different clinicopathologic classifications were generated. Detailed description of SOR is provided in supplemental method. The percentage of CNAs in each case was calculated by the total size of CNAs divided by the size of human genome.

### Statistical analysis

We compared the survive between different clinicopathologic classifications of E-S grading, BCLC stages, and recurrence status using Kaplan–Meier method with *p*-value calculated from log-rank test [23]. Considering the interaction among these three clinical variables, we stratified the survival analysis on recurrence status and E-S grade at BCLC stages A and C. There were 32 patients at BCLC stage A and 35 patients at BCLC stage C. The hazard ratio between E-S grades 3 and 2 is 0.17 for these patients at BCLC stage A when we used Cox Proportional-Harzards Model; it was estimated that a power of 95% at the significant level of 0.05 can be achieved when the sample size is 32 or more in our analysis [24]. We didn’t perform stratified survive analysis on cases in BCLC stage B because of the limited sample size of only 12 cases. The correlation between the clinical variables were tested by Fisher’s exact test.

We compared the genomic profiles of CNAs by relative frequencies of SORs between cases of different E-S grades, BCLC stages and recurrence status. P-value was calculated from Wilcoxon rank test when comparing the changes of CNAs in selected regions between case classifications. We also used receiver operating characteristic (ROC) curve to evaluate the association between the percentage of genomic CNAs for the three clinicopathologic classifications. In this analysis, the percentage of the CNAs in each case was used to predict the three classifications of the patients. Furthermore, K-means was used to cluster the cases according to SORs of CNAs for all cases and the cases in different BCLC stages. The size of SORs was used as the weight in clustering considering that CNAs with larger SOR may have more influence on the tumors. There were both male and female patients in our analysis. We did not include chromosome X and Y in clustering to avoid the bias of clustering cases with same gender together. Statistical analyses were performed using statistical computing software R 3.6.1. [25].

## Results

### Survival outcomes from different clinicopathologic classifications

**Fig. 1:**
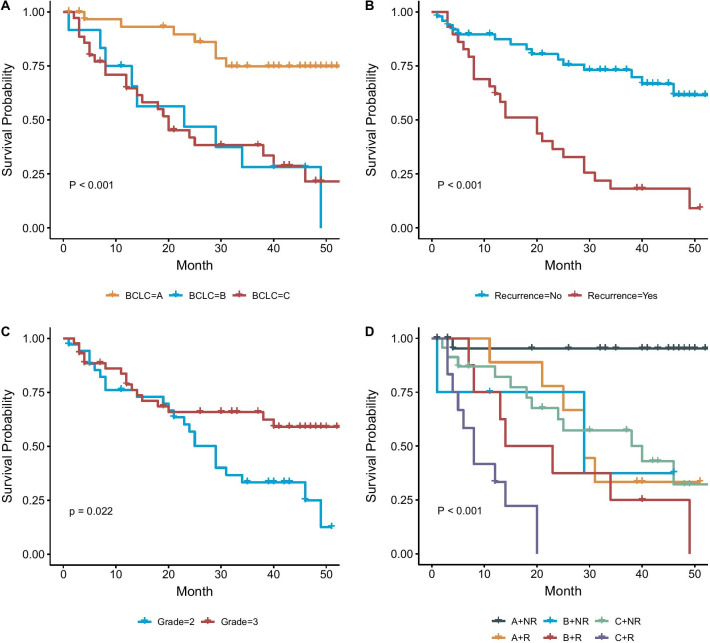
Survival outcomes with different clinicopathologic classifications. **a** Survival outcome for the cases with different BCLC stages. **b** Survival outcome difference between the cases with and without recurrence. **c** Survival outcome difference between the cases in E-S grade II and grade III. **d** Survival outcome for cases with different BCLC stages and recurrence status. G2: E-S Grade II; G3: E-S Grade III; NR: No Recurrence; R: Recurrence; A: BCLC stage A; B: BCLC stage B; C: BCLC stage C

The clinicopathologic classifications of the 79 HCC cases were summarized in Table 1. Survival outcomes from these cases were compared based on their classifications of BCLC stages, recurrence status, and E-S grades. The Kaplan–Meier analysis showed that cases in BCLC stage A had longer survive than cases in BCLC stage B and C ////(*P* < 0.001) (Fig. 1a). Cases without recurrence had better prognosis than cases with recurrence (*P* < 0.001) (Fig. 1b). Cases in E-S grade III seemed to have longer survival than cases in grade II (*P* = 0.022) (Fig. 1c). When we took the survival analysis on different combinations of stages and recurrence, cases without recurrence in stage A had the best prognosis, while cases with recurrence in stage C had the worst prognosis (Fig. 1d). Considering the interactions between the three clinical classifications, we further stratified all cases into different BCLC stages A and C and evaluated the influence on survival by recurrence status and E-S grades. Cases without recurrence had better prognosis than those with recurrence in both stages A and C (*P* < 0.001) (Additional file 1: Fig 1A/B). Cases in E-S grade III had better prognosis than grade II only in stage A (*P* = 0.018) but not in stage C (Additional file 1: Fig. 1C/D). It was also noted that cases in E-S grade III was significantly associated with non-recurrence in BCLC stage A (*P* < 0.001) but had no significant association between recurrence and non-recurrence in BCLC stage C (*P* = 1.00) (Table 1). These results indicated that the BCLC stages and recurrence status could be an independent predictor for prognosis and E-S grades might be a modifying predictor affected by stage and recurrence. However, the overall sample size was limited and thus this preliminary observation would need further validation from a large case series.

**Table 1:** Sample size and case number in different clinicopathologic classifications

		Barcelona-clinic liver cancer stage					
		A		B		C	
		No recurrence	Recurrence	No recurrence	Recurrence	No recurrence	Recurrence
Grade	2	2	8	4	6	10	5
	3	21	1	0	2	13	7

### Genomic profile of CNAs and correlation of CNAs with clinicopathologic classifications

**Fig. 2:**
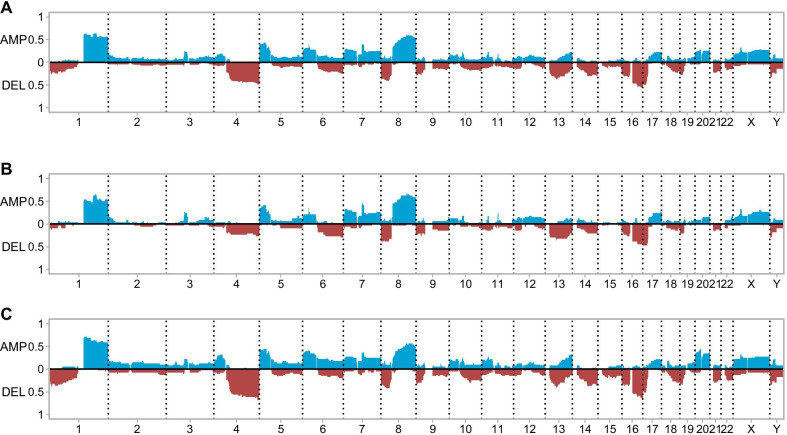
The genomic profiles of CNAs all cases and cases in different E-S grades. **a** The distribution of CNAs in all 75 cases. **b** The distribution of CNAs in 34 cases of E-S grade II. **c** The distribution of CNAs in 41 cases of E-S grade III. (duplications/gains in blue and deletions/losses in red)

Of the 79 cases subjected for aCGH analysis, 95% (75/79) were successful to have results from both tumor and adjacent nontumor DNA pairs. All DNAs from adjacent nontumor tissues showed normal results and CNAs detected in tumor DNAs were summarized in Additional file 2: Table 1. The genomic profile of CNAs in all 75 cases was shown in Fig. 2a. Of the CNAs from these 75 cases, more than 50% cases had gains of 1q and 8q and a loss of 16q, more than 40% cases had losses of 4q and 17p, and a gain of 5p, and more than 30% cases had losses of 8p and 13q.

The genomic profiles of CNAs for cases in E-S grades II and III were shown in Fig. 2b/C, respectively. There were significantly more copy number losses of 1p36.31p22.1 (chr1:6,334,098–93,461,630, 87.1 Mb) (*P* = 0.004), 4q13.2q35.2 (chr4:68,288,998–190,706,331, 122.4 Mb) (*P* = 0.001) and 10q22.3q26.13 (chr10:78,314,145–124,106,670, 45.8 Mb) (*P* = 0.010), and more copy number gain of 20p13p11.1 (chr20:1,211,324–25,680,554, 24.5 Mb) (*P* = 0.001) for cases in grade III than in grade II. The genomic profiles of CNAs for cases in different BCLC stages and recurrence status were shown in Additional file 1: Figs 2 and 3, respectively. There was no significant difference of CNAs between cases in different BCLC stages A and C and with or without recurrence.

We further used ROC curve to check the association of the percentage of genomic CNAs of each case with clinicopathologic classifications. We found that the area under the curve (AUC) were 0.68 (95% CI = [0.56, 0.81]), 0.57 (95% CI = [0.42, 0.72]), 0.53 (95% CI = [0.39, 0.66]), and 0.61 (95% CI = [0.48, 0.74]) for E-S grades, recurrence status, BCLC stage A versus stage B + C, and stage A + B versus stage C, respectively (Additional file 1: Fig. 4). These results indicated that there were more CNAs in E-S grade III than grade II (*P* = 0.003, one tail Wilcoxon rank test) but the percentage of genomic CNAs had no significant association with E-S grades, BCLC stages and recurrence.

During the analysis of survival outcome and clinicopathologic classifications, we found the impact of E-S grades and recurrence on BCLC stages (Additional file 1: Fig. 1). We further stratified cases into BLCL stage A and C to evaluate any difference in CNAs. The significant difference between E-S grades II and III in BCLC stage A was more losses of 4q13.2q35.2 (*P* = 0.003 in BCLC A; *P* = 0.80 in BLCL C) and 10q22.3q26.13 (*P* = 0.033 in BCLC A; *P* = 0.55 in BCLC C) in grade III; the significant difference between E-S grades II and III in BCLC stage C was more gains of 2q11.2q21.2 (chr2:98,228,328–134,727,485, 36.5 Mb) (*P* = 1 in BCLC A, *P* = 0.008 in BCLC C) and 20p13p11.1 (*P* = 0.59 in BCLC A; *P* = 0.005 in BCLC C) in grade III (Additional file 1: Fig. 5).

### Cluster analysis of CNAs for clinical classifications

**Fig. 3:**
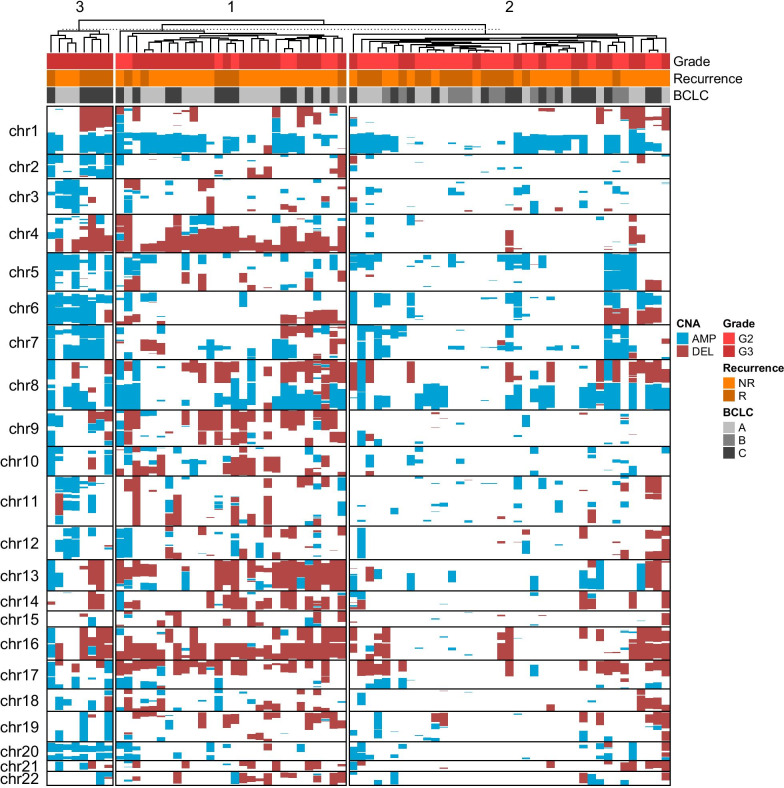
Clusters of all 75 cases according to genomic CNAs. Color coding for E-S grades, recurrences, and BCLC stages in clusters and for deletion and amplification of CNAs is shown

We used k-means method to cluster patterns of CNAs into three clusters for all cases and cases in BCLC stages A and C. The CNAs from all 75 cases were divided into three clusters as shown in Fig. 3. The 28 cases in cluster 1 had more CNA losses, the 39 cases in cluster 2 had less CNAs, and the eight cases in cluster 3 had more CNA gains. There were more CNAs in clusters 1 and 3 than cluster 2. The E-S grades, BCLC stages and recurrence status were mixed in clusters 1 and 2, while the eight cases in cluster 3 were all E-S grade III and further divided into BCLC stage A without recurrence and BCLC stage C with recurrence. Further clustering analysis was performed on cases in BCLC stage A and C. For the 32 cases in BCLC stage A, 14 cases were in cluster 1 with more CNA losses and 12 of them were classified as E-S grade III and nonrecurrence, 15 cases were in cluster 2 with less CNAs and mixed E-S grades and recurrence statues, and three cases in cluster 3 with more CNA gains were all in E-S grade III and nonrecurrence (Additional file 1: Fig. 6). For the 31 cases in BCLC stage C, 12 cases were in cluster 1 with more CNA losses, 14 cases were in cluster 2 with less CNAs, and five cases were in cluster 3 with more CNA gains. It was noted that cases in clusters 1 and 2 were mixed in E-S grades and recurrence status, while cases in cluster 3 was associated with E-S grade III and recurrence (Additional file 1: Fig. 7).

Survival differences between the three clusters in all three data sets were shown in Additional file 1: Fig. 8. For all 75 cases, cases in cluster 3 had worse survival than cases in clusters 1 and 2 (*P* = 0.021, Additional file 1: Fig. 8A). For cases in BCLC stage A, cases in cluster 1 showed better survival than cases in clusters 2 and 3 (*P* = 0.047); for cases in BCLC stage C, cases in clusters 3 showed the worst prognosis than cases in clusters 1 and 2 (Additional file 1: Fig. 8B/C). Further analysis focused on a comparison between cases with the best prognosis by BCLC stage A and nonrecurrence and cases with the worse prognosis by BCLC stage C and recurrence (Fig. 1d). Of the 21 cases classified as BCLC stage A, E-S grade III and nonrecurrence in Table 1, there were 12, six and three cases in clusters 1, 2 and 3, respectively. The losses of 4q13.2q35.2 and 10q22.3q26.13 associated with E-S grade III were seen in cases of cluster 1, which suggested these CNAs might play a protective or modify role for survive outcome. Other gains of chromosomes 3, 6, 11p, 12q and 20 in cluster 3 probably related to poor prognosis (Additional file 1: Fig. 6). Of the seven cases classified as BCLC stage C, E-S grade III and recurrence in Table 1, there were one case each in clusters 1 and 2 and five cases in cluster 3. The loss of 1p36.31p22.1 and gains of 2q11.2q21.2 and 20p13p11.1 associated with E-S grade III and additional gains of chromosomes 6, 7 and 20q were seen in cluster 3 and thus likely correlated with worst survival (Additional file 1: Fig. 7). Combing clustering and survival results noted that specific CNAs could be indicators for clinicopathologic classifications and survival prediction.

## Discussion

We performed first survival analysis based on BCLC stages, E-S grades and recurrent status and then tried to correlate genomic profile of CNAs with clinicopathologic classifications. The BCLC staging classifies HCC based on liver functional status, physical status and cancer-related symptoms and linked the four stages with treatment algorithm [26, 27]. E-S grading is determined by tumor histologic and cytologic findings [28, 29]. The general genomic profile of CNAs from these cases showed a consistent or similar pattern of CNAs from previous studies [3–18]. The most commonly seen CNAs in more than 30% of our cases, including gains of 1q, 5p and 8q and losses of 4q, 8p, 13q, 16q and 17p as shown in Fig. 2a, were all recurrent CNAs for HCC. Bioinformatic and gene expression analyses had been used to define candidate genes from these recurrent CNAs. Candidate genes, key genes and pathways from nine studies were listed in Additional file 2: Table. 2. Further gene functional analysis validated the causal or modifying roles of some of these CNAs and associated genes for HCC. Analysis of gene expression levels in CNAs noted that gain or amplification of the *MDM4* gene at 1q32 associated with poor metastasis-free survival [5, 16]. Mechanistically, gains of 1q resulted from hypomethylation of 1q heterochromatin induced jumping translocations were seen in myelodysplastic syndrome acute myeloid leukemia [30]. Gains of 8q involving the *MYC* gene overexpression was observed in viral and alcohol-related HCCs [5]. Significantly upregulated genes *ATAD2*, *SQLE*, *PVT1*, *ASAP1* and *NDRG1* from gains of 8q24.13q24.3 were an unfavorable prognostic marker for HCCs [15]. Loss of 8p involving the *DLC1*, *CCDC25*, *ELP3*, *PROSC*, *SORBS3*, *SH2D4A* genes associated with poor outcomes for HCC; in vitro and in vivo analysis indicated that the *PROCS*, *SH2D4A* and *SORBS3* genes have tumor-suppressive activities and the *DLC1* gene is a known tumor suppressor gene [9]. Integrated analysis of somatic mutations and CNAs in HCCs identified 56 key genes and five pathways for HCC [8]. At least 38% (21/56) of these key genes could be mapped to the recurrent CNAs and affected four core pathways of p53/cell cycle control, chromatin remodeling, PI3K/Ras signaling, and oxidative and endoplasmic reticulum stress (Additional file 2: Table 2). Multiple genes from recurrent CNAs affected the p53/cell cycle control, including the *CDKN2C* gene at 1p32.3, the *CDK11A/B* genes at 1p36.33, the *IRF2* gene at 4q35, the *RB1* gene at 13q14.2 and the *TP53* gene at 17p13.1. These recurrent CNAs and affected genes and pathways were an integral part of the comprehensive genetic landscape and genomic characterization of HCC [31, 32].

Correlating genomic CNAs with the HCC stages and grades could be helpful to interpret test results and guide clinical treatment. A dendrogram of the cluster analysis showed first the gain of 1q then gains of 8q and 5p followed by other CNAs; the gains of 1q and 8q were significantly correlated with E-S grades II-IV [18]. Another study compared the recurrent CNAs between E-S grades I/II and III/IV and noted that gain of 8q was statistically more frequently seen in grade III/IV [14]. In general, cases in E-S grade III seemed to have more CNAs than those in grade II. More specifically, significant correlation of losses of 1p36.31p22.1, 4q13.2q35.2 and 10q22.3q26.13 and gains of 2q11.2q21.2 and 20p13p11.1 with E-S grade III were noted. After we stratified the cases according to BCLC stages, two regions significant correlated with BCLC stage A were losses of 4q13.2q35.2 and 10q22.3q26.13 (Additional file 1: Fig. 3). The 4q13.2q35.2 included the *IRF2* gene for p53/cell cycle control and *SMARCAD1* gene for chromatin remodeling, while the 10q22.3q26.13 included the *PTEN* gene for PI3K/Ras signaling (Additional file 2: Table 2). Counterintuitively, our cases showed a better survival and less recurrence for E-S grade III than grade II at BCLC stage A. One hypothesis to explain this observation was that in BCLC stage A usually involved one tumor and the tumor in E-S grade III may be cytologically more distinct from tumor in grade II, which might make the tumor in grade III more likely to be removed completely during the surgery. This could also explain significantly less recurrence in E-S grade III than in grade II in BCLC stage A (*P* < 0.001). In BCLC stage C, the cancer cells have been spread to blood vessels, lymph nodes or other body organs. The recurrence rate after the surgery is similar between E-S grades II and III in this stage (*P* = 0.72). The loss of 1p36.31p22.1 and gains of 2q11.2q21.2 and 20p13p11.1 and additional gains of chromosomes 6, 7 and 20q were correlated with BCLC stage C, E-S grade III and recurrence for the worst survival outcome. The *ARID1A*, *CDKN2C*, *CDK11A* and *CDK11B* genes at 1p could affect the p53/cell cycle control and chromatin remodeling pathways (Additional file 2: Table 2). A study focused on gene expression from gains of 20q identify candidate genes contributing to unfavorable outcomes for HCC, including overexpression of the *DDX27*, *B4GALT5*, *RNF114*, ZFP64 and *PFDN4* associated significantly with vascular invasion, and high *RNF114* expression associated with advanced tumor stage [12]. We didn’t find significant correlation between CNAs and BCLC stage or recurrence status. The correlation of CNAs with cytologic and histologic findings by E-S grades may reflect a link between molecular and cellular levels. Additionally, clustering analysis noted more CNAs in cases with poor survival outcome but ROC analysis did not support the association of percentage of CNAs with clinicopathologic classifications.

This study provided preliminary results correlating genomic CNAs to clinicopathologic findings. However, two major limitations should be mentioned from this study. The first limitation by the sample size possibly introduced bias in case stratification. The second limitation was the technical challenge in tracking the clonal evolution and dissecting tumor heterogeneity [33, 34]. All tumor specimens were collected at the surgical procedures and thus made it difficult to investigate initial event and accumulated aberrations from different tumor stages.

## Conclusion

Clinical application of aCGH could detect genomic profiles of recurrent CNAs and affected key genes and pathways for HCC. Specific CNAs could be correlated with the cytologic and histologic grading and likely related to the prognosis of HCC. HCC has showed clinical heterogeneity at the clinicopathologic level and genomic heterogeneity from accumulated CNAs, somatic mutations, and epigenetic alterations. A biopsy-based integrative diagnostic approach including morphology, immunohistochemistry, transcriptomic data, mutational profiles, CNA and methylome analysis have been proposed for future analysis of HCC [35].

## Supplementary information

**Additional file 1. Supplemental Method**. Define Smallest Overlap Region (SOR) and calculate relative frequency (RF).** Supplemental Figure 1**. Survival difference between the cases with different recurrence and E-S grades in BCLC stage A and C.** Supplemental Figure 2**. The genomic profiles of CNAs in cases with different BCLC stages.** Supplemental Figure 3**. The genomic profiles of CNAs in cases with or without recurrence.** Supplemental Figure 4**. ROC curves for the prediction performance of percentage of genome change to E-S grades, recurrence, and BCLC stages.** Supplemental Figure 5**. The genomic profile of CNAs in different BCLC stages and E-S grades.** Supplemental Figure 6**. Clusters of the cases by CNAs in BCLC stage A.** Supplemental Figure 7**. Clusters of the cases by CNAs in BCLC stage C.** Supplemental Figure 8**. Survival difference between different clusters in all cases and cases in BCLC stages A and C.

**Additional file 2. Supplemental Table 1**. CNAs detected from 75 cases of HCC.** Supplemental Table 2**. List of candidate and key genes in recurrent CNAs for HCC.

## Data Availability

The datasets used and/or analyzed during the current study are available in Additional file 2: Table 1. The source code and data for smallest overlap regions estimation are available in the github repository [https://github.com/peng-gang/SOR].

## Abbreviations

aCGH: Array comparative genomic hybridization.
BCLC: Barcelona-clinic liver cancer stage
CNAs: Copy number alterations.
FFPE: Formalin-fixed paraffin embedded
HCC: Hepatocellular carcinoma.
ROC: Receiver operating characteristic

## References

[1] Bray F, Ferlay J, Soerjomataram I, Siegel RL, Torre LA, Jemal A (2018). Global cancer statistics 2018: GLOBOCAN estimates of incidence and mortality worldwide for 36 cancers in 185 countries. CA Cancer J Clin.

[2] Patil MA, Gütgemann I, Zhang J, Ho C, Cheung ST, Ginzinger D (2005). Array-based comparative genomic hybridization reveals recurrent chromosomal aberrations and Jab1 as a potential target for 8q gain in hepatocellular carcinoma. Carcinogenesis.

[3] Steinemann D, Skawran B, Becker T, Tauscher M, Weigmann A, Wingen L (2006). Assessment of differentiation and progression of hepatic tumors using array-based comparative genomic hybridization. Clin Gastroenterol Hepatol.

[4] Midorikawa Y, Yamamoto S, Ishikawa S, Kamimura N, Igarashi H, Sugimura H (2006). Molecular karyotyping of human hepatocellular carcinoma using single-nucleotide polymorphism arrays. Oncogene.

[5] Schlaeger C, Longerich T, Schiller C, Bewerunge P, Mehrabi A, Toedt G (2008). Etiology-dependent molecular mechanisms in human hepatocarcinogenesis. Hepatology.

[6] Chochi Y, Kawauchi S, Nakao M, Furuya T, Hashimoto K, Oga A (2009). A copy number gain of the 6p arm is linked with advanced hepatocellular carcinoma: an array-based comparative genomic hybridization study. J Pathol.

[7] Jia D, Wei L, Guo W, Zha R, Bao M, Chen Z (2011). Genome-wide copy number analyses identified novel cancer genes in hepatocellular carcinoma. Hepatology.

[8] Guichard C, Amaddeo G, Imbeaud S, Ladeiro Y, Pelletier L, Maad IB (2012). Integrated analysis of somatic mutations and focal copy-number changes identifies key genes and pathways in hepatocellular carcinoma. Nat Genet.

[9] Roessler S, Long EL, Budhu A, Chen Y, Zhao X, Ji J (2012). Integrative genomic identification of genes on 8p associated with hepatocellular carcinoma progression and patient survival. Gastroenterology.

[10] Wang K, Lim HY, Shi S, Lee J, Deng S, Xie T (2013). Genomic landscape of copy number aberrations enables the identification of oncogenic drivers in hepatocellular carcinoma. J Hepatology.

[11] Gu DL, Chen YH, Shih JH, Lin CH, Jou YS, Chen CF (2013). Target genes discovery through copy number alteration analysis in human hepatocellular carcinoma. World J Gastroenterol.

[12] Wang D, Zhu ZZ, Jiang H, Zhu J, Cong WM, Wen BJ (2015). Multiple genes identified as targets for 20q13.12–13.33 gain contributing to unfavorable clinical outcomes in patients with hepatocellular carcinoma. Hepatol Int..

[13] Cho HJ, Kim SS, Wang HJ, Kim BW, Cho H, Jung J (2016). Detection of novel genomic markers for predicting prognosis in hepatocellular carcinoma patients by integrative analysis of copy number aberrations and gene expression profiles: Results from a long-term follow-up. DNA Cell Biol.

[14] Yu MC, Lee CW, Lee YS, Lian JH, Tsai CL, Liu YP (2017). Prediction of early-stage hepatocellular carcinoma using OncoScan chromosomal copy number aberration data. World J Gastroenterol.

[15] Zhao K, Zhao Y, Zhu JY, Dong H, Cong WM, Yu Y (2018). A panel of genes identified as targets for 8q2413–243 gain contributing to unfavorable overall survival in patients with hepatocellular carcinoma. Curr Med Sci..

[16] Zhu ZZ, Bao LL, Zhao K, Xu Q, Zhu JY, Zhu KX (2019). Copy number aberrations of multiple genes identified as prognostic markers for extrahepatic metastasis-free survival of patients with hepatocellular carcinoma. Curr Med Sci.

[17] Qi LN, Li LQ, Chen YY, Chen ZH, Bai T, Xiang BD, et al. Genome-wide and differential proteomic analysis of hepatitis B virus and aflatoxin B1 related hepatocellular carcinoma in Guangxi, China. PLoS One. 2013;8(12):e83465. 10.1371/journal.pone.0083465 PMC3877066 24391771

[18] Liu YJ, Zhou Y, Yeh MM (2014). Recurrent genetic alterations in hepatitis C-associated hepatocellular carcinoma detected by genomic microarray: a genetic, clinical and pathological correlation study. Mol Cytogenet.

[19] Grommisch B, DiAdamo AJ, Xu ZY, XH Pan, DQ Ma, Xie JS, et al. Biobanking of residual specimens from diagnostic genetics laboratories: standard operating procedures, ethical and legal considerations, and research applications. N Am J Med Sci. 2013;6(4):200–7.

[20] Bajaj R, Xu F, Xiang B, Wilcox K, DiAdamo AJ, Kumar R (2011). Evidence-based genomic diagnosis characterized chromosomal and cryptic imbalances in 30 elderly patients with myelodysplastic syndrome and acute myeloid leukemia. Mol Cytogenet.

[21] Buza N, Xu F, Wu W, Car RJ, Li P, Hui P (2014). Recurrent chromosomal aberrations in intravenous leiomyomatosis of the uterus: High resolution array comparative genomic hybridization study. Hum Pathol.

[22] Ordulu Z, Chai H, Peng G, McDonald AG, De Nictolis M, Garcia-Fernandez E (2020). Molecular and clinicopathologic characterization of intravenous leiomyomatosis. Mod Pathol.

[23] Kaplan EL, Meier P (1958). Nonparametric estimation from incomplete observations. J Am Stat Assoc.

[24] Schoenfeld DA (1983). Sample-size formula for the proportional-harzards regression model. Biometrics.

[25] R Core Team. R: A Language and Environment for Statistical Computing 2019, Vienna, Austria.

[26] LIovet JM, Bru C, Bruix J. Prognosis of hepatocellular carcinoma: the BCLC staging classification. Semin Liver Dis. 1999;19(3):329–38. 10.1055/s-2007-1007122 10518312

[27] Pons F, Varela M, Llovet JM (2005). Staging systems in hepatocellular carcinoma. HPB (Oxford).

[28] Martins-Filho SN, Paiva C, Azevedo RS, Alves VAF (2017). Histological grading of hepatocellular carcinoma-A systematic review of literature. Front Med (Lausanne).

[29] Zhou L, Rui JA, Zhou WX, Wang SB, Chen SG, Qu Q (2017). Edmondson-steiner grade: a crucial predictor of recurrence and survival in hepatocellular carcinoma without microvascular invasio. Pathol Res Pract.

[30] Couture T, Amato K, DiAdamo A, Li P (2018). Jumping translocations of 1q in myelodysplastic syndrome and acute myeloid leukemia: Report of three cases and review of literature. Case Rep Genet.

[31] Zucman-Rossi J, Villanueva A, Nault JC, Llovet JM (2015). Genetic landscape and biomarkers of hepatocellular carcinoma. Gastroenterology.

[32] Cancer Genome Atlas Research Network (2017). Comprehensive and integrative genomic characterization of hepatocellular carcinoma. Cell.

[33] Parisi F, Ariyan S, Narayan D, Bacchiocchi A, Hoyt K, Cheng E (2011). Detecting copy number status and uncovering subclonal markers in heterogeneous tumor biopsies. BMC Genomics.

[34] Parisi F, Micsinai M, Strino F, Ariyan S, Narayan D, Bacchiocchi A (2012). Integrated analysis of tumor samples sheds light on tumor heterogeneity. Yale J Biol Med.

[35] Schulze K, Zucman-Rossi J (2015). Current issues on genomic heterogeneity in hepatocellular carcinoma and its implication in clinical practice. Hepat Oncol.

