# Supplementary material for: Correlating genomic copy number alterations with clinicopathologic findings in 75 cases of hepatocellular carcinoma
Source: BMC Med Genomics. 2021 Jun 8;14:150. doi: 10.1186/s12920-021-00998-9 (PMC8185937; doi:10.1186/s12920-021-00998-9)
Supplement: Supplementary file 1 — Additional file 1. Supplemental Method. Define Smallest Overlap Region (SOR) and calculate relative frequency (RF). Supplemental Figure 1. Survival difference between the cases with different recurrence and E-S grades in BCLC stage A and C. Supplemental Figure 2. The genomic profiles of CNAs in cases with different BCLC stages. Supplemental Figure 3. The genomic profiles of CNAs in cases with or without recurrence. Supplemental Figure 4. ROC curves for the prediction performance of percentage of genome change to E-S grades, recurrence, and BCLC stages. Supplemental Figure 5. The genomic profile of CNAs in different BCLC stages and E-S grades. Supplemental Figure 6. Clusters of the cases by CNAs in BCLC stage A. Supplemental Figure 7. Clusters of the cases by CNAs in BCLC stage C. Supplemental Figure 8. Survival difference between different clusters in all cases and cases in BCLC stages A and C. [file 12920_2021_998_MOESM1_ESM.pdf]

Supplemental Method

Define Smallest Overlap Region (SOR) and calculate relative frequency

Copy number aberrations (CNAs) have different size and location for different patients. It is difficult to make a direct comparison between patients with different CNAs. For example, when we compare CNVs (blue bars) between S1 and S2 shown below, we cannot compare the two CNAs in S1 to the one CNV in S2. The overlap regions between the two patients are usually used to make a direct comparison. The problem becomes more complex when there are multiple patients. Here we defined the smallest overlap region as the smallest section overlapped by CNAs from all patients. To simplify the problem, SOR also includes the region unique to a single sample, which is kind of region "overlapped" by itself like the first SOR shown below. For each SOR, we can calculate the relative frequency of CNA as the number of cases with CNA in the SOR divided by the number of all cases and make a comparison between patients. The figure below shows an example of how we define SORs for 5 cases. The number at the bottom of the figure shows the relative frequency for each SOR.

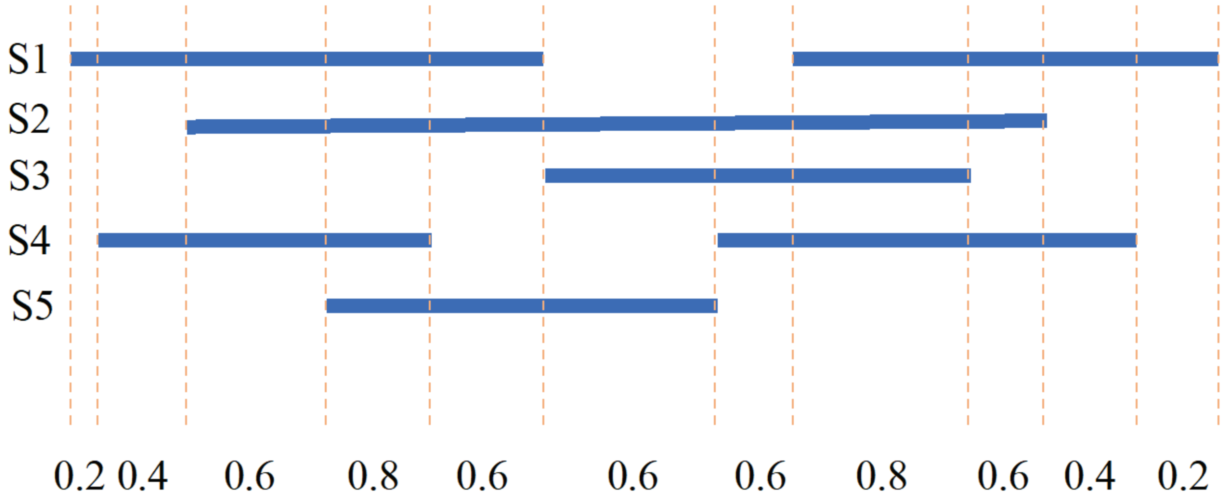

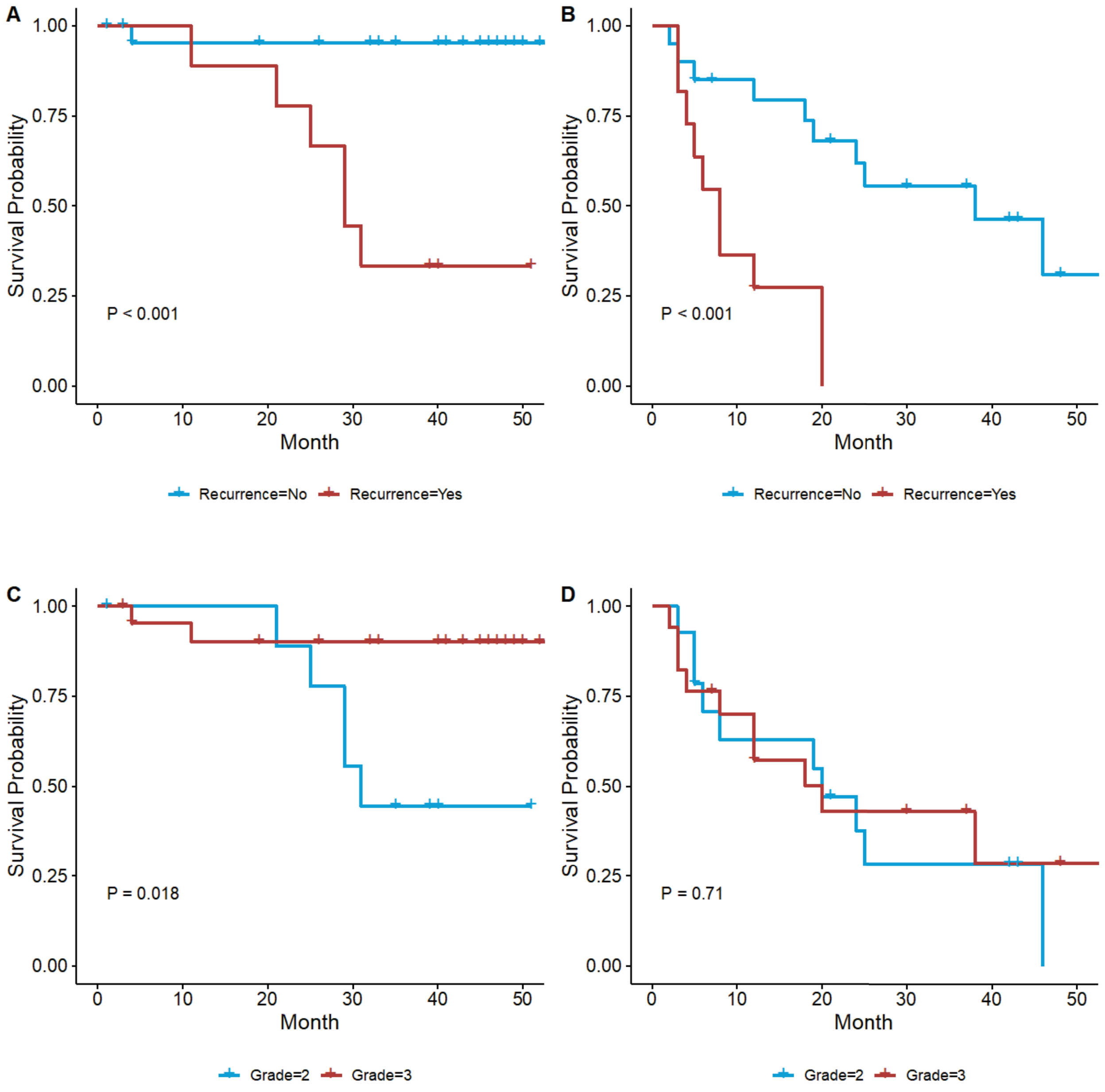

Supplemental Figure 1. Survival difference between the cases with different recurrence and E-S grades in BCLC stage A and C.

A) Survival difference between the cases with and without recurrence in BCLC stage A. B) Survival difference between the cases with and without recurrence in BCLC stage C. C) Survival difference between the cases of E-S grades II and III in BCLC stage A. D) Survival difference between the cases of E-S grades II and III in BCLC stage C.

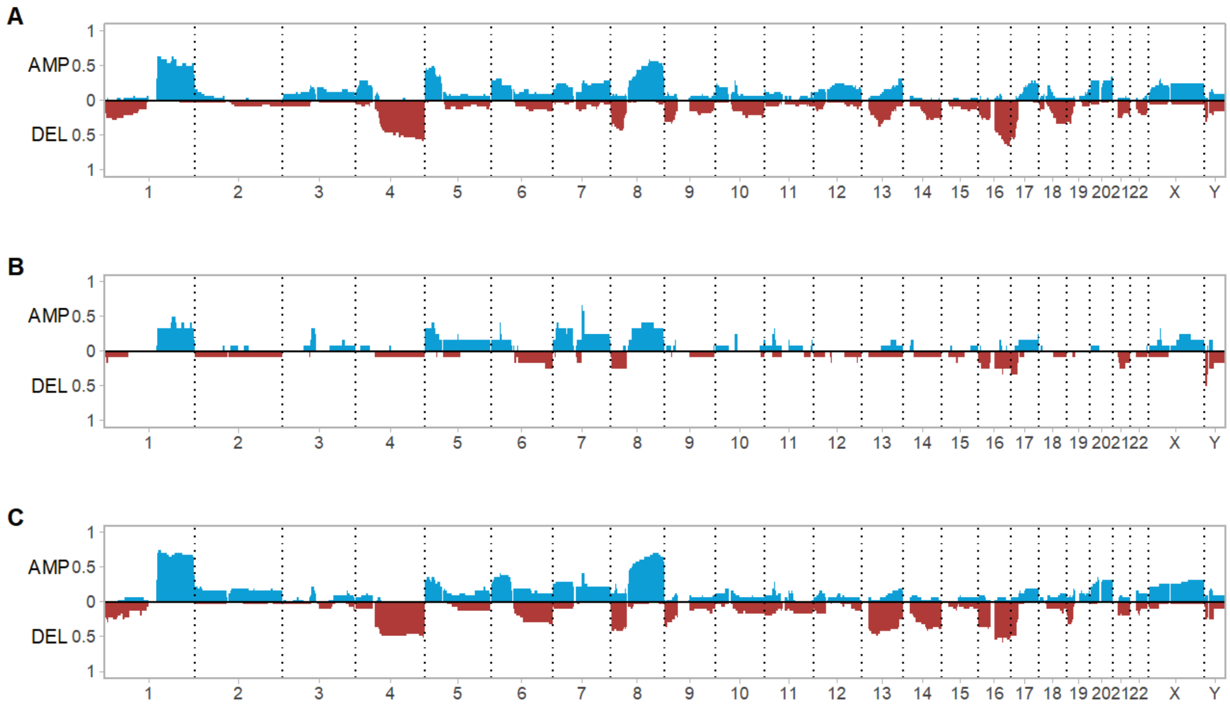

Supplemental Figure 2. The genomic profiles of CNAs in cases with different BCLC stages. A) Cases in BCLC stage A (n=32). B) Cases in BCLC stage B (n=12). C) Cases in BCLC stage C (n=31).

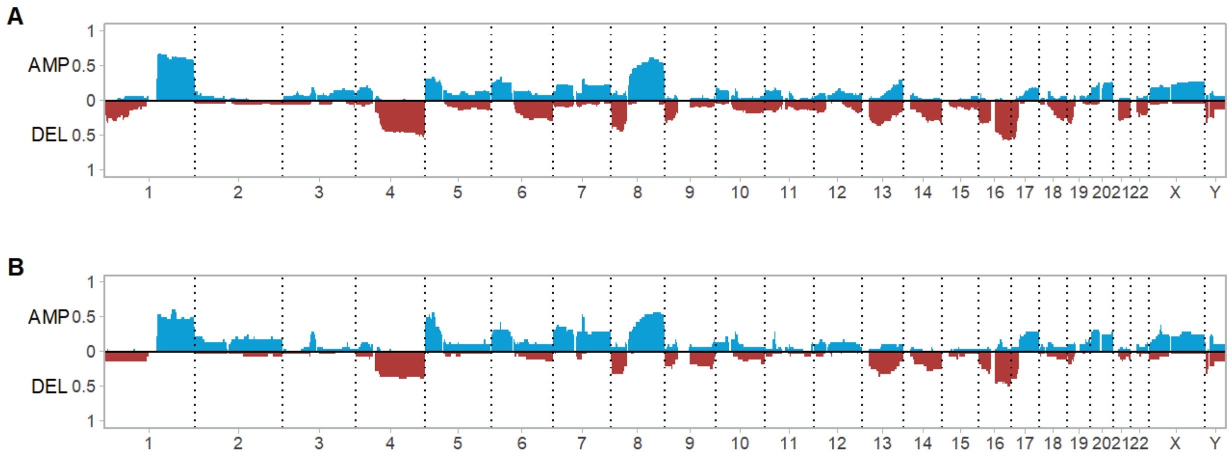

Supplemental Figure 3. The genomic profiles of CNAs in cases with or without recurrence. A) Cases without recurrence (n=47). B) Cases with recurrence (n=28).

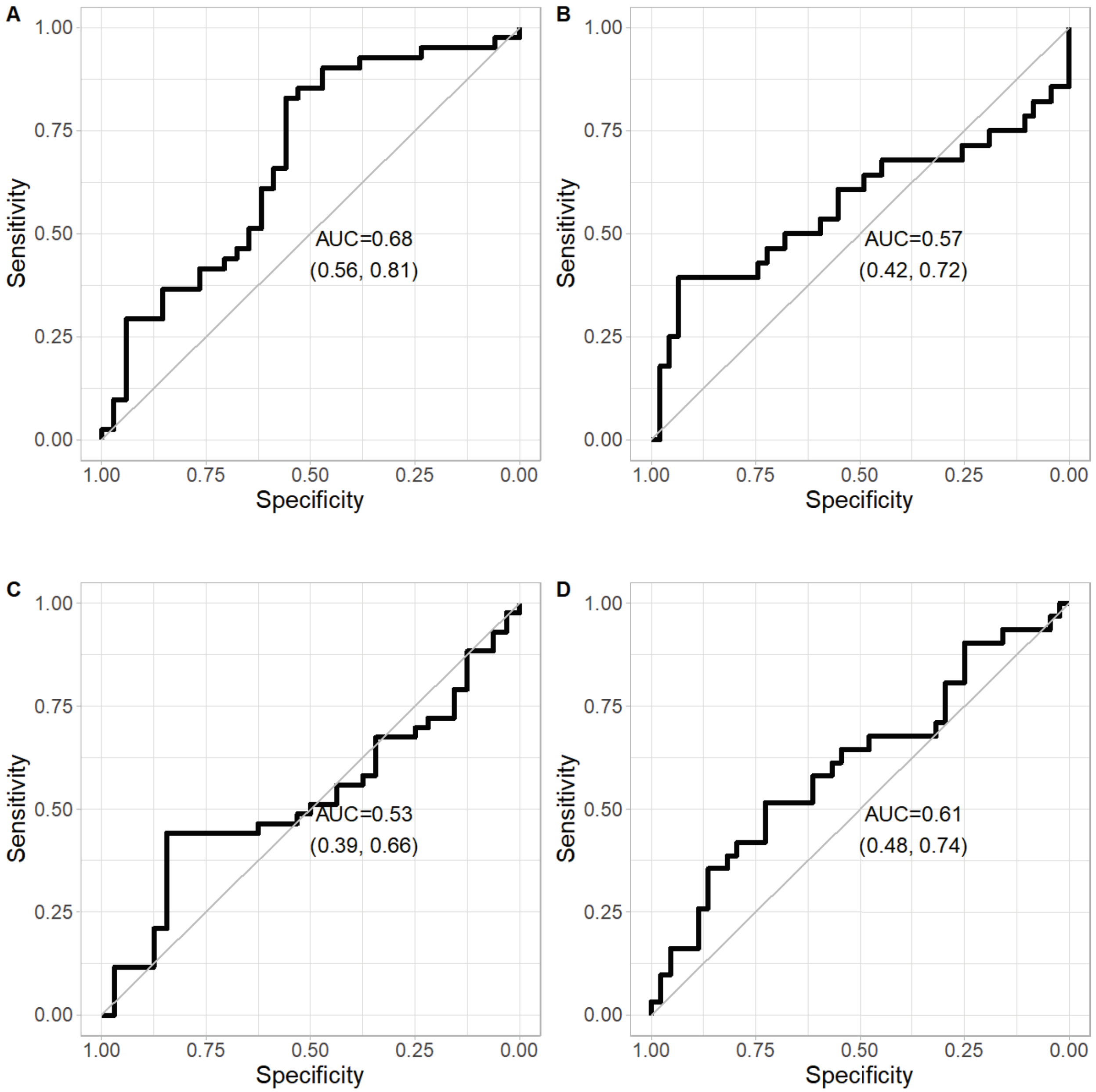

Supplemental Figure 4. ROC curves for the prediction performance of percentage of genome change to E-S grades, recurrence, and BCLC stages.

Prediction ability of percentage of genome change to A) E-S grades II and III, B) recurrence vs non-recurrence, C) BCLC stage A versus stage B+C, D) stage A+B versus stage C. The numbers in the parentheses are the 95% confidence interval of area under the curve (AUC).

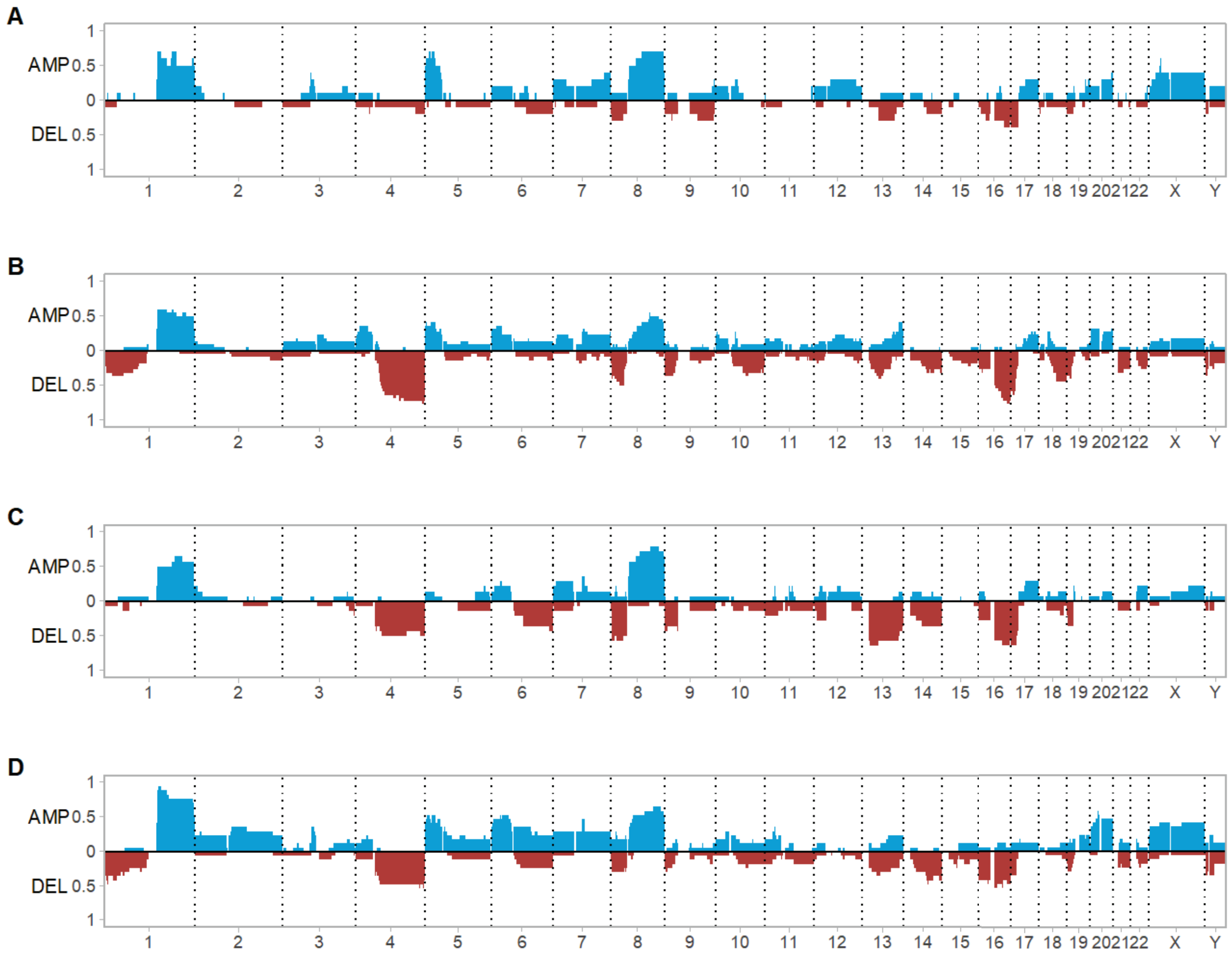

Supplemental Figure 5. The genomic profiles of CNAs in different BCLC stages and E-S grades. A) Cases in BCLC stage A and E-S grade II (n=10). B) Cases in BCLC stage A and E-S grade III (n=22). C) Cases in BCLC stage C and E-S grade II (n=14). D) Cases in BCLC stage C and E-S grade III (n=17).

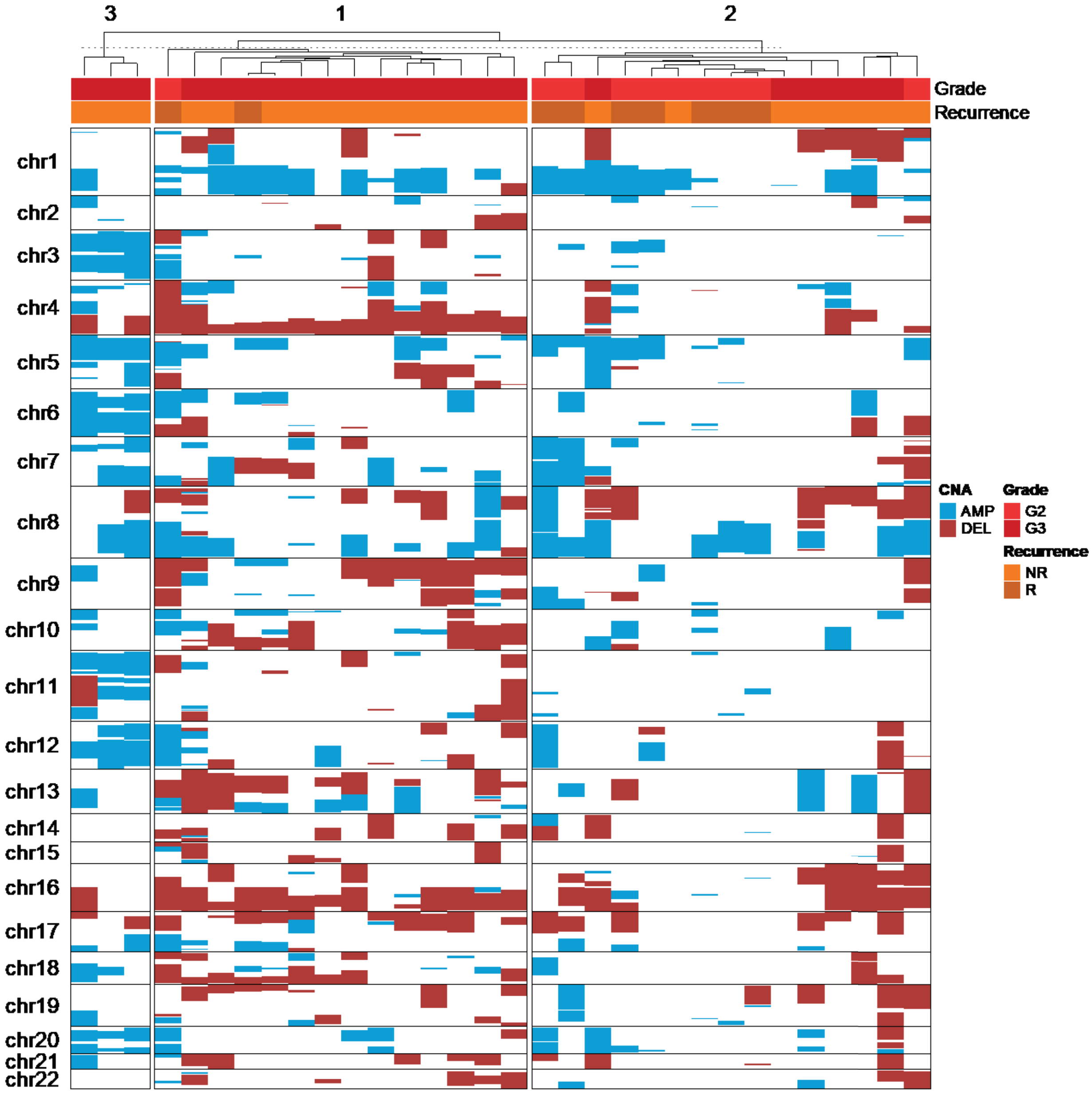

Supplemental Figure 6. Clusters of cases by CNAs in BCLC stage A.

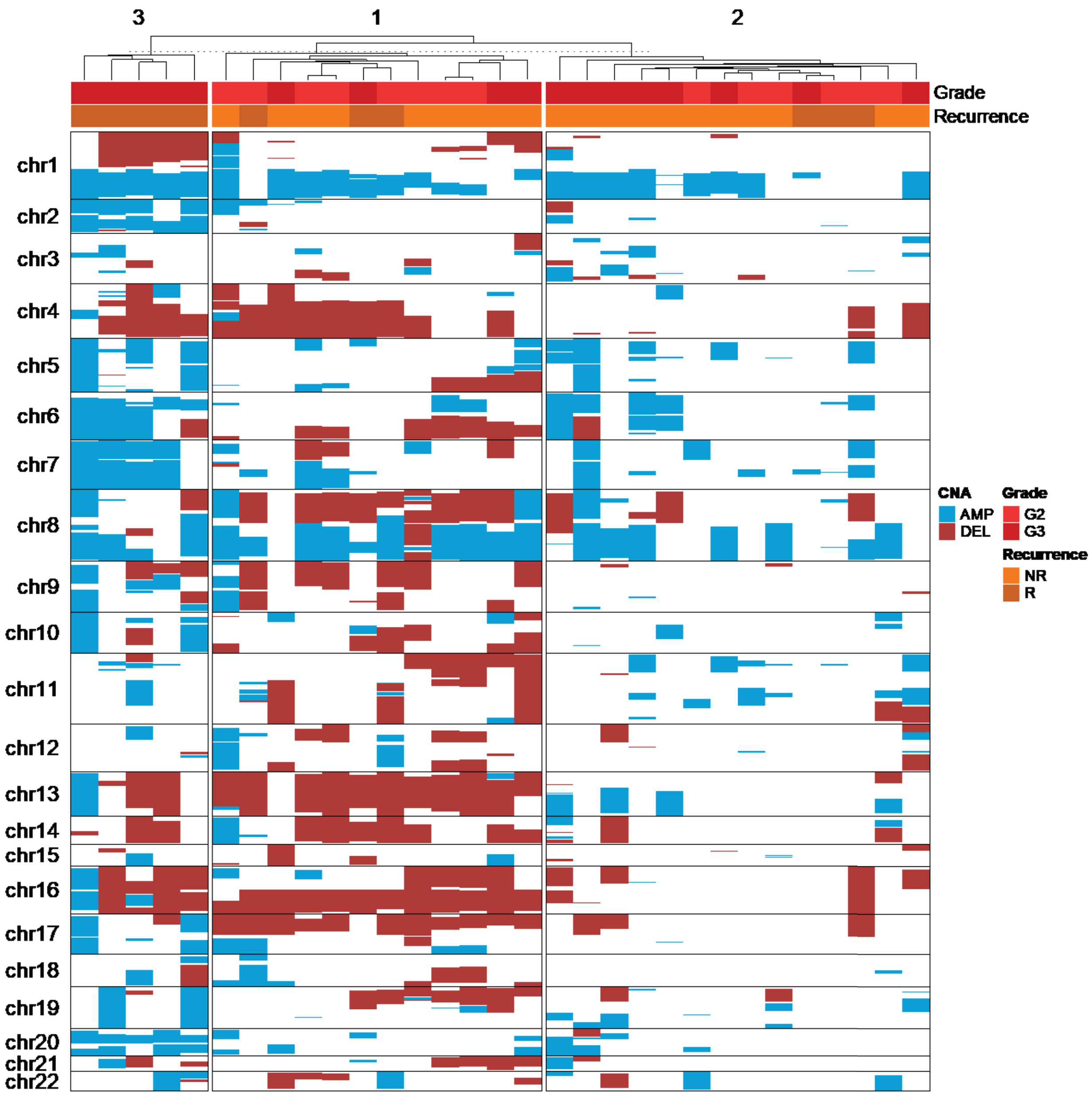

Supplemental Figure 7. Clusters of cases by CNAs in BCLC stage C.

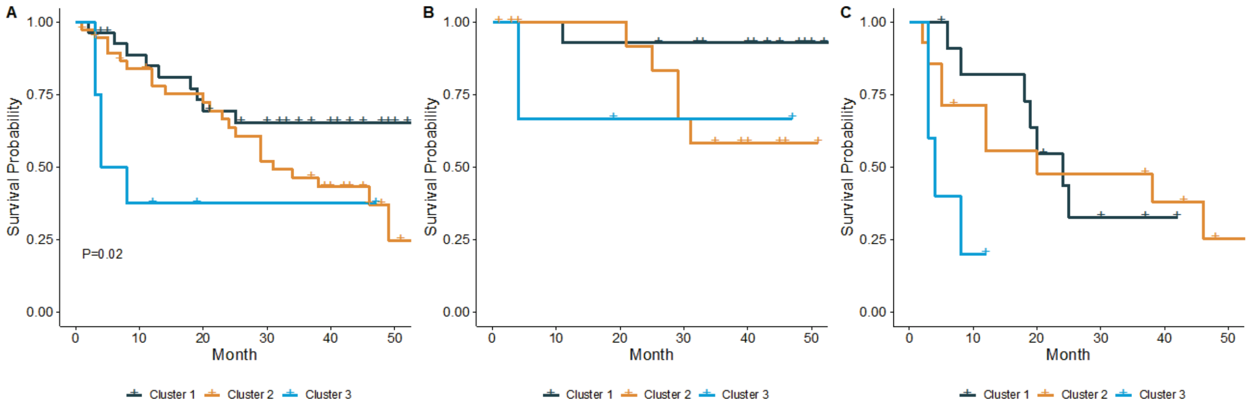

Supplemental Figure 8. Survival difference between different clusters in all cases and cases in BCLC stages A and C. A) All cases. B) Cases of BCLC stage A. C) Cases of BCLC stage C.
