# Supplementary material for: Correlating genomic copy number alterations with clinicopathologic findings in 75 cases of hepatocellular carcinoma
Source: BMC Med Genomics. 2021 Jun 8;14:150. doi: 10.1186/s12920-021-00998-9 (PMC8185937; doi:10.1186/s12920-021-00998-9)
Supplement: Supplementary file 2 — Additional file 2. Supplemental Table 1. CNAs detected from 75 cases of HCC. Supplemental Table 2. List of candidate and key genes in recurrent CNAs for HCC. [file 12920_2021_998_MOESM2_ESM.pdf]

Supplemental Table 1. CNAs detected from 75 cases of hepatocellular carcinoma

Case ID	Chr	Start	Stop	Cytoband	Size(kb)	Type	Mean Log Ratio
001	chr1	749625	115563570	p36.33 - p13.2	114813.946	Loss	-0.44145423
	chr1	118214256	120331248	p12	2116.993	Loss	-0.6356078
	chr1	144124745	246751532	q21.1 - q44	102626.788	Gain	0.64473027
	chr2	32444	89649697	p25.3 - p11.2	89617.254	Gain	0.3248169
	chr2	94999687	242690037	q11.1 - q37.3	147690.351	Gain	0.35368505
	chr4	64471364	190588045	q13.1 - q35.2	126116.682	Loss	-0.435663
	chr5	1852098	45865412	p15.33 - p11	44013.315	Gain	0.38034487
	chr5	50058977	180417981	q11.1 - q35.3	130359.005	Gain	0.27656764
	chr6	5738643	54239060	p25.1 - p12.1	48500.418	Gain	0.76706034
	chr6	65760879	170382576	q12 - q27	104621.698	Loss	-0.29449388
	chr8	211611	36865972	p23.3 - p12	36654.362	Loss	-0.42734212
	chr8	39128088	43452795	p11.23 - p11.1	4324.708	Gain	0.63472795
	chr8	47987961	145148886	q11.1 - q24.3	97160.926	Gain	0.66316336
	chr9	261257	29908802	p24.3 - p21.1	29647.546	Deletion	-1.3987811
	chr9	70327267	134370152	q13 - q34.13	64042.886	Loss	-0.44710913
	chr9	134397168	139998682	q34.13 - q34.3	5601.515	Gain	0.39457786
	chr10	27501733	38523886	p12.1 - p11.21	11022.154	Gain	0.43332583
	chr10	42289771	134882384	q11.21 - q26.3	92592.614	Gain	0.36380044
	chr12	73657187	78558323	q21.1 - q21.2	4901.137	Loss	-0.49836844
	chr12	81276064	86525837	q21.31 - q21.32	5249.774	Gain	0.5403453
	chr16	46271	34903000	p13.3 - p11.1	34856.73	Loss	-0.72094125
	chr16	45284566	88563897	q11.2 - q24.3	43279.332	Loss	-0.4342219
	chr17	148092	20853231	p13.3 - p11.2	20705.14	Gain	0.34634578
	chr17	24795468	78586290	q11.2 - q25.3	53790.823	Gain	0.34262127
	chr18	518074	14918854	p11.32 - p11.21	14400.781	Gain	0.42860472
	chr18	17760071	76083117	q11.2 - q23	58323.047	Loss	-0.43560392
	chr19	318892	24132581	p13.3 - p12	23813.69	Gain	0.33878547
	chr19	32964337	63672832	q12 - q13.43	30708.496	Gain	0.33090895
	chr20	1528899	25611675	p13 - p11.21	24082.777	Gain	0.3994832
	chr20	29423641	62134908	q11.21 - q13.33	32711.268	Gain	0.34768236
	chr21	23680979	46433516	q21.2 - q22.3	22752.538	Loss	-0.3645308
	chr22	15476855	25755261	q11.1 - q12.1	10278.407	Gain	0.3146128
	chr22	25857188	30160421	q12.1 - q12.2	4303.234	Loss	-0.39801207
	chrX	1091	57178479	p22.33 - p11.1	57177.389	Loss	-0.4899994
	chrX	839417	2662039	p22.33	1822.623	Deletion	-1.0468109
	chrX	20125720	20184734	p22.12	59.015	Deletion	-2.5918598
	chrX	61848414	154886101	q11.1 - q28	93037.688	Loss	-0.37334722
	chrY	1091	2666392	p11.32 - p11.31	2665.302	Loss	-0.95641464
	chrY	2716461	10511314	p11.31 - p11.2	7794.854	Deletion	-2.8189662
	chrY	12571053	57586758	q11.21 - q12	45015.706	Deletion	-2.086531
002	chr1	7695956	8957695	p36.23	1261.74	Gain	1.7660244
	chr1	144127702	245804497	q21.1 - q44	101676.796	Gain	0.61560005
	chr2	32444	84037584	p25.3 - p11.2	84005.141	Gain	0.3413675
	chr3	36460084	87917144	p22.3 - p11.2	51457.061	Gain	0.31065568
	chr3	95136554	196542278	q11.2 - q29	101405.725	Gain	0.28707322
	chr4	12322518	47810101	p15.33 - p12	35487.584	Gain	0.51970613
	chr4	53426490	67133381	q12 - q13.2	13706.892	Gain	0.52424675
	chr4	67425960	190706331	q13.2 - q35.2	123280.372	Loss	-0.4485348
	chr5	204737	45865412	p15.33 - p11	45660.676	Gain	0.8751743

	chr5	50058977	60345508	q11.1 - q12.1	10286.532	Gain	0.5562959
	chr5	97818174	118249997	q21.1 - q23.1	20431.824	Gain	0.30665928
	chr6	593433	58506751	p25.3 - p11.1	57913.319	Gain	0.722913
	chr6	62040890	170732033	q11.1 - q27	108691.144	Gain	0.7381321
	chr7	10212848	55458325	p21.3 - p11.2	45245.478	Gain	0.2898288
	chr9	8832502	36090542	p24.1 - p13.3	27258.041	Gain	0.38030395
	chr10	138206	38468053	p15.3 - p11.21	38329.848	Gain	0.78235275
	chr10	45376528	55595537	q11.21 - q21.1	10219.01	Gain	0.35569757
	chr11	551995	46490960	p15.5 - p11.2	45938.966	Gain	0.8901707
	chr11	46594346	49489219	p11.2 - p11.12	2894.874	Gain	0.4711205
	chr11	55002923	96617014	q11 - q21	41614.092	Loss	-0.45645523
	chr11	97126209	133886901	q22.1 - q25	36760.693	Gain	0.71466464
	chr12	37331742	108736639	q12 - q24.11	71404.898	Gain	0.2569092
	chr13	61326326	111978171	q21.31 - q34	50651.846	Gain	0.32613832
	chr16	45122058	88638764	q11.2 - q24.3	43516.707	Loss	-0.4699327
	chr17	87009	13498264	p13.3 - p12	13411.256	Loss	-0.5816262
	chr17	45570882	78623230	q21.33 - q25.3	33052.349	Gain	0.52393186
	chr18	18016879	75320815	q11.2 - q23	57303.937	Gain	0.3219037
	chr19	33099182	63784382	q12 - q13.43	30685.201	Gain	0.2603796
	chr20	1056046	26023841	p13 - p11.1	24967.796	Gain	0.7056149
	chr20	29501535	62363633	q11.21 - q13.33	32862.099	Gain	0.5454023
	chr21	14510897	46892352	q11.2 - q22.3	32381.456	Gain	0.5781066
	chrX	5891860	58068490	p22.32 - p11.1	52176.631	Gain	0.73756015
	chrX	62181725	154886101	q11.1 - q28	92704.377	Gain	0.7618302
	chrY	2783745	10622121	p11.31 - p11.2	7838.377	Loss	-0.6061363
	chrY	17284587	57745301	q11.221 - q12	40460.715	Gain	0.6656385
003	Normal						
004	Normal						
005	Normal						
006	chr1	21471020	94148139	p36.12 - p22.1	72677.12	Loss	-0.25191522
	chr1	143787504	172734850	q21.1 - q25.1	28947.347	Gain	0.746482
	chr3	134711	42048759	p26.3 - p22.1	41914.049	Gain	0.7508471
	chr4	418732	48350883	p16.3 - p12	47932.152	Gain	1.0556623
	chr4	53178040	54554612	q12	1376.573	Gain	0.5792713
	chr4	55573918	57372145	q12	1798.228	Gain	0.4530955
	chr4	57472343	191133668	q12 - q35.2	133661.326	Loss	-0.27780762
	chr5	14875036	43330652	p15.2 - p12	28455.617	Gain	0.8000573
	chr6	115426	30081187	p25.3 - p21.33	29965.762	Gain	0.8100357
	chr6	65850743	170732033	q12 - q27	104881.291	Loss	-0.41303226
	chr7	6837409	40884093	p22.1 - p14.1	34046.685	Gain	0.41687828
	chr7	117269869	128200133	q31.31 - q32.1	10930.265	Loss	-0.8002011
	chr7	139463387	158554347	q34 - q36.3	19090.961	Loss	-0.39833918
	chr8	432267	4239024	p23.3 - p23.2	3806.758	Loss	-0.9304533
	chr8	4288734	10511405	p23.2 - p23.1	6222.672	Gain	0.3063008
	chr8	10917417	36481098	p23.1 - p12	25563.682	Loss	-0.7847145
	chr8	39378051	40879554	p11.23 - p11.21	1501.504	Gain	0.4983334
	chr8	60800114	70906621	q12.1 - q13.3	10106.508	Loss	-0.26256827
	chr8	71233042	146201771	q13.3 - q24.3	74968.73	Gain	0.6040126
	chr9	363868	19286158	p24.3 - p22.1	18922.291	Loss	-0.26148772
	chr9	19501524	23779216	p22.1 - p21.3	4277.693	Deletion	-1.6886952
	chr9	28660476	38653271	p21.1 - p13.1	9992.796	Gain	0.34845057
	chr10	42209250	57734841	q11.21 - q21.1	15525.592	Gain	0.37589985

	chr10	81692910	90594643	q22.3 - q23.31	8901.734	Loss	-0.4104324
	chr10	128457030	135222482	q26.2 - q26.3	6765.453	Loss	-0.87384915
	chr11	27548931	46490960	p14.1 - p11.2	18942.03	Gain	0.33648255
	chr11	95882988	116439451	q21 - q23.3	20556.464	Gain	0.7289841
	chr11	116687865	120565106	q23.3	3877.242	Gain	0.34886903
	chr11	120814012	134373617	q24.1 - q25	13559.606	Loss	-0.38735718
	chr12	8650725	14666257	p13.31 - p13.1	6015.533	Loss	-0.40863332
	chr12	14740410	34081210	p13.1 - p11.1	19340.801	Gain	0.35953647
	chr12	58741161	85149014	q14.1 - q21.32	26407.854	Gain	0.36274034
	chr12	124390826	130793291	q24.31 - q24.33	6402.466	Gain	0.52040374
	chr13	18194544	114077122	q11 - q34	95882.579	Loss	-0.35948464
	chr13	51544169	73896018	q14.3 - q22.1	22351.85	Loss	-0.7765361
	chr14	60720314	71888587	q23.1 - q24.2	11168.274	Loss	-0.82702076
	chr14	75098864	84804155	q24.3 - q31.3	9705.292	Loss	-0.40357736
	chr14	97298383	100435026	q32.2 - q32.31	3136.644	Gain	0.53962183
	chr14	101201692	105632227	q32.31 - q32.33	4430.536	Loss	-0.7087441
	chr15	21409583	78069743	q11.2 - q25.1	56660.161	Loss	-0.32183012
	chr15	78602427	100200996	q25.1 - q26.3	21598.57	Gain	0.62899476
	chr16	45122058	88638764	q11.2 - q24.3	43516.707	Loss	-0.36683983
	chr17	32069339	43173798	q12 - q21.32	11104.46	Gain	0.30348775
	chr17	64695529	70391195	q24.3 - q25.1	5695.667	Gain	0.28238574
	chr18	226820	14918854	p11.32 - p11.21	14692.035	Loss	-0.32910684
	chr18	46165567	76083117	q21.1 - q23	29917.551	Loss	-0.62586385
	chr19	557030	18058366	p13.3 - p13.11	17501.337	Loss	-0.41516617
	chr21	13562263	46646783	q11.2 - q22.3	33084.521	Loss	-0.30130348
	chr22	17299942	21328331	q11.21 - q11.22	4028.39	Gain	0.35392728
	chr22	21631426	49168537	q11.22 - q13.33	27537.112	Loss	-0.37166533
	chrX	2719027	55256168	p22.33 - p11.21	52537.142	Deletion	-1.1331073
	chrX	2754701	9349157	p22.33 - p22.31	6594.457	Deletion	-1.3735967
	chrX	48864380	48911097	p11.23	46.718	Gain	0.3482219
	chrX	63426744	154561665	q11.1 - q28	91134.922	Loss	-0.57045054
	chrX	65521790	69476037	q12 - q13.1	3954.248	Deletion	-1.1877627
	chrX	74149292	85140776	q13.3 - q21.2	10991.485	Deletion	-1.0964737
	chrX	85286931	153943360	q21.2 - q28	68656.43	Loss	-0.48205677
	chrY	2716461	10511314	p11.31 - p11.2	7794.854	plification	3.512802
	chrY	12571053	26957873	q11.21 - q11.23	14386.821	plification	2.6007063
007	chr1	749625	78209946	p36.33 - p31.1	77460.322	Loss	-0.34692383
	chr1	144188986	246630459	q21.1 - q44	102441.474	Gain	0.35540584
	chr4	3496955	48527470	p16.2 - p12	45030.516	Gain	0.39950645
	chr4	52472013	61545140	q12 - q13.1	9073.128	Gain	0.37585655
	chr4	62563623	191004108	q13.1 - q35.2	128440.486	Loss	-0.37095103
	chr8	211611	36134051	p23.3 - p12	35922.441	Loss	-0.59758466
	chr10	52897465	135254513	q11.23 - q26.3	82357.049	Gain	0.31897083
	chr16	94314	34903000	p13.3 - p11.1	34808.687	Loss	-0.3408102
	chr16	45122058	88621982	q11.2 - q24.3	43499.925	Loss	-0.33435038
	chr17	148092	14641708	p13.3 - p12	14493.617	Loss	-0.3279481
	chrX	890703	58068490	p22.33 - p11.1	57177.788	Gain	0.47116855
	chrX	61848414	154407365	q11.1 - q28	92558.952	Gain	0.50305486
	chrY	2882209	10511314	p11.31 - p11.2	7629.106	Deletion	-1.5827075
	chrY	12593244	27069824	q11.21 - q11.23	14476.581	Loss	-0.93082124
008	chr1	184912161	196226519	q31.1 - q31.3	11314.359	Gain	0.27106306
	chr3	74963465	90336752	p12.3 - p11.1	15373.288	Gain	0.2811857

	chr5	580552	39775231	p15.33 - p13.1	39194.68	Gain	0.2618596
	chr7	78549068	86205180	q21.11 - q21.12	7656.113	Gain	0.3064506
	chr13	52839564	96289044	q21.1 - q32.1	43449.481	Gain	0.2579189
	chr16	57785182	64970934	q21	7185.753	Gain	0.33826977
	chrX	85286931	118106800	q21.2 - q24	32819.87	Gain	0.4481286
	chrY	218292	2372192	p11.32 - p11.31	2153.901	Gain	0.36763462
	chrY	2783745	7788655	p11.31 - p11.2	5004.911	Loss	-0.5103464
009	chr3	40200214	42280450	p22.1	2080.237	Gain	0.521732
	chr3	77222495	90336752	p12.3 - p11.1	13114.258	Gain	0.3333027
	chr5	17960602	29407781	p15.1 - p13.3	11447.18	Gain	0.2598866
	chr7	8838768	16688974	p21.3 - p21.1	7850.207	Gain	0.26189417
	chr7	78549068	86412277	q21.11 - q21.12	7863.21	Gain	0.34967968
	chr9	30510898	33293292	p21.1 - p13.3	2782.395	Gain	0.65433234
	chr10	52702648	61282457	q11.23 - q21.2	8579.81	Gain	0.26377216
	chr11	21166615	29151796	p15.1 - p14.1	7985.182	Gain	0.26793832
	chrX	26122426	37420242	p21.3 - p21.1	11297.817	Gain	0.2750433
	chrY	2716461	7788655	p11.31 - p11.2	5072.195	Loss	-0.5367243
010	chr1	143639135	247179291	q21.1 - q44	103540.157	Gain	1.1449513
	chr2	74669	89057332	p25.3 - p11.2	88982.664	Gain	0.40250662
	chr2	94892766	242535059	q11.1 - q37.3	147642.294	Gain	0.4435264
	chr3	77222495	90336752	p12.3 - p11.1	13114.258	Gain	0.31429344
	chr4	58720843	68288131	q12 - q13.2	9567.289	Gain	0.3642861
	chr5	260981	46136124	p15.33 - p11	45875.144	Gain	0.7872047
	chr5	49725929	180517872	q11.1 - q35.3	130791.944	Gain	0.7085161
	chr6	2046381	58722020	p25.3 - p11.1	56675.64	Gain	0.39853698
	chr6	62040890	170707926	q11.1 - q27	108667.037	Gain	0.44174019
	chr7	524935	57498383	p22.3 - p11.1	56973.449	Gain	0.71902514
	chr7	62291739	158781397	q11.21 - q36.3	96489.659	Gain	0.7366156
	chr8	283177	41809596	p23.3 - p11.21	41526.42	Gain	0.47035608
	chr8	48437368	56590706	q11.21 - q12.1	8153.339	Gain	0.53615534
	chr8	58358356	146201771	q12.1 - q24.3	87843.416	Gain	1.3623095
	chr9	7131937	35097397	p24.1 - p13.3	27965.461	Gain	0.51510435
	chr9	67911945	140128736	q12 - q34.3	72216.792	Gain	0.3757073
	chr10	138206	38585934	p15.3 - p11.21	38447.729	Gain	0.692291
	chr10	42209250	135254513	q11.21 - q26.3	93045.264	Gain	0.70585936
	chr13	18424064	114077122	q12.11 - q34	95653.059	Gain	0.46091276
	chr13	52839564	71377730	q21.1 - q21.33	18538.167	Gain	0.7100086
	chr14	62958522	81714630	q23.2 - q31.1	18756.109	Loss	-0.59121865
	chr16	211735	34903000	p13.3 - p11.1	34691.266	Gain	0.36164054
	chr16	45282156	88523261	q11.2 - q24.3	43241.106	Gain	0.37238246
	chr17	544452	22129948	p13.3 - p11.1	21585.497	Gain	0.43440875
	chr17	22715254	78623230	q11.1 - q25.3	55907.977	Gain	0.37161994
	chr20	257960	26023841	p13 - p11.1	25765.882	Gain	0.3773818
	chr20	31085455	62363633	q11.21 - q13.33	31278.179	Gain	0.35741493
	chrX	1529	58068490	p22.33 - p11.1	58066.962	Gain	0.7536007
	chrX	61848414	154886101	q11.1 - q28	93037.688	Gain	0.78719664
	chrY	109064	2622294	p11.32 - p11.31	2513.231	Gain	0.8063906
	chrY	13010597	57745301	q11.21 - q12	44734.705	Gain	0.3579176
011	chr1	749625	116112500	p36.33 - p13.1	115362.876	Loss	-0.5226555
	chr1	144219515	247179291	q21.1 - q44	102959.777	Gain	0.7997789
	chr2	111638161	242690037	q13 - q37.3	131051.877	Gain	0.46932757
	chr4	61552	48182792	p16.3 - p12	48121.241	Gain	0.4621316

	chr4	53178040	190706331	q12 - q35.2	137528.292	Loss	-0.4827993
	chr6	1786686	49257051	p25.3 - p12.3	47470.366	Gain	0.27206054
	chr7	187615	57327440	p22.3 - p11.2	57139.826	Gain	0.45836988
	chr7	62153588	158781397	q11.21 - q36.3	96627.81	Gain	0.47310844
	chr9	856525	22755281	p24.3 - p21.3	21898.757	Loss	-0.5292394
	chr9	23186282	39146954	p21.3 - p13.1	15960.673	Gain	0.49961016
	chr11	26677538	28867117	p14.2 - p14.1	2189.58	Gain	0.4078228
	chr13	18194544	114077122	q11 - q34	95882.579	Loss	-0.5134615
	chr14	25985334	105399038	q12 - q32.33	79413.705	Loss	-0.5266935
	chr16	94314	34903000	p13.3 - p11.1	34808.687	Loss	-0.54065764
	chr16	45122058	88638764	q11.2 - q24.3	43516.707	Loss	-0.5212033
	chr17	87009	15589301	p13.3 - p12	15502.293	Loss	-0.50933576
	chr20	118826	25680554	p13 - p11.21	25561.729	Gain	0.48976272
	chr20	29423641	62363633	q11.21 - q13.33	32939.993	Gain	0.35110706
	chr22	15476855	49525130	q11.1 - q13.33	34048.276	Gain	0.38178176
	chrX	31285077	34058518	p21.2 - p21.1	2773.442	Gain	0.35936925
	chrY	2783745	10511314	p11.31 - p11.2	7727.57	Loss	-0.90252846
	chrY	12593244	57586758	q11.21 - q12	44993.515	Loss	-0.6300148
012	chr1	144127702	213042600	q21.1 - q41	68914.899	Gain	0.5644681
	chr3	95295727	136777035	q11.2 - q22.2	41481.309	Loss	-0.30170354
	chr3	137793401	195461874	q22.3 - q29	57668.474	Gain	0.3222863
	chr4	66267986	191133668	q13.1 - q35.2	124865.683	Loss	-0.6888587
	chr6	65760879	170732033	q12 - q27	104971.155	Loss	-0.6596399
	chr7	290112	55242365	p22.3 - p11.2	54952.254	Gain	0.26150572
	chr8	211611	10274974	p23.3 - p23.1	10063.364	Deletion	-1.5260378
	chr8	10323367	15193518	p23.1 - p22	4870.152	Gain	1.0864851
	chr8	15407172	32499834	p22 - p12	17092.663	Deletion	-1.2948453
	chr8	30186270	30686931	p12	500.662	Deletion	-2.1176136
	chr8	36973293	43452795	p12 - p11.1	6479.503	Loss	-0.45996934
	chr8	48067862	105669346	q11.1 - q22.3	57601.485	Loss	-0.2735872
	chr8	106883443	130857683	q23.1 - q24.21	23974.241	Gain	0.34979212
	chr8	131720610	146250824	q24.22 - q24.3	14530.215	Loss	-0.3021185
	chr9	261257	39091408	p24.3 - p13.1	38830.152	Loss	-0.6657919
	chr10	43210109	78314145	q11.21 - q22.3	35104.037	Loss	-0.6715864
	chr11	208365	36930558	p15.5 - p12	36722.194	Loss	-0.25456953
	chr13	21261016	103076962	q12.11 - q33.1	81815.947	Loss	-0.6793598
	chr14	35543573	105399038	q13.2 - q32.33	69855.466	Loss	-0.7001631
	chr16	46271	34903000	p13.3 - p11.1	34856.73	Loss	-0.7098384
	chr16	45122058	88638764	q11.2 - q24.3	43516.707	Loss	-0.693043
	chr17	87009	22002704	p13.3 - p11.2	21915.696	Loss	-0.7028461
	chr17	22427573	39014573	q11.1 - q21.31	16587.001	Loss	-0.69777066
	chr18	56668527	76083117	q21.32 - q23	19414.591	Loss	-0.64062065
	chr19	278273	14230645	p13.3 - p13.12	13952.373	Loss	-0.69291365
	chr19	15062885	16299294	p13.12 - p13.11	1236.41	Gain	0.39682245
	chr19	16299299	16788515	p13.11	489.217	Loss	-0.87447184
	chrX	110071615	154561665	q22.3 - q28	44490.051	Gain	0.5332049
	chrY	1091	2662039	p11.32 - p11.31	2660.949	Loss	-0.5384299
013	chr1	749625	120386946	p36.33 - p12	119637.322	Loss	-0.39986706
	chr1	144127702	246751532	q21.1 - q44	102623.831	Gain	0.8353704
	chr2	32444	91129998	p25.3 - p11.1	91097.555	Gain	0.26570696
	chr2	102379623	215128693	q12.1 - q35	112749.071	Gain	0.5913274
	chr2	215430915	242053993	q35 - q37.3	26623.079	Loss	-0.34861258

	chr3	73168400	90336752	p13 - p11.1	17168.353	Gain	0.30277213
	chr3	176437712	182915023	q26.31 - q26.33	6477.312	Gain	0.3222648
	chr4	16509695	35612378	p15.32 - p14	19102.684	Gain	0.27575034
	chr4	42663317	47810101	p13 - p12	5146.785	Gain	0.35932422
	chr4	52383858	54819288	q11 - q12	2435.431	Loss	-0.471689
	chr4	66871839	189257416	q13.2 - q35.2	122385.578	Loss	-0.34520623
	chr5	16989989	29407781	p15.1 - p13.3	12417.793	Gain	0.33981436
	chr5	77693995	78653287	q14.1	959.293	Loss	-0.5478959
	chr5	160819324	167082649	q34	6263.326	Gain	0.39421514
	chr6	3051802	58722020	p25.2 - p11.1	55670.219	Gain	0.25859472
	chr6	62040890	170707926	q11.1 - q27	108667.037	Gain	0.2996677
	chr7	187615	57327440	p22.3 - p11.2	57139.826	Gain	0.25963998
	chr7	62153588	158781397	q11.21 - q36.3	96627.81	Gain	0.28916752
	chr8	13324774	15886768	p22	2561.995	Gain	0.4906595
	chr8	60180511	146250824	q12.1 - q24.3	86070.314	Gain	0.6277021
	chr11	21123640	29151796	p15.1 - p14.1	8028.157	Gain	0.30883527
	chr11	36930499	44562851	p12 - p11.2	7632.353	Gain	0.33736706
	chr13	38990710	51334158	q13.3 - q14.3	12343.449	Loss	-0.35025382
	chr15	31093704	43813736	q13.3 - q21.1	12720.033	Loss	-0.39908335
	chr16	94314	34903000	p13.3 - p11.1	34808.687	Loss	-0.33747518
	chr16	45122058	88638764	q11.2 - q24.3	43516.707	Loss	-0.35120928
	chr19	278273	24132581	p13.3 - p12	23854.309	Gain	0.32867017
	chr19	32964337	63775385	q12 - q13.43	30811.049	Gain	0.30372742
	chr20	1211324	26023841	p13 - p11.1	24812.518	Gain	0.30649188
	chr20	29536104	62363633	q11.21 - q13.33	32827.53	Gain	0.2744364
	chr21	14964772	46843337	q11.2 - q22.3	31878.566	Gain	0.34282717
	chrX	218292	58068490	p22.33 - p11.1	57850.199	Gain	0.2887987
	chrX	31803265	35917559	p21.1	4114.295	Gain	0.7020278
	chrX	62181725	153581117	q11.1 - q28	91399.393	Gain	0.38558775
	chrX	73802172	94867105	q13.3 - q21.33	21064.934	Gain	0.58146393
	chrX	132594493	132765458	q26.2	170.966	Deletion	-1.3147769
014	chr1	144127702	247139492	q21.1 - q44	103011.791	Gain	0.4470619
	chr4	74566335	190706331	q13.3 - q35.2	116139.997	Loss	-0.38090056
	chr6	101843782	103286627	q16.3	1442.846	Gain	0.5971515
	chr6	154533216	170732033	q25.2 - q27	16198.818	Loss	-0.4495872
	chr7	290112	55150487	p22.3 - p11.2	54860.376	Gain	0.44670844
	chr7	69801505	127199897	q11.22 - q32.1	57398.393	Loss	-0.38718566
	chr8	13324774	17111850	p22	3787.077	Gain	0.41229236
	chr8	105857203	109011867	q22.3 - q23.1	3154.665	Gain	0.38391262
	chr9	103202198	106333332	q31.1	3131.135	Gain	0.4257807
	chr10	4710076	5452287	p15.1	742.212	Gain	0.39287183
	chr10	42209250	135222482	q11.21 - q26.3	93013.233	Loss	-0.42367476
	chr15	52344822	100282878	q21.3 - q26.3	47938.057	Loss	-0.46036148
	chr16	5856888	8440445	p13.3 - p13.2	2583.558	Gain	0.3260614
	chr16	11549970	12664828	p13.13 - p13.12	1114.859	Loss	-0.5549137
	chr16	63876662	88638764	q21 - q24.3	24762.103	Loss	-0.4767086
	chr17	87009	13953779	p13.3 - p12	13866.771	Loss	-0.44487816
	chr17	14189874	22002704	p12 - p11.2	7812.831	Gain	0.3259026
	chr17	56915827	61723954	q23.2 - q24.2	4808.128	Gain	0.35847646
	chr17	61765590	78623230	q24.2 - q25.3	16857.641	Loss	-0.48966327
	chr18	353234	14918854	p11.32 - p11.21	14565.621	Gain	0.2599326
	chr18	21276655	76083117	q11.2 - q23	54806.463	Loss	-0.39064258

	chr19	8572581	12601172	p13.2 - p13.13	4028.592	Loss	-0.48083007
	chr19	53810369	63784382	q13.33 - q13.43	9974.014	Gain	0.38592944
	chrX	1091	2662039	p22.33	2660.949	Loss	-0.29684263
	chrX	31996853	34048503	p21.1	2051.651	Gain	0.48854676
	chrX	83545314	83641517	q21.1	96.204	Gain	1.1759791
	chrY	1091	2662039	p11.32 - p11.31	2660.949	Loss	-0.29684263
	chrY	2716461	10511314	p11.31 - p11.2	7794.854	Deletion	-1.1792762
	chrY	12593244	27176992	q11.21 - q11.23	14583.749	Loss	-0.97379464
	chrY	17284587	19613917	q11.221 - q11.222	2329.331	Loss	-0.43854457
015	chr5	1852098	16207654	p15.33 - p15.1	14355.557	Gain	0.27997312
	chr5	160819324	167082649	q34	6263.326	Gain	0.33964047
	chr8	48067862	145120207	q11.1 - q24.3	97052.346	Gain	0.25210354
	chr11	128761187	132514983	q24.3 - q25	3753.797	Gain	0.31989744
	chr19	58358846	63636460	q13.41 - q13.43	5277.615	Gain	0.2656307
016	chr1	144127702	247139492	q21.1 - q44	103011.791	Gain	0.2737407
	chr3	77137293	87377686	p12.3 - p11.2	10240.394	Gain	0.29157394
	chr3	126787794	196542278	q21.2 - q29	69754.485	Gain	0.275306
	chr8	13295522	17821053	p22	4525.532	Gain	0.25517485
	chr8	52414812	146024209	q11.22 - q24.3	93609.398	Gain	0.3137659
	chr9	2898782	8207827	p24.2 - p24.1	5309.046	Loss	-0.41906944
	chr11	46522252	47962213	p11.2	1439.962	Loss	-0.44686162
	chr12	100682	34236852	p13.33 - p11.1	34136.171	Loss	-0.2694962
	chr13	52125944	113532325	q14.3 - q34	61406.382	Gain	0.45677176
	chr14	19323579	105399038	q11.2 - q32.33	86075.46	Loss	-0.27574024
	chr16	94314	30027260	p13.3 - p11.2	29932.947	Loss	-0.30886298
	chr17	87009	17592700	p13.3 - p11.2	17505.692	Loss	-0.2815487
	chr19	278273	17268252	p13.3 - p13.11	16989.98	Loss	-0.3190097
	chr19	33198460	63775385	q12 - q13.43	30576.926	Gain	0.2526996
	chr20	1109200	23925553	p13 - p11.21	22816.354	Gain	0.31347865
	chr22	16023930	49291101	q11.1 - q13.33	33267.172	Loss	-0.32276687
	chrX	31803265	35917559	p21.1	4114.295	Gain	0.29275754
	chrY	2716461	10550478	p11.31 - p11.2	7834.018	Loss	-0.6440996
	chrY	12593244	57586758	q11.21 - q12	44993.515	Loss	-0.48662603
017	chr4	67133322	189902947	q13.2 - q35.2	122769.626	Loss	-0.44984668
	chr5	53260681	106489497	q11.2 - q21.3	53228.817	Loss	-0.5002432
	chr6	352231	58506751	p25.3 - p11.1	58154.521	Gain	0.7020909
	chr8	101986914	146250824	q22.3 - q24.3	44263.911	Gain	0.5854837
	chr9	856525	39146954	p24.3 - p13.1	38290.43	Loss	-0.4345447
	chr9	70327267	138656314	q13 - q34.3	68329.048	Loss	-0.4347382
	chr10	183492	37285570	p15.3 - p11.21	37102.079	Loss	-0.3023594
	chr10	42209250	135084833	q11.21 - q26.3	92875.584	Loss	-0.2871747
	chr11	128277435	133687965	q24.3 - q25	5410.531	Gain	0.42634076
	chr12	85423703	132278059	q21.32 - q24.33	46854.357	Loss	-0.3590917
	chr14	49164493	105399038	q22.1 - q32.33	56234.546	Loss	-0.5030441
	chr16	45122058	88601624	q11.2 - q24.3	43479.567	Loss	-0.40092403
	chr17	87009	21857788	p13.3 - p11.2	21770.78	Loss	-0.39770022
	chr18	4696135	13428287	p11.31 - p11.21	8732.153	Gain	0.2583726
	chr21	13783127	27905665	q11.2 - q21.3	14122.539	Loss	-0.7306073
	chr22	16023930	49291101	q11.1 - q13.33	33267.172	Loss	-0.44387966
	chrX	2661980	56860755	p22.33 - p11.1	54198.776	Loss	-0.35910445
	chrX	61848414	154727558	q11.1 - q28	92879.145	Loss	-0.27748454
	chrY	2783745	10622121	p11.31 - p11.2	7838.377	Loss	-0.5219759

	chrY	19951287	57586758	q11.222 - q12	37635.472	Loss	-0.43632197
018	chr1	79211223	83160805	p31.1	3949.583	Gain	0.4314021
	chr1	143639135	247139492	q21.1 - q44	103500.358	Gain	0.50583535
	chr2	32444	28780918	p25.3 - p23.2	28748.475	Gain	0.68180245
	chr3	73168400	90176395	p13 - p11.1	17007.996	Gain	0.25649133
	chr3	162217145	178221283	q26.1 - q26.32	16004.139	Gain	0.32616872
	chr4	7335000	48350883	p16.1 - p12	41015.884	Gain	0.26675084
	chr4	58720843	67029204	q12 - q13.2	8308.362	Gain	0.5355459
	chr5	924638	45865412	p15.33 - p11	44940.775	Gain	0.28154656
	chr5	55499495	70784638	q11.2 - q13.2	15285.144	Loss	-0.56973684
	chr7	290112	36891004	p22.3 - p14.2	36600.893	Gain	0.36267218
	chr8	211611	43452795	p23.3 - p11.1	43241.185	Loss	-0.51386964
	chr9	91624139	134933018	q22.2 - q34.2	43308.88	Loss	-0.25425822
	chr10	42209250	78923854	q11.21 - q22.3	36714.605	Gain	0.26748702
	chr10	124106670	135222482	q26.13 - q26.3	11115.813	Loss	-0.49679762
	chr13	44762385	94741905	q14.12 - q32.1	49979.521	Loss	-0.4167477
	chr16	49024407	66023997	q12.1 - q22.1	16999.591	Gain	0.2594245
	chr16	70647078	88638764	q22.3 - q24.3	17991.687	Loss	-0.4881369
	chr17	87009	21857788	p13.3 - p11.2	21770.78	Loss	-0.251539
	chr17	41236006	78623230	q21.31 - q25.3	37387.225	Gain	0.37553617
	chr20	29761480	62323759	q11.21 - q13.33	32562.28	Gain	0.3278635
	chrX	19972474	58068490	p22.12 - p11.1	38096.017	Gain	0.41871995
	chrX	62018861	153943360	q11.1 - q28	91924.5	Gain	0.48449433
019	chr1	3462768	66844899	p36.32 - p31.3	63382.132	Loss	-0.4766521
	chr1	67923755	69145061	p31.3 - p31.2	1221.307	Gain	0.7751427
	chr2	76563906	82895654	p12	6331.749	Gain	0.32573995
	chr3	77222495	90102363	p12.3 - p11.1	12879.869	Gain	0.33188406
	chr3	162217145	170347251	q26.1 - q26.2	8130.107	Gain	0.29720345
	chr5	924638	46136124	p15.33 - p11	45211.487	Gain	0.51581573
	chr5	49725929	179964347	q11.1 - q35.3	130238.419	Gain	0.43133405
	chr6	63348944	170732033	q11.1 - q27	107383.09	Loss	-0.4524885
	chr7	187615	57327440	p22.3 - p11.2	57139.826	Gain	0.477827
	chr7	62291739	158781397	q11.21 - q36.3	96489.659	Gain	0.4762964
	chr9	7192876	18794207	p24.1 - p22.1	11601.332	Gain	0.25776893
	chr9	20607752	22755281	p21.3	2147.53	Loss	-0.5435122
	chr9	24022718	32195829	p21.3 - p21.1	8173.112	Gain	0.29959083
	chr9	135344331	138814989	q34.2 - q34.3	3470.659	Gain	0.3604564
	chr10	54710584	61134282	q21.1	6423.699	Gain	0.34751257
	chr10	134277058	135254513	q26.3	977.456	Gain	0.51186806
	chr11	303837	2996477	p15.5 - p15.4	2692.641	Gain	0.33358118
	chr11	22356015	48250331	p14.3 - p11.2	25894.317	Loss	-0.461585
	chr11	66780416	69727064	q13.1 - q13.3	2946.649	Gain	0.33596298
	chr12	86651989	132278059	q21.32 - q24.33	45626.071	Loss	-0.55082387
	chr16	2434221	3081611	p13.3	647.391	Gain	0.5193644
	chr16	4104241	7904339	p13.3 - p13.2	3800.099	Gain	0.31119904
	chr16	57785182	64078112	q21	6292.931	Gain	0.40467146
	chr17	1613876	21139998	p13.3 - p11.2	19526.123	Loss	-0.43284494
	chr17	24253086	37915463	q11.2 - q21.31	13662.378	Loss	-0.4919656
	chr17	74363212	78623230	q25.3	4260.019	Gain	0.3811527
	chr21	14667810	46892352	q11.2 - q22.3	32224.543	Loss	-0.3445901
	chr22	44614141	49525130	q13.31 - q13.33	4910.99	Gain	0.33779833
	chrX	2719027	58068490	p22.33 - p11.1	55349.464	Gain	0.9091469

	chrX	61848414	154407365	q11.1 - q28	92558.952	Gain	0.9517493
	chrX	78089914	96742926	q21.1 - q21.33	18653.013	Gain	1.1540766
	chrY	2716461	10511314	p11.31 - p11.2	7794.854	Deletion	-3.108997
	chrY	12571053	27176992	q11.21 - q11.23	14605.94	Deletion	-2.0888968
	chrY	14214157	14487081	q11.21 - q11.221	272.925	Deletion	-3.3053012
	chrY	17098148	19613917	q11.221 - q11.222	2515.77	Deletion	-1.072247
020	chr1	7503586	9565437	p36.23 - p36.22	2061.852	Gain	0.37478516
	chr1	143639135	156420261	q21.1 - q23.1	12781.127	Gain	0.90505856
	chr1	183392597	199127329	q25.3 - q32.1	15734.733	Gain	0.2759712
	chr1	239244684	247179291	q43 - q44	7934.608	Gain	0.92860746
	chr3	68949	74963524	p26.3 - p12.3	74894.576	Loss	-0.36527333
	chr3	76537524	78688333	p12.3	2150.81	plification	2.2025223
	chr3	95063426	115116766	q11.2 - q13.31	20053.341	Gain	1.3843954
	chr3	115538038	198880094	q13.31 - q29	83342.057	Gain	0.40817237
	chr4	61552	48758058	p16.3 - p11	48696.507	Loss	-0.29746947
	chr4	52383858	190706331	q11 - q35.2	138322.474	Loss	-0.306561
	chr5	5396166	10011299	p15.32 - p15.2	4615.134	Loss	-0.3120512
	chr5	10120817	46136124	p15.2 - p11	36015.308	Gain	0.52449375
	chr5	49725929	58370263	q11.1 - q11.2	8644.335	Gain	0.92379814
	chr5	65155918	70931296	q12.3 - q13.2	5775.379	Gain	0.33323443
	chr5	85235027	180617107	q14.3 - q35.3	95382.081	Loss	-0.3213778
	chr6	455657	58722020	p25.3 - p11.1	58266.364	Gain	0.9956514
	chr6	62040890	67086471	q11.1 - q12	5045.582	Gain	1.1611632
	chr6	96345486	170707926	q16.1 - q27	74362.441	Loss	-0.30451295
	chr7	105429248	158781397	q22.2 - q36.3	53352.15	Gain	0.34954837
	chr8	514066	33525017	p23.3 - p12	33010.952	Loss	-0.35074615
	chr8	48386362	146250824	q11.21 - q24.3	97864.463	Gain	0.39099586
	chr9	261257	39146954	p24.3 - p13.1	38885.698	Loss	-0.29882026
	chr9	70327267	139602359	q13 - q34.3	69275.093	Loss	-0.32444802
	chr10	138206	38395999	p15.3 - p11.21	38257.794	Gain	0.30326
	chr10	42209250	63492370	q11.21 - q21.2	21283.121	Gain	0.26583344
	chr11	3030205	48250331	p15.4 - p11.2	45220.127	Loss	-0.35342717
	chr12	1183521	34236852	p13.33 - p11.1	33053.332	Gain	0.33321276
	chr12	36858944	131856521	q12 - q24.33	94997.578	Gain	0.3029399
	chr13	45949119	90295974	q14.13 - q31.3	44346.856	Loss	-0.8479774
	chr13	90423948	104320727	q31.3 - q33.2	13896.78	Gain	0.36723045
	chr13	104449575	113532325	q33.2 - q34	9082.751	plification	2.155419
	chr14	63040068	105013921	q23.2 - q32.33	41973.854	Loss	-0.39085743
	chr15	18835660	31697407	q11.2 - q14	12861.748	Loss	-0.35387084
	chr15	31893225	46051831	q14 - q21.1	14158.607	Gain	0.29951215
	chr16	21585396	34903000	p12.2 - p11.1	13317.605	Loss	-0.42783633
	chr16	45122058	88621982	q11.2 - q24.3	43499.925	Loss	-0.4266816
	chr17	1613876	21139998	p13.3 - p11.2	19526.123	Loss	-0.33700785
	chr17	22614479	78623230	q11.1 - q25.3	56008.752	Gain	0.30226508
	chr18	4316	13815806	p11.32 - p11.21	13811.491	Loss	-0.40848798
	chr18	18968555	76018409	q11.2 - q23	57049.855	Loss	-0.33624473
	chr19	41966756	49764793	q13.12 - q13.31	7798.038	Loss	-0.35452402
	chr19	49842066	63407936	q13.31 - q13.43	13565.871	Gain	0.2889156
	chr20	118826	26023841	p13 - p11.1	25905.016	Gain	0.29924545
	chr20	29501535	62343283	q11.21 - q13.33	32841.749	Gain	0.30309507
	chr21	13562263	15028897	q11.2	1466.635	Gain	1.3620015
	chr21	34999700	35696672	q22.12	696.973	Gain	0.592946

	chr22	41536788	48956066	q13.2 - q13.33	7419.279	plification	2.043341
	chrX	2754701	58068490	p22.33 - p11.1	55313.79	Gain	0.7832607
	chrX	61848414	154561665	q11.1 - q28	92713.252	Gain	0.8078245
	chrY	2666344	10511314	p11.31 - p11.2	7844.971	Loss	-0.36770168
021	chr1	880808	52088655	p36.33 - p32.3	51207.848	Loss	-0.595702
	chr1	52157850	120885791	p32.3 - p11.2	68727.942	Gain	0.4551539
	chr1	143700072	247118482	q21.1 - q44	103418.411	Gain	0.47886005
	chr4	249110	44418976	p16.3 - p13	44169.867	Gain	0.5020012
	chr4	133037099	190706331	q28.3 - q35.2	57669.233	Loss	-0.6813529
	chr7	62291739	158781397	q11.21 - q36.3	96489.659	Gain	0.49993283
	chr8	84934664	146201771	q21.2 - q24.3	61267.108	Gain	0.491171
	chr10	46431590	134108936	q11.22 - q26.3	87677.347	Loss	-0.6367877
	chr12	109418170	132278059	q24.11 - q24.33	22859.89	Loss	-0.6549645
	chr13	20886939	113373792	q12.11 - q34	92486.854	Loss	-0.5607181
	chr16	46271	30811182	p13.3 - p11.2	30764.912	Loss	-0.6430677
	chr16	69831902	88621982	q22.3 - q24.3	18790.081	Loss	-0.63429344
	chr17	1613876	11407931	p13.3 - p12	9794.056	Loss	-0.64495564
	chr18	32632356	76018409	q12.2 - q23	43386.054	Loss	-0.68217224
	chr19	372537	13389878	p13.3 - p13.13	13017.342	Loss	-0.4823758
	chr21	13562263	46880878	q11.2 - q22.3	33318.616	Loss	-0.569571
	chrY	2661980	10511314	p11.31 - p11.2	7849.335	Deletion	-2.2652955
	chrY	12571053	57623977	q11.21 - q12	45052.925	Deletion	-1.534362
022	chr1	184816387	197385396	q31.1 - q31.3	12569.01	Gain	0.3530798
	chr2	76796517	82895654	p12	6099.138	Gain	0.27462038
	chr4	37705788	40680782	p14	2974.995	Loss	-0.30226594
	chr5	17406393	29407781	p15.1 - p13.3	12001.389	Gain	0.3077402
	chr6	90864523	103286627	q15 - q16.3	12422.105	Gain	0.26236528
	chr6	120506683	125021068	q22.31	4514.386	Gain	0.40008873
	chr8	69094772	146250824	q13.2 - q24.3	77156.053	Gain	0.37623507
	chr9	135344331	138814989	q34.2 - q34.3	3470.659	Gain	0.26023957
	chr10	138206	35279437	p15.3 - p11.21	35141.232	Gain	0.25576463
	chr10	53416626	61134282	q21.1	7717.657	Gain	0.29182675
	chr11	303837	2996477	p15.5 - p15.4	2692.641	Gain	0.31595927
	chr16	57785182	63592637	q21	5807.456	Gain	0.3238085
	chr19	49947017	50886589	q13.31 - q13.32	939.573	Gain	0.49772748
	chrX	31803265	36210647	p21.1	4407.383	Gain	0.30473322
023	chr1	144127702	247118482	q21.1 - q44	102990.781	Gain	0.37472078
	chr3	75047041	87287251	p12.3 - p11.2	12240.211	Gain	0.4086452
	chr5	204737	26103882	p15.33 - p14.1	25899.146	Gain	0.26180676
	chr6	630345	58722020	p25.3 - p11.1	58091.676	Gain	0.6282676
	chr7	524935	57498383	p22.3 - p11.1	56973.449	Gain	0.3975245
	chr7	62291739	158781397	q11.21 - q36.3	96489.659	Gain	0.4532301
	chr8	83320460	146201771	q21.13 - q24.3	62881.312	Gain	0.6713013
	chr9	130053946	140128736	q34.11 - q34.3	10074.791	Gain	0.29422802
	chr13	51224460	90124152	q14.3 - q31.3	38899.693	Gain	0.25058788
	chr16	8193757	31284827	p13.2 - p11.2	23091.071	Loss	-0.5070855
	chr16	45122058	87034467	q11.2 - q24.2	41912.41	Loss	-0.41939604
	chr17	1932367	21158567	p13.3 - p11.2	19226.201	Loss	-0.37220803
	chr17	25637986	78623230	q11.2 - q25.3	52985.245	Gain	0.4186947
	chr19	278273	24072630	p13.3 - p12	23794.358	Gain	0.33932072
	chr19	32964337	61800568	q12 - q13.43	28836.232	Gain	0.29099044
	chr22	44614141	49525130	q13.31 - q13.33	4910.99	Gain	0.5055965

	chrX	109064	2372192	p22.33	2263.129	Gain	0.95939267
	chrX	31372643	35590846	p21.2 - p21.1	4218.204	Gain	0.5545415
	chrY	109064	2372192	p11.32 - p11.31	2263.129	Gain	0.95939267
	chrY	12571053	57745301	q11.21 - q12	45174.249	Gain	0.44901612
024	chr1	143639135	247139492	q21.1 - q44	103500.358	Gain	0.45056966
	chr2	98228328	111342998	q11.2 - q13	13114.671	Gain	0.26017028
	chr3	73426923	90336752	p13 - p11.1	16909.83	Gain	0.39666238
	chr3	194961377	197289184	q29	2327.808	Loss	-0.39567825
	chr4	184255364	186536068	q35.1	2280.705	Loss	-0.39612624
	chr5	924638	29938767	p15.33 - p13.3	29014.13	Gain	0.7692647
	chr5	33215945	41464795	p13.3 - p13.1	8248.851	Gain	0.42968127
	chr5	94882171	110641173	q15 - q22.1	15759.003	Gain	0.25452456
	chr6	115426	58122491	p25.3 - p11.2	58007.066	Gain	0.35930628
	chr6	62040890	108595221	q11.1 - q21	46554.332	Gain	0.3320226
	chr6	120033969	128685062	q22.31 - q22.33	8651.094	Gain	0.25958836
	chr7	78619146	86412277	q21.11 - q21.12	7793.132	Gain	0.42265218
	chr8	38309927	42449179	p12 - p11.21	4139.253	Loss	-0.3039097
	chr8	48437368	146250824	q11.21 - q24.3	97813.457	Gain	0.8271013
	chr9	102943977	105897511	q31.1	2953.535	Gain	0.4527103
	chr11	274838	46490960	p15.5 - p11.2	46216.123	Gain	0.36302072
	chr11	68438799	69840565	q13.2 - q13.3	1401.767	Gain	0.90761703
	chr11	130451440	132678521	q25	2227.082	Gain	0.51074684
	chr12	47340334	49922248	q13.11 - q13.13	2581.915	Loss	-0.29298213
	chr16	29582349	29824895	p11.2	242.547	Gain	0.91761553
	chr19	1709857	2887626	p13.3	1177.77	Gain	0.38583535
	chrY	2716461	8300713	p11.31 - p11.2	5584.253	Loss	-0.583828
	chrY	12627138	27176992	q11.21 - q11.23	14549.855	Loss	-0.25402102
025	chr1	143815233	247139492	q21.1 - q44	103324.26	Gain	0.25394836
	chr3	127751292	199324736	q21.3 - q29	71573.445	Gain	0.420489
	chr5	31487385	33156304	p13.3	1668.92	Loss	-0.4677055
	chr6	26038737	26354877	p22.2 - p22.1	316.141	Gain	0.8416729
	chr8	33483345	41809596	p12 - p11.21	8326.252	Gain	0.4226041
	chr8	57048730	117347162	q12.1 - q23.3	60298.433	Gain	0.4475724
	chr10	138206	38468053	p15.3 - p11.21	38329.848	Gain	0.34959197
	chr10	122004660	135254513	q26.12 - q26.3	13249.854	Gain	0.27246332
	chr13	78826674	114077122	q31.1 - q34	35250.449	Gain	0.26711333
	chr14	19895020	29621157	q11.2 - q12	9726.138	Gain	0.31242067
	chr15	79380848	99528261	q25.1 - q26.3	20147.414	Gain	0.27663508
	chr17	11407872	22129948	p12 - p11.1	10722.077	Gain	0.25022933
	chrY	2716461	9208336	p11.31 - p11.2	6491.876	Loss	-0.6286124
026	chr1	143815233	247179291	q21.1 - q44	103364.059	Gain	0.5818218
	chr3	28315276	56305132	p24.1 - p14.3	27989.857	Gain	0.25159264
	chr3	77222495	90176395	p12.3 - p11.1	12953.901	Gain	0.28329486
	chr4	52719921	191004108	q12 - q35.2	138284.188	Loss	-0.25827608
	chr9	70327267	83895284	q13 - q21.32	13568.018	Loss	-0.5882487
	chr11	274838	44609795	p15.5 - p11.2	44334.958	Gain	0.43781137
	chr11	65891350	79702160	q13.1 - q14.1	13810.811	Gain	0.9774997
	chr11	80790766	134160011	q14.1 - q25	53369.246	Loss	-0.27522582
	chr12	100682	9697608	p13.33 - p13.31	9596.927	Loss	-0.29520667
	chr12	10053270	33646504	p13.2 - p11.1	23593.235	Gain	0.2845052
	chr12	65994571	74710465	q14.3 - q21.2	8715.895	Gain	0.642867
	chr12	79113501	132193660	q21.31 - q24.33	53080.16	Loss	-0.25626364

	chr15	18835660	41486825	q11.2 - q15.3	22651.166	Loss	-0.32979494
	chr16	682550	34847384	p13.3 - p11.1	34164.835	Loss	-0.27048498
	chr19	6086357	10485291	p13.3 - p13.2	4398.935	Gain	0.38987777
	chr19	15199118	24072630	p13.12 - p12	8873.513	Gain	0.2556778
	chrX	263301	7704191	p22.33 - p22.31	7440.891	Gain	0.32587466
	chrX	7780636	58068490	p22.31 - p11.1	50287.855	Gain	0.8955213
	chrX	7827300	26151941	p22.31 - p21.3	18324.642	Gain	0.55618596
	chrX	26322343	58068490	p21.3 - p11.1	31746.148	Gain	1.1014895
	chrX	61848414	154407365	q11.1 - q28	92558.952	Gain	0.97844017
	chrX	72701062	91391564	q13.2 - q21.31	18690.503	Gain	1.2143482
	chrX	99176526	135678352	q22.1 - q26.3	36501.827	Gain	0.8480889
	chrX	154494590	154886101	q28	391.512	Gain	0.7518979
	chrY	2716461	10511314	p11.31 - p11.2	7794.854	Deletion	-3.1483827
	chrY	12571053	27176992	q11.21 - q11.23	14605.94	Deletion	-2.197306
	chrY	57440809	57745301	q12	304.493	Gain	0.77078533
027	chr3	345339	90336752	p26.3 - p11.1	89991.414	Gain	0.43225732
	chr3	95063426	199028560	q11.2 - q29	103965.135	Gain	0.45630106
	chr4	6504029	8419080	p16.1	1915.052	Gain	0.4438096
	chr4	68288998	191133668	q13.2 - q35.2	122844.671	Loss	-0.31107575
	chr5	924638	45865412	p15.33 - p11	44940.775	Gain	0.29538962
	chr5	50094782	179811088	q11.1 - q35.3	129716.307	Gain	0.2529269
	chr6	1636776	57648080	p25.3 - p11.2	56011.305	Gain	0.28346238
	chr6	62040890	170433574	q11.1 - q27	108392.685	Gain	0.29504105
	chr7	187615	55842141	p22.3 - p11.2	55654.527	Gain	0.32803202
	chr7	78474418	158781397	q21.11 - q36.3	80306.98	Gain	0.4884704
	chr8	704325	40699315	p23.3 - p11.21	39994.991	Loss	-0.3681854
	chr8	47800500	146250824	q11.1 - q24.3	98450.325	Gain	0.3142433
	chr11	303837	51289318	p15.5 - p11.12	50985.482	Gain	0.4849721
	chr11	55379046	64115300	q11 - q13.1	8736.255	Gain	0.48940197
	chr11	66370535	73148715	q13.1 - q13.4	6778.181	Gain	1.2466118
	chr12	752975	34236852	p13.33 - p11.1	33483.878	Gain	0.50129175
	chr12	36973165	131791665	q12 - q24.33	94818.501	Gain	0.5088112
	chr17	1649795	20133761	p13.3 - p11.2	18483.967	Loss	-0.35062602
	chr17	22614479	78623230	q11.1 - q25.3	56008.752	Gain	0.26207608
	chr20	118826	26023841	p13 - p11.1	25905.016	Gain	0.26083428
	chr20	32611751	62343283	q11.22 - q13.33	29731.533	Gain	0.3612629
	chrY	94062	10622121	p11.32 - p11.2	10528.06	Loss	-0.30882314
	chrY	12627138	57682436	q11.21 - q12	45055.299	Loss	-0.6455953
028	chr1	143639135	247118482	q21.1 - q44	103479.348	Gain	0.7015473
	chr2	38246167	38940432	p22.2 - p22.1	694.266	Loss	-0.71435064
	chr4	118581927	190706331	q26 - q35.2	72124.405	Loss	-0.661464
	chr5	924638	29643288	p15.33 - p13.3	28718.651	Gain	0.93849444
	chr6	593433	31002218	p25.3 - p21.33	30408.786	Gain	0.41328877
	chr6	43101791	43134380	p21.1	32.59	Deletion	-1.4328362
	chr7	63087010	90535410	q11.21 - q21.13	27448.401	Loss	-0.69214547
	chr7	107001429	107577562	q22.3 - q31.1	576.134	Loss	-0.7162876
	chr9	319684	12038612	p24.3 - p23	11718.929	Gain	0.50340647
	chr10	138206	35140690	p15.3 - p11.21	35002.485	Gain	0.4551306
	chr10	54710584	62341322	q21.1 - q21.2	7630.739	Gain	0.29164237
	chr10	75828276	134446451	q22.2 - q26.3	58618.176	Loss	-0.69718456
	chr11	46522252	48345331	p11.2	1823.08	Loss	-0.33255345
	chr13	25391304	80808086	q12.13 - q31.1	55416.783	Loss	-0.6476524

	chr13	100025138	114077122	q32.3 - q34	14051.985	Gain	0.577538
	chr16	45122058	88621982	q11.2 - q24.3	43499.925	Loss	-0.6934314
	chr17	210970	16186663	p13.3 - p11.2	15975.694	Loss	-0.7226077
	chr17	35330867	78623230	q12 - q25.3	43292.364	Gain	0.40078688
	chr18	23010698	30598634	q11.2 - q12.1	7587.937	Gain	0.27629665
	chr18	45423715	76018409	q21.1 - q23	30594.695	Loss	-0.686149
	chr19	278273	11167149	p13.3 - p13.2	10888.877	Loss	-0.6237792
	chrX	128767274	129134737	q25	367.464	Loss	-0.7569636
	chrY	307301	7751489	p11.32 - p11.2	7444.189	Loss	-0.28128204
029	chr1	143815233	246695582	q21.1 - q44	102880.35	Gain	0.32886752
	chr3	68949	77180067	p26.3 - p12.3	77111.119	Loss	-0.40744737
	chr4	305565	48182792	p16.3 - p12	47877.228	Gain	0.41437986
	chr4	53755607	191133668	q12 - q35.2	137378.062	Loss	-0.34009627
	chr5	924638	30184958	p15.33 - p13.3	29260.321	Gain	0.2689847
	chr5	53626875	180617107	q11.2 - q35.3	126990.233	Loss	-0.3850949
	chr7	78549068	86412277	q21.11 - q21.12	7863.21	Gain	0.2947508
	chr8	1561083	43452795	p23.3 - p11.1	41891.713	Loss	-0.3618119
	chr9	261257	39146954	p24.3 - p13.1	38885.698	Loss	-0.31664774
	chr9	70327267	139602359	q13 - q34.3	69275.093	Loss	-0.3989728
	chr10	54710584	62218019	q21.1 - q21.2	7507.436	Gain	0.30596513
	chr12	222982	30700931	p13.33 - p11.21	30477.95	Loss	-0.39904496
	chr12	115609026	116656179	q24.22	1047.154	Gain	0.3942308
	chr16	45122058	88638764	q11.2 - q24.3	43516.707	Loss	-0.46984038
	chr17	1391616	21139998	p13.3 - p11.2	19748.383	Loss	-0.44188046
	chr18	22909600	30598634	q11.2 - q12.1	7689.035	Gain	0.25873947
	chr19	372537	23164126	p13.3 - p12	22791.59	Loss	-0.38472477
030	chr1	183373195	197385396	q25.3 - q31.3	14012.202	Gain	0.46197152
	chr2	99953826	119996047	q11.2 - q14.2	20042.222	Gain	0.27780497
	chr5	924638	26103882	p15.33 - p14.1	25179.245	Gain	0.2693018
	chr7	65130855	78392753	q11.21 - q21.11	13261.899	Loss	-0.26104206
	chr7	78474418	86480289	q21.11 - q21.12	8005.872	Gain	0.35772344
	chr11	107683813	127394467	q22.3 - q24.2	19710.655	Loss	-0.2879418
	chr12	47024632	50151519	q13.11 - q13.13	3126.888	Loss	-0.3731654
	chr15	38289172	43671709	q15.1 - q21.1	5382.538	Loss	-0.2894487
	chr16	7178607	31284827	p13.2 - p11.2	24106.221	Loss	-0.2548013
	chr16	45122058	88621982	q11.2 - q24.3	43499.925	Loss	-0.2573641
031	chr1	49732367	67984539	p33 - p31.3	18252.173	Loss	-0.46340883
	chr1	184908007	245804497	q31.1 - q44	60896.491	Gain	0.38278478
	chr5	88229048	180617107	q14.3 - q35.3	92388.06	Loss	-0.40162134
	chr6	630345	56474156	p25.3 - p12.1	55843.812	Gain	0.35809916
	chr6	62040890	170700061	q11.1 - q27	108659.172	Loss	-0.35931253
	chr8	704325	43452795	p23.3 - p11.1	42748.471	Loss	-0.39649528
	chr8	48437368	146144837	q11.21 - q24.3	97707.47	Gain	0.40356076
	chr11	208365	50096841	p15.5 - p11.12	49888.477	Loss	-0.41544595
	chr11	55131703	64456881	q11 - q13.1	9325.179	Loss	-0.43450993
	chr12	7515180	34236852	p13.31 - p11.1	26721.673	Loss	-0.36182138
	chr12	103602885	132278059	q23.3 - q24.33	28675.175	Loss	-0.4957971
	chr13	18679724	112809469	q12.11 - q34	94129.746	Loss	-0.39072174
	chr16	46271	34402265	p13.3 - p11.1	34355.995	Loss	-0.4581829
	chr16	45122058	88621982	q11.2 - q24.3	43499.925	Loss	-0.42434487
	chr17	1602400	17910159	p13.3 - p11.2	16307.76	Loss	-0.40605822
	chr17	41212860	78623230	q21.31 - q25.3	37410.371	Gain	0.31475997

	chr18	21020515	66627931	q11.2 - q22.2	45607.417	Loss	-0.41539127
	chr19	1856897	17739860	p13.3 - p13.11	15882.964	Loss	-0.32565463
	chr19	18749734	19699544	p13.11	949.811	Gain	0.88248354
	chr21	13879604	46591026	q11.2 - q22.3	32711.423	Loss	-0.35475913
032	chr1	143815233	247118482	q21.1 - q44	103303.25	Gain	0.68732554
	chr2	302011	8927493	p25.3 - p25.1	8625.483	Gain	0.37650427
	chr3	75129926	87406085	p12.3 - p11.2	12276.16	Gain	0.284281
	chr3	173541520	196725250	q26.31 - q29	23183.731	Loss	-0.41781822
	chr4	52406688	191133668	q12 - q35.2	138726.981	Loss	-0.43953094
	chr5	204737	26103882	p15.33 - p14.1	25899.146	Gain	0.27058604
	chr5	137909738	180517872	q31.2 - q35.3	42608.135	Gain	0.38950744
	chr6	88141869	170700061	q15 - q27	82558.193	Loss	-0.3863904
	chr7	216122	57498383	p22.3 - p11.1	57282.262	Loss	-0.40115023
	chr7	62471018	158781397	q11.21 - q36.3	96310.38	Gain	0.43103725
	chr8	704325	43452795	p23.3 - p11.1	42748.471	Loss	-0.4100081
	chr8	47800500	146250824	q11.1 - q24.3	98450.325	Gain	0.7113496
	chr9	363868	38195685	p24.3 - p13.1	37831.818	Loss	-0.4394756
	chr12	3601474	34012013	p13.32 - p11.1	30410.54	Loss	-0.45511854
	chr13	18601703	113373792	q12.11 - q34	94772.09	Loss	-0.40451252
	chr14	19323579	105013921	q11.2 - q32.33	85690.343	Loss	-0.47358412
	chr16	1824115	20970038	p13.3 - p12.2	19145.924	Gain	0.30500546
	chr16	45122058	88621982	q11.2 - q24.3	43499.925	Loss	-0.44682726
	chr17	1649795	20133761	p13.3 - p11.2	18483.967	Loss	-0.443707
	chr19	48590762	48827422	q13.31	236.661	Gain	1.1139423
	chr22	15979429	27521073	q11.1 - q12.1	11541.645	Loss	-0.47227794
033	chr1	184816387	197385396	q31.1 - q31.3	12569.01	Gain	0.8447497
	chr3	68949	74963524	p26.3 - p12.3	74894.576	Loss	-0.27449664
	chr3	99172962	199251188	q11.2 - q29	100078.227	Loss	-0.26163673
	chr4	305565	48557847	p16.3 - p12	48252.283	Gain	0.5354255
	chr4	52879589	191133668	q12 - q35.2	138254.08	Loss	-0.61193085
	chr7	62471018	158781397	q11.21 - q36.3	96310.38	Gain	0.5218462
	chr9	319684	39146954	p24.3 - p13.1	38827.271	Loss	-0.6059084
	chr11	116543877	119137144	q23.3	2593.268	Loss	-0.34102207
	chr12	120273065	124484405	q24.31	4211.341	Loss	-0.3133731
	chr14	19323579	105013921	q11.2 - q32.33	85690.343	Loss	-0.27043435
	chr17	210970	14189933	p13.3 - p12	13978.964	Loss	-0.64193743
	chr17	15438550	17079954	p12 - p11.2	1641.405	Gain	0.91696477
	chr20	118826	26023841	p13 - p11.1	25905.016	Gain	0.43679067
	chr20	31085455	62343283	q11.21 - q13.33	31257.829	Gain	0.32105246
034	chr1	5855095	119293069	p36.31 - p12	113437.975	Loss	-0.46664783
	chr2	302011	8610188	p25.3 - p25.1	8308.178	Gain	0.45056522
	chr3	41152107	41292441	p22.1	140.335	plification	2.6930635
	chr3	42610827	43349239	p22.1	738.413	plification	2.9941418
	chr7	62153588	77792656	q11.21 - q21.11	15639.069	Loss	-0.35651475
	chr7	155023198	158781397	q36.3	3758.2	Gain	0.7817167
	chr8	21826992	43353273	p21.3 - p11.1	21526.282	Loss	-0.32431936
	chr8	56043163	146201771	q12.1 - q24.3	90158.609	Gain	0.96576893
	chr10	238464	38395999	p15.3 - p11.21	38157.536	Gain	0.29355118
	chr12	100682	32827592	p13.33 - p11.21	32726.911	Loss	-0.78108025
	chr12	36931877	132278059	q12 - q24.33	95346.183	Loss	-0.5083432
	chr13	18679724	21011477	q12.11	2331.754	Loss	-0.6413762
	chr14	19323579	105013921	q11.2 - q32.33	85690.343	Loss	-0.50322217

	chr15	28830208	97997129	q13.2 - q26.3	69166.922	Loss	-0.29046655
	chr16	3105059	34242154	p13.3 - p11.2	31137.096	Loss	-0.33499634
	chr16	45122058	88621982	q11.2 - q24.3	43499.925	Loss	-0.25384668
	chr17	210970	22129948	p13.3 - p11.1	21918.979	Loss	-0.7108403
	chr18	38618066	76018409	q12.3 - q23	37400.344	Loss	-0.7346115
	chr19	372537	23164126	p13.3 - p12	22791.59	Loss	-0.52995425
	chr19	37836946	63672832	q13.11 - q13.43	25835.887	Loss	-0.26478454
	chr20	69521	25454944	p13 - p11.21	25385.424	Loss	-0.4284084
	chr20	29352138	52638549	q11.21 - q13.2	23286.412	Loss	-0.5544276
	chr20	57520313	62343283	q13.32 - q13.33	4822.971	Gain	0.48662192
	chr21	13783127	46892352	q11.2 - q22.3	33109.226	Loss	-0.37834758
	chr22	15979429	46244603	q11.1 - q13.31	30265.175	Loss	-0.48769456
035	chr1	205007611	247139492	q32.1 - q44	42131.882	Loss	-0.4906035
	chr2	96527147	242656032	q11.2 - q37.3	146128.886	Loss	-0.45712045
	chr4	71309854	190706331	q13.3 - q35.2	119396.478	Loss	-0.44095635
	chr5	260981	5343154	p15.33 - p15.32	5082.174	Gain	0.6328987
	chr5	170004694	177590592	q35.1 - q35.3	7585.899	Loss	-0.39646715
	chr8	14775825	38959671	p22 - p11.23	24183.847	Loss	-0.50454944
	chr8	122932538	146201771	q24.13 - q24.3	23269.234	Loss	-0.5545677
	chr9	319684	30237453	p24.3 - p21.1	29917.77	Loss	-0.5672699
	chr9	70327267	131439018	q13 - q34.11	61111.752	Loss	-0.2698285
	chr10	46431590	125022153	q11.22 - q26.13	78590.564	Loss	-0.3823543
	chr11	1707889	43469098	p15.5 - p11.2	41761.21	Loss	-0.5464658
	chr11	57054076	133951370	q12.1 - q25	76897.295	Loss	-0.3213411
	chr12	222982	34012013	p13.33 - p11.1	33789.032	Loss	-0.53324485
	chr13	46351122	61054344	q14.2 - q21.31	14703.223	Loss	-0.4810429
	chr13	102073323	112517932	q33.1 - q34	10444.61	Gain	0.5056302
	chr14	49260318	105080399	q22.1 - q32.33	55820.082	Loss	-0.55838484
	chr16	47761263	88601624	q12.1 - q24.3	40840.362	Loss	-0.47691762
	chr17	5067461	16450802	p13.2 - p11.2	11383.342	Loss	-0.41425902
	chr18	27475714	75164621	q12.1 - q23	47688.908	Loss	-0.26685938
	chr19	372537	15144173	p13.3 - p13.12	14771.637	Loss	-0.36140928
	chr19	61809452	63784382	q13.43	1974.931	Loss	-0.56172055
	chr20	505786	25235554	p13 - p11.21	24729.769	Loss	-0.5914029
	chr22	16597351	49524226	q11.21 - q13.33	32926.876	Loss	-0.45960477
036	chr1	212021116	218237315	q32.3 - q41	6216.2	Gain	0.29724178
	chr1	235271668	239506603	q43	4234.936	Gain	0.27861804
	chr2	1102915	2667650	p25.3	1564.736	Gain	0.42736286
	chr3	197282504	198154829	q29	872.326	Loss	-0.43475118
	chr11	18030377	18843060	p15.1	812.684	Loss	-0.25469306
	chr11	129884810	133432246	q24.3 - q25	3547.437	Gain	0.27920815
	chr15	22821763	27994765	q11.2 - q13.1	5173.003	Gain	0.37617773
	chr16	75326840	79633907	q23.1 - q23.2	4307.068	Gain	0.25286356
	chr18	5045927	9029431	p11.31 - p11.22	3983.505	Gain	0.26788437
	chrX	31996853	35917559	p21.1	3920.707	Gain	0.32375455
	chrX	128705550	129184287	q25	478.738	Loss	-0.4534443
037	chr1	749625	116743105	p36.33 - p13.1	115993.481	Loss	-0.3106957
	chr1	116752525	120982693	p13.1 - p11.2	4230.169	Gain	0.32824162
	chr1	143639135	247179291	q21.1 - q44	103540.157	Gain	0.58853924
	chr4	61552	41313394	p16.3 - p13	41251.843	Loss	-0.49582008
	chr4	52383858	124038304	q11 - q28.1	71654.447	Loss	-0.26129636
	chr4	128809713	137337871	q28.1 - q28.3	8528.159	Gain	0.49735498

	chr4	182690730	190706331	q35.1 - q35.2	8015.602	Loss	-0.6038287
	chr5	452535	46136124	p15.33 - p11	45683.59	Gain	0.405016
	chr5	49725929	180517872	q11.1 - q35.3	130791.944	Gain	0.32464933
	chr7	78474418	95497715	q21.11 - q21.3	17023.298	Gain	0.29739317
	chr7	105876140	158343829	q22.2 - q36.3	52467.69	Loss	-0.5556933
	chr8	617655	38087555	p23.3 - p12	37469.901	Loss	-0.84123325
	chr8	38180851	39341583	p12 - p11.23	1160.733	Loss	-0.37167132
	chr8	39586203	43353273	p11.22 - p11.1	3767.071	Loss	-0.25846612
	chr8	47800500	146250824	q11.1 - q24.3	98450.325	Gain	0.38016352
	chr9	93019397	101152078	q22.31 - q22.33	8132.682	Loss	-0.90731466
	chr10	71002959	135254513	q21.3 - q26.3	64251.555	Gain	0.34171662
	chr14	19651899	105080399	q11.2 - q32.33	85428.501	Loss	-0.31676042
	chr16	5418323	8108270	p13.3 - p13.2	2689.948	Gain	0.30907917
	chr16	30315213	34847384	p11.2 - p11.1	4532.172	Loss	-0.62294644
	chr16	45172598	88638764	q11.2 - q24.3	43466.167	Loss	-0.60302913
	chr20	118826	26135794	p13 - p11.1	26016.969	Gain	0.48743656
	chr20	29352138	62343283	q11.21 - q13.33	32991.146	Gain	0.39900056
	chr21	13562263	46892352	q11.2 - q22.3	33330.09	Loss	-0.49131793
038	chr1	50845730	55676921	p33 - p32.3	4831.192	Loss	-0.2713768
	chr1	55857393	107584833	p32.3 - p13.3	51727.441	Gain	0.27797806
	chr1	143815233	247179291	q21.1 - q44	103364.059	Gain	0.6124065
	chr2	1141154	88833920	p25.3 - p11.2	87692.767	Loss	-0.27164987
	chr2	94892766	144471295	q11.1 - q22.2	49578.53	Gain	0.27598974
	chr3	100051041	135170575	q12.1 - q22.1	35119.535	Loss	-0.27898595
	chr3	138147614	198723053	q22.3 - q29	60575.44	Gain	0.39112198
	chr5	452535	3339587	p15.33	2887.053	Gain	0.79933137
	chr5	4821781	26323106	p15.32 - p14.1	21501.326	Gain	1.3190943
	chr5	26458221	46136124	p14.1 - p11	19677.904	Gain	0.37903106
	chr6	280924	58722020	p25.3 - p11.1	58441.097	Gain	0.6822549
	chr6	62040890	170732033	q11.1 - q27	108691.144	Gain	0.45300707
	chr8	704325	43452795	p23.3 - p11.1	42748.471	Loss	-0.4902571
	chr8	47800500	59930924	q11.1 - q12.1	12130.425	Loss	-0.48667654
	chr8	95953334	96421912	q22.1	468.579	Gain	0.7112047
	chr8	100149140	101042488	q22.2	893.349	Gain	0.70715797
	chr13	47601734	47884379	q14.2	282.646	Loss	-0.9731607
	chr13	71662196	77753402	q21.33 - q22.3	6091.207	Gain	0.25865766
	chr13	78086551	114077122	q31.1 - q34	35990.572	Gain	0.6385409
	chr14	19323579	39769550	q11.2 - q21.1	20445.972	Gain	0.3699005
	chr14	67353536	68064720	q24.1	711.185	Loss	-0.65301025
	chr14	82567057	97692159	q31.1 - q32.2	15125.103	Gain	0.40238407
	chr14	98389742	105399038	q32.2 - q32.33	7009.297	Loss	-0.29231012
	chr15	60386313	75561736	q22.2 - q24.3	15175.424	Loss	-0.25958994
	chr16	142249	34129822	p13.3 - p11.2	33987.574	Loss	-0.2927121
	chr16	45172598	69000216	q11.2 - q22.1	23827.619	Loss	-0.25047174
	chr19	32964337	50587942	q12 - q13.32	17623.606	Gain	0.67724067
	chr20	19822772	26135794	p11.23 - p11.1	6313.023	Gain	0.88525784
	chr20	29352138	62343283	q11.21 - q13.33	32991.146	Gain	0.4821856
	chr21	14510897	46846246	q11.2 - q22.3	32335.35	Gain	0.29306027
	chr22	15476855	19117997	q11.1 - q11.21	3641.143	Gain	0.25138754
039	chr1	749625	116269571	p36.33 - p13.1	115519.947	Loss	-0.4184444
	chr1	116329602	117294210	p13.1	964.609	Gain	0.6600732
	chr1	143700072	247139492	q21.1 - q44	103439.421	Gain	0.5724761

	chr2	32444	84504382	p25.3 - p11.2	84471.939	Loss	-0.4638655
	chr4	63676717	110831576	q13.1 - q25	47154.86	Loss	-0.43523684
	chr6	352231	58722020	p25.3 - p11.1	58369.79	Gain	0.5532328
	chr6	62040890	65719195	q11.1 - q12	3678.306	Gain	0.5898029
	chr6	66215441	170732033	q12 - q27	104516.593	Loss	-0.43614274
	chr8	211611	36792719	p23.3 - p12	36581.109	Loss	-0.4574277
	chr13	25312943	114077122	q12.13 - q34	88764.18	Gain	0.34669873
	chr15	54475616	55149948	q21.3	674.333	Gain	0.4690289
	chr16	46271	34903000	p13.3 - p11.1	34856.73	Loss	-0.38897476
	chr16	45122058	88638764	q11.2 - q24.3	43516.707	Loss	-0.394904
	chr18	4316	14723870	p11.32 - p11.21	14719.555	Loss	-0.48453915
	chr18	16938683	76083117	q11.1 - q23	59144.435	Loss	-0.45771655
040	chr1	145139454	246427587	q21.1 - q44	101288.134	Gain	0.27312812
	chr4	52383858	190706331	q11 - q35.2	138322.474	Loss	-0.30113772
	chr8	514066	35622799	p23.3 - p12	35108.734	Loss	-0.30238837
	chr8	39763621	43452795	p11.22 - p11.1	3689.175	Gain	0.5345245
	chr8	47800500	146201771	q11.1 - q24.3	98401.272	Gain	0.7259951
	chr9	319684	38388671	p24.3 - p13.1	38068.988	Loss	-0.30787748
	chr9	70681459	139972732	q21.11 - q34.3	69291.274	Loss	-0.29451007
	chr10	47125093	135222482	q11.22 - q26.3	88097.39	Loss	-0.33608493
	chr11	62608297	62987465	q12.3	379.169	plification	2.6463237
	chr11	63228921	68323313	q13.1 - q13.2	5094.393	Loss	-0.2883843
	chr11	68406403	69236195	q13.2 - q13.3	829.793	plification	3.1774118
	chr11	69297018	134373617	q13.3 - q25	65076.6	Loss	-0.3070571
	chr12	17928374	23046546	p12.3 - p12.1	5118.173	plification	2.1212335
	chr12	23157120	34148688	p12.1 - p11.1	10991.569	Gain	0.30011368
	chr12	39266576	128726423	q12 - q24.33	89459.848	Gain	0.25072497
	chr13	18194544	114077122	q11 - q34	95882.579	Loss	-0.32036254
	chr14	19323579	105399038	q11.2 - q32.33	86075.46	Loss	-0.33840868
	chr16	45122058	88217382	q11.2 - q24.3	43095.325	Loss	-0.30572253
	chr17	87009	21386319	p13.3 - p11.2	21299.311	Loss	-0.32021204
	chr19	1833336	18415251	p13.3 - p13.11	16581.916	Loss	-0.3066249
	chr22	15533988	49412774	q11.1 - q13.33	33878.787	Gain	0.25520495
041	chr1	24808885	35698566	p36.11 - p34.3	10889.682	Loss	-0.5283149
	chr1	96927190	102023173	p21.3 - p21.1	5095.984	Loss	-0.53859496
	chr1	143700072	247179291	q21.1 - q44	103479.22	Gain	0.5985084
	chr2	14380559	18599899	p24.3 - p24.2	4219.341	Gain	0.3645855
	chr4	61552	48714116	p16.3 - p11	48652.565	Loss	-0.54143435
	chr4	52383858	191004108	q11 - q35.2	138620.251	Loss	-0.5331673
	chr10	138206	38395999	p15.3 - p11.21	38257.794	Gain	0.40638164
	chr11	55316677	134373617	q11 - q25	79056.941	Loss	-0.52179086
	chr12	113433176	132278059	q24.21 - q24.33	18844.884	Loss	-0.60681915
	chr15	18362555	100200996	q11.1 - q26.3	81838.442	Loss	-0.5451067
	chr16	45122058	88638764	q11.2 - q24.3	43516.707	Loss	-0.516388
	chr17	87009	21386319	p13.3 - p11.2	21299.311	Loss	-0.5073153
	chr18	55998040	76083117	q21.32 - q23	20085.078	Gain	0.53011096
	chr20	29352138	62175050	q11.21 - q13.33	32822.913	Gain	0.41895583
	chr22	15950807	49356332	q11.1 - q13.33	33405.526	Loss	-0.568228
	chrX	2719027	58068490	p22.33 - p11.1	55349.464	Gain	0.6086315
	chrX	61848414	154885408	q11.1 - q28	93036.995	Gain	0.8424185
042	chr1	143706582	247118482	q21.1 - q44	103411.901	Gain	0.4493641
	chr5	204737	40668360	p15.33 - p13.1	40463.624	Gain	0.83807015

	chr7	187615	57498383	p22.3 - p11.1	57310.769	Gain	0.47309485
	chr7	62153588	65480434	q11.21	3326.847	Gain	0.4180823
	chr7	66309180	158781397	q11.22 - q36.3	92472.218	Gain	0.7912138
	chr8	211611	43452795	p23.3 - p11.1	43241.185	Gain	0.4849006
	chr8	47800500	62197660	q11.1 - q12.2	14397.161	Gain	0.47854024
	chr8	62364458	146250824	q12.2 - q24.3	83886.367	Gain	1.1118718
	chr9	67911945	140128736	q12 - q34.3	72216.792	Gain	0.4473793
	chr11	69131640	69339391	q13.2 - q13.3	207.752	plification	5.3834295
	chr11	128761187	132583973	q24.3 - q25	3822.787	Gain	0.25370058
	chr12	1183521	34236852	p13.33 - p11.1	33053.332	Gain	0.41826996
	chr12	36858944	132145669	q12 - q24.33	95286.726	Gain	0.40943184
	chr14	19408583	54456716	q11.2 - q22.3	35048.134	Gain	0.4550989
	chr14	55336442	105399038	q22.3 - q32.33	50062.597	Loss	-0.5641929
	chr17	87009	22002704	p13.3 - p11.2	21915.696	Loss	-0.5380085
	chr18	11584789	14918854	p11.21	3334.066	Gain	0.30708623
	chr18	16904187	39656060	q11.1 - q12.3	22751.874	Gain	0.2552012
	chr20	170384	26023841	p13 - p11.1	25853.458	Gain	0.46260628
	chr20	29423641	62363633	q11.21 - q13.33	32939.993	Gain	0.3837607
	chr21	13562263	25984079	q11.2 - q21.3	12421.817	Loss	-0.47826508
	chrX	1091	2666392	p22.33	2665.302	Gain	0.4934338
	chrX	2719027	58068490	p22.33 - p11.1	55349.464	Gain	1.2221369
	chrX	32550995	33337902	p21.1	786.908	Gain	1.7424695
	chrX	61848414	154407365	q11.1 - q28	92558.952	Gain	1.2640661
	chrY	1091	2754930	p11.32 - p11.31	2753.84	Gain	0.48995215
043	chr2	145231594	148447555	q22.3 - q23.1	3215.962	Gain	0.39835802
	chr2	162877641	165308433	q24.2 - q24.3	2430.793	Gain	0.37681058
	chr6	26085971	26380787	p22.2 - p22.1	294.817	Gain	0.42374763
	chr7	79562911	79980120	q21.11	417.21	Gain	0.7493268
	chr8	13324774	17821053	p22	4496.28	Gain	0.2646574
	chr8	106815179	107377047	q23.1	561.869	Gain	0.8644226
	chr11	26677538	28867117	p14.2 - p14.1	2189.58	Gain	0.46659482
044	chr1	749625	44822675	p36.33 - p34.1	44073.051	Loss	-0.5862145
	chr4	28437442	43083854	p15.1 - p13	14646.413	Gain	0.55164886
	chr4	59593671	190706331	q13.1 - q35.2	131112.661	Loss	-0.6063456
	chr5	50094782	73830192	q11.1 - q13.3	23735.411	Gain	0.3722978
	chr5	74678755	180617107	q13.3 - q35.3	105938.353	Loss	-0.6091028
	chr6	68742886	170732033	q12 - q27	101989.148	Loss	-0.60177433
	chr7	187615	56534393	p22.3 - p11.2	56346.779	Loss	-0.6070145
	chr8	211611	43452795	p23.3 - p11.1	43241.185	Loss	-0.5833956
	chr8	57974100	146250824	q12.1 - q24.3	88276.725	Gain	0.46863946
	chr9	116247012	140073968	q32 - q34.3	23826.957	Loss	-0.5736882
	chr10	138206	38585934	p15.3 - p11.21	38447.729	Gain	0.43346295
	chr10	83924095	135254513	q23.1 - q26.3	51330.419	Loss	-0.56592363
	chr11	208365	31129045	p15.5 - p13	30920.681	Loss	-0.5455535
	chr11	132322372	134251838	q25	1929.467	Gain	0.47128877
	chr12	78512297	84146435	q21.2 - q21.31	5634.139	Loss	-0.6083785
	chr13	18361637	26130318	q11 - q12.13	7768.682	Gain	0.4804906
	chr13	26547589	114077122	q12.13 - q34	87529.534	Loss	-0.5950434
	chr14	34142457	105432573	q13.2 - q32.33	71290.117	Loss	-0.608448
	chr15	45742989	100282878	q21.1 - q26.3	54539.89	Gain	0.42302793
	chr16	94314	34903000	p13.3 - p11.1	34808.687	Loss	-0.55833995
	chr16	45122058	88638764	q11.2 - q24.3	43516.707	Loss	-0.5662483

	chr17	87009	16132997	p13.3 - p11.2	16045.989	Loss	-0.5485932
	chr19	488965	24132581	p13.3 - p12	23643.617	Loss	-0.57500166
	chr21	13562263	46847409	q11.2 - q22.3	33285.147	Loss	-0.5839301
045	chr1	749625	37042692	p36.33 - p34.3	36293.068	Loss	-0.3807352
	chr1	37082946	83737823	p34.3 - p31.1	46654.878	Gain	1.4399288
	chr1	37082946	41139076	p34.3 - p34.2	4056.131	Gain	1.8124846
	chr1	83976963	120885791	p31.1 - p11.2	36908.829	Gain	0.36142877
	chr1	143639135	247179291	q21.1 - q44	103540.157	Gain	0.61029524
	chr2	32444	91129998	p25.3 - p11.1	91097.555	Gain	0.33221614
	chr4	61552	48714116	p16.3 - p11	48652.565	Loss	-0.3559077
	chr4	52406688	58953266	q12	6546.579	Loss	-0.38963157
	chr4	61763726	71944173	q13.1 - q13.3	10180.448	Gain	0.8237404
	chr4	72369260	191004108	q13.3 - q35.2	118634.849	Loss	-0.35477045
	chr5	160653456	166652059	q34	5998.604	Gain	0.4106837
	chr6	26125251	29686713	p22.1	3561.463	Gain	0.28363904
	chr6	160446423	170732033	q25.3 - q27	10285.611	Loss	-0.9630571
	chr7	6589410	55242365	p22.1 - p11.2	48652.956	Gain	0.38863194
	chr7	63087010	65883783	q11.21	2796.774	Gain	0.43422168
	chr7	66026478	74123762	q11.21 - q11.23	8097.285	Loss	-0.50253105
	chr8	211611	42137416	p23.3 - p11.21	41925.806	Gain	0.33694693
	chr8	49005004	146201771	q11.21 - q24.3	97196.768	Gain	0.33319938
	chr9	261257	2993970	p24.3 - p24.2	2732.714	Gain	0.48610872
	chr9	28129825	38519673	p21.1 - p13.1	10389.849	Gain	0.5326715
	chr9	70225166	140128736	q13 - q34.3	69903.571	Gain	0.4947936
	chr10	16191107	27569042	p13 - p12.1	11377.936	Loss	-0.4262167
	chr10	90428280	135222482	q23.31 - q26.3	44794.203	Loss	-0.32451025
	chr12	1733888	34081210	p13.33 - p11.1	32347.323	Gain	0.34281
	chr12	36858944	131927866	q12 - q24.33	95068.923	Gain	0.27737835
	chr13	18194544	101851907	q11 - q33.1	83657.364	Loss	-0.33636415
	chr13	102053370	110991573	q33.1 - q34	8938.204	Gain	0.42987764
	chr14	19542388	106260935	q11.2 - q32.33	86718.548	Gain	0.4333574
	chr15	85923322	100187420	q25.3 - q26.3	14264.099	Loss	-0.38944963
	chr16	511780	17205614	p13.3 - p12.3	16693.835	Gain	0.38265938
	chr16	65048878	88638764	q21 - q24.3	23589.887	Loss	-0.39801976
	chr17	210970	21857788	p13.3 - p11.2	21646.819	Loss	-0.34731567
	chr17	23115968	78623230	q11.1 - q25.3	55507.263	Gain	0.38764045
	chr18	51808469	76083117	q21.2 - q23	24274.649	Gain	0.65110654
	chr20	118826	24510026	p13 - p11.21	24391.201	Gain	0.5242379
	chr20	32611751	62323759	q11.22 - q13.33	29712.009	Gain	0.3009463
	chrX	2666344	58068490	p22.33 - p11.1	55402.147	Gain	0.83036095
	chrX	61848414	154284598	q11.1 - q28	92436.185	Gain	0.8727731
	chrY	2716461	9208336	p11.31 - p11.2	6491.876	Deletion	-1.8763756
	chrY	12571053	27176992	q11.21 - q11.23	14605.94	Deletion	-1.1478842
046	chr8	49253853	145782038	q11.21 - q24.3	96528.186	Gain	0.2550288
	chr9	20810273	22509944	p21.3	1699.672	Loss	-0.44443393
	chr11	68358122	69339391	q13.2 - q13.3	981.27	plification	2.1007597
	chr14	77678605	79806473	q24.3 - q31.1	2127.869	Gain	0.49835688
	chr16	24264091	28784206	p12.1 - p11.2	4520.116	Gain	0.43461558
	chr18	2580000	3669368	p11.32 - p11.31	1089.369	Loss	-0.54448605
	chr19	488965	18571943	p13.3 - p13.11	18082.979	Loss	-0.25564766
	chr19	19008377	19987118	p13.11 - p12	978.742	Gain	0.905981
	chr21	41544521	46591026	q22.3	5046.506	Loss	-0.25683108

047	chr5	90112791	92261256	q14.3 - q15	2148.466	Gain	0.4140852
	chr6	26085971	26378998	p22.2 - p22.1	293.028	Gain	0.82072335
	chr7	79562911	81177448	q21.11	1614.538	Gain	0.4826712
	chr11	26677538	28867117	p14.2 - p14.1	2189.58	Gain	0.46621472
	chrX	31996853	35917559	p21.1	3920.707	Gain	0.30126768
	chrY	840228	10473850	p11.32 - p11.2	9633.623	Loss	-0.32954058
	chrY	12593244	57702477	q11.21 - q12	45109.234	Loss	-0.2961974
048	chr1	7917851	10363629	p36.23 - p36.22	2445.779	Loss	-0.3856634
	chr1	144127702	247179291	q21.1 - q44	103051.59	Gain	0.44126666
	chr3	38630778	44125030	p22.2 - p21.33	5494.253	Gain	0.4042747
	chr3	196583022	198149198	q29	1566.177	Loss	-0.3407235
	chr4	175476443	176749296	q34.1 - q34.2	1272.854	Loss	-0.5382725
	chr4	185546008	187054373	q35.1	1508.366	Loss	-0.38812014
	chr5	204737	45865412	p15.33 - p11	45660.676	Gain	0.48169369
	chr5	49774457	180598584	q11.1 - q35.3	130824.128	Gain	0.58618754
	chr6	455657	57183221	p25.3 - p12.1	56727.565	Gain	0.4024991
	chr6	62448434	170732033	q11.1 - q27	108283.6	Loss	-0.51051015
	chr7	187615	57498383	p22.3 - p11.1	57310.769	Gain	0.7198235
	chr7	63087010	158781397	q11.21 - q36.3	95694.388	Gain	0.7073229
	chr8	211611	42817001	p23.3 - p11.21	42605.391	Gain	0.42544308
	chr8	47800500	146250824	q11.1 - q24.3	98450.325	Gain	0.4444874
	chr9	137743959	139407497	q34.3	1663.539	Gain	0.7737038
	chr10	101159997	102777693	q24.2 - q24.31	1617.697	Gain	0.35744742
	chr16	66578792	69247499	q22.1	2668.708	Loss	-0.25422192
	chr17	87009	21386319	p13.3 - p11.2	21299.311	Loss	-0.5165968
	chr19	51784149	63784382	q13.32 - q13.43	12000.234	Gain	0.34568107
	chr20	69521	20582212	p13 - p11.23	20512.692	Loss	-0.48568118
	chr20	20629341	23925553	p11.23 - p11.21	3296.213	Gain	0.4839903
	chr20	29436537	62363633	q11.21 - q13.33	32927.097	Gain	0.35290208
	chr21	13562263	21833149	q11.2 - q21.1	8270.887	Loss	-0.45841697
	chrX	2719027	58068490	p22.33 - p11.1	55349.464	Gain	0.7302319
	chrX	61848414	154407365	q11.1 - q28	92558.952	Gain	0.7619721
049	chr7	187615	35815880	p22.3 - p14.2	35628.266	Gain	0.6083162
	chr7	38254010	56093139	p14.1 - p11.2	17839.13	Gain	0.2525551
	chr17	87009	19791366	p13.3 - p11.2	19704.358	Loss	-0.45310423
	chr17	23115968	78623230	q11.1 - q25.3	55507.263	Gain	0.35813624
	chr20	1056046	24510026	p13 - p11.21	23453.981	Gain	0.29090562
	chrY	13409104	22017318	q11.21 - q11.223	8608.215	Gain	0.37015423
050	chr8	47800500	146091894	q11.1 - q24.3	98291.395	Gain	0.42967317
	chr10	138206	37454940	p15.3 - p11.21	37316.735	Gain	0.38016534
	chr10	42209250	51334085	q11.21 - q11.23	9124.836	Gain	0.4463386
	chr11	67109244	69569280	q13.2 - q13.3	2460.037	plification	2.0914006
	chr11	71708506	133652658	q13.4 - q25	61944.153	Loss	-0.27077574
	chr13	18194544	45992161	q11 - q14.13	27797.618	Loss	-0.30497238
	chr13	86073740	113766081	q31.1 - q34	27692.342	Gain	1.569683
	chr14	24513861	49839419	q12 - q22.1	25325.559	Gain	0.38853267
	chr14	50000502	105354945	q22.1 - q32.33	55354.444	Loss	-0.30709416
	chr18	23010698	31202774	q11.2 - q12.2	8192.077	Gain	0.5412491
	chr22	17890544	49525130	q11.21 - q13.33	31634.587	Gain	0.30083427
	chrX	3069447	31436341	p22.33 - p21.2	28366.895	Loss	-0.28400669
	chrY	2666344	10004221	p11.31 - p11.2	7337.878	Gain	1.067802
	chrY	12571053	57489107	q11.21 - q12	44918.055	Gain	0.7706504

051	chr1	749625	115988694	p36.33 - p13.1	115239.07	Loss	-0.3355396
	chr1	144219515	247179291	q21.1 - q44	102959.777	Gain	0.36997786
	chr3	110147979	119432930	q13.13 - q13.32	9284.952	Gain	0.47038046
	chr4	15025108	26418885	p15.33 - p15.2	11393.778	Loss	-0.35183007
	chr4	81091963	191004108	q21.21 - q35.2	109912.146	Loss	-0.37296528
	chr6	104396044	117422671	q16.3 - q22.2	13026.628	Loss	-0.37183374
	chr7	187615	43106239	p22.3 - p14.1	42918.625	Loss	-0.42798626
	chr8	514066	35286752	p23.3 - p12	34772.687	Loss	-0.35472447
	chr8	70192139	146201771	q13.2 - q24.3	76009.633	Gain	0.50632924
	chr9	319684	33547425	p24.3 - p13.3	33227.742	Loss	-0.3765037
	chr11	208365	40482022	p15.5 - p12	40273.658	Loss	-0.34836322
	chr13	20707346	82392827	q12.11 - q31.1	61685.482	Loss	-0.35065824
	chr13	83872994	113532325	q31.1 - q34	29659.332	Gain	0.3396286
	chr16	46271	33868734	p13.3 - p11.2	33822.464	Loss	-0.41517484
	chr16	45122058	88638764	q11.2 - q24.3	43516.707	Loss	-0.34098363
	chr18	188111	13875315	p11.32 - p11.21	13687.205	Loss	-0.41502675
	chr18	31087146	76083117	q12.2 - q23	44995.972	Loss	-0.38173735
	chr20	1056046	26023841	p13 - p11.1	24967.796	Gain	0.40654624
	chrY	13409104	22017318	q11.21 - q11.223	8608.215	Gain	0.34741843
052	chr1	147490608	155764358	q21.1 - q23.1	8273.751	Gain	0.76948655
	chr1	194561277	196226519	q31.3	1665.243	Gain	0.40112516
	chr3	186991168	187356860	q27.2	365.693	Gain	0.8091418
	chr4	305565	48557847	p16.3 - p12	48252.283	Gain	0.32080787
	chr5	38500180	39391355	p13.1	891.176	Gain	0.5630423
	chr6	593433	57620687	p25.3 - p11.2	57027.255	Gain	0.4913414
	chr6	62040890	111261365	q11.1 - q21	49220.476	Gain	0.41380912
	chr8	514066	43452795	p23.3 - p11.1	42938.73	Loss	-0.27430204
	chr10	42289771	65935382	q11.21 - q21.3	23645.612	Gain	0.4969858
	chr13	67606795	114077122	q21.33 - q34	46470.328	Gain	0.9565668
	chr17	27685878	28343878	q11.2	658.001	Gain	1.2063913
	chrX	516161	58068490	p22.33 - p11.1	57552.33	Gain	0.397087
	chrX	62792220	154885408	q11.1 - q28	92093.189	Gain	0.40681276
	chrY	94062	10473850	p11.32 - p11.2	10379.789	Gain	0.33144617
	chrY	12571053	57744608	q11.21 - q12	45173.556	Gain	0.3945079
053	chr1	144451955	175364850	q21.1 - q25.2	30912.896	Gain	0.929483
	chr1	175461745	247118482	q25.2 - q44	71656.738	Gain	0.29262632
	chr3	345339	9109462	p26.3 - p25.3	8764.124	Gain	0.31139576
	chr4	52406688	191004108	q12 - q35.2	138597.421	Loss	-0.2961069
	chr5	204737	13883744	p15.33 - p15.2	13679.008	Gain	0.27991104
	chr7	78619146	86205180	q21.11 - q21.12	7586.035	Gain	0.3374292
	chr8	704325	43452795	p23.3 - p11.1	42748.471	Loss	-0.3100727
	chr8	92671861	137621382	q21.3 - q24.23	44949.522	Gain	1.5575106
	chr9	124559827	132287078	q33.2 - q34.11	7727.252	Loss	-0.40541378
	chr10	43602229	57140296	q11.21 - q21.1	13538.068	Gain	0.38069326
	chr10	60858683	134603929	q21.1 - q26.3	73745.247	Loss	-0.30576107
	chr13	18679724	104243768	q12.11 - q33.2	85564.045	Loss	-0.282875
	chr14	28234818	105013921	q12 - q32.33	76779.104	Loss	-0.356505
	chr15	47718086	100187420	q21.2 - q26.3	52469.335	Loss	-0.26065502
	chr16	45122058	88638764	q11.2 - q24.3	43516.707	Loss	-0.29376507
	chr19	1856897	20738499	p13.3 - p12	18881.603	Loss	-0.3772925
	chr20	1056046	23295721	p13 - p11.21	22239.676	Gain	0.3318254
	chr21	18677896	25828359	q21.1 - q21.3	7150.464	Gain	0.46187288

	chrY	12571053	22017318	q11.21 - q11.223	9446.266	Gain	0.3142377
054	chr1	144127702	172976169	q21.1 - q25.1	28848.468	Gain	0.25506926
	chr7	78474418	85739981	q21.11	7265.564	Gain	0.2907588
055	chr3	176437712	179192769	q26.31 - q26.32	2755.058	Gain	0.4000561
	chr4	54873909	139335807	q12 - q28.3	84461.899	Loss	-0.27103886
	chr4	177020890	191004108	q34.2 - q35.2	13983.219	Loss	-0.27675048
	chr5	924638	45865412	p15.33 - p11	44940.775	Gain	0.27714822
	chr6	630345	55026679	p25.3 - p12.1	54396.335	Gain	0.4160676
	chr7	187615	56611349	p22.3 - p11.2	56423.735	Gain	0.3640281
	chr7	66368051	94504147	q11.22 - q21.3	28136.097	Gain	0.39017636
	chr8	704325	43291296	p23.3 - p11.1	42586.972	Loss	-0.28635466
	chr8	79170737	146201771	q21.12 - q24.3	67031.035	Gain	0.47209433
	chr16	46271	34903000	p13.3 - p11.1	34856.73	Loss	-0.27739012
	chr16	45122058	88638764	q11.2 - q24.3	43516.707	Loss	-0.25723687
	chr17	210970	22129948	p13.3 - p11.1	21918.979	Loss	-0.26759222
	chrY	15005207	21559609	q11.221 - q11.223	6554.403	Gain	0.28145736
056	chr1	144127702	247118482	q21.1 - q44	102990.781	Gain	0.38332298
	chr2	137298749	148447555	q22.1 - q23.1	11148.807	Gain	0.27880546
	chr3	58527374	87917144	p14.2 - p11.2	29389.771	Gain	0.3091057
	chr4	93445209	98609318	q22.1 - q22.3	5164.11	Gain	0.37780064
	chr5	873365	45865412	p15.33 - p11	44992.048	Gain	0.43030998
	chr5	49725929	180417981	q11.1 - q35.3	130692.053	Gain	0.36655158
	chr6	593433	58722020	p25.3 - p11.1	58128.588	Gain	0.6277187
	chr6	62040890	64139231	q11.1 - q12	2098.342	Gain	0.79698783
	chr6	64633346	71194779	q12 - q13	6561.434	Loss	-0.36546034
	chr6	71355115	74029317	q13	2674.203	Gain	0.67473763
	chr6	74191717	170732033	q13 - q27	96540.317	Loss	-0.37531304
	chr7	7010835	55242365	p22.1 - p11.2	48231.531	Gain	0.40397984
	chr7	66461831	158781397	q11.22 - q36.3	92319.567	Gain	0.3786743
	chr8	211611	43291296	p23.3 - p11.1	43079.686	Loss	-0.44699186
	chr8	47800500	146250824	q11.1 - q24.3	98450.325	Gain	0.7529307
	chr10	53416626	60792202	q21.1	7375.577	Gain	0.2709573
	chr11	21123640	31074662	p15.1 - p13	9951.023	Gain	0.2893032
	chr11	128761187	133687965	q24.3 - q25	4926.779	Gain	0.29156882
	chr12	47300434	50013885	q13.11 - q13.13	2713.452	Loss	-0.27566147
	chr12	119903214	122919033	q24.31	3015.82	Loss	-0.25638
	chr16	66567072	69247499	q22.1	2680.428	Loss	-0.30343452
	chr17	87009	20036515	p13.3 - p11.2	19949.507	Loss	-0.44380602
	chrY	1753446	10622121	p11.31 - p11.2	8868.676	Loss	-0.28791976
	chrY	12627138	57586758	q11.21 - q12	44959.621	Loss	-0.35395023
057	chr1	144219515	246630459	q21.1 - q44	102410.945	Gain	0.58061206
	chr7	187615	57498383	p22.3 - p11.1	57310.769	Gain	0.34275317
	chr11	69806894	84356658	q13.3 - q14.1	14549.765	Gain	0.3244573
	chr19	37856935	40737421	q13.11 - q13.12	2880.487	Gain	1.4093889
	chr20	29716147	59244861	q11.21 - q13.33	29528.715	Gain	0.35445574
	chr22	15476855	49451468	q11.1 - q13.33	33974.614	Gain	0.28359187
058	chr1	749625	69678673	p36.33 - p31.1	68929.049	Loss	-0.5154017
	chr1	143700072	176067040	q21.1 - q25.2	32366.969	Gain	0.83763236
	chr3	68949	76880005	p26.3 - p12.3	76811.057	Loss	-0.45248634
	chr3	77084744	87917144	p12.3 - p11.2	10832.401	Gain	0.31983173
	chr5	204737	14417548	p15.33 - p15.2	14212.812	Gain	0.27406496
	chr5	17685501	46136124	p15.1 - p11	28450.624	Gain	0.5891924

	chr5	49774457	59142525	q11.1 - q12.1	9368.069	Gain	0.58655125
	chr5	59872475	180617107	q12.1 - q35.3	120744.633	Loss	-0.4885419
	chr6	79628347	170302324	q14.1 - q27	90673.978	Loss	-0.46134362
	chr8	211611	42849186	p23.3 - p11.21	42637.576	Gain	0.33649075
	chr8	47800500	146250824	q11.1 - q24.3	98450.325	Gain	0.7372554
	chr9	319684	38480834	p24.3 - p13.1	38161.151	Loss	-0.4509342
	chr10	183492	36844313	p15.3 - p11.21	36660.822	Loss	-0.36377934
	chr10	55256725	135222482	q21.1 - q26.3	79965.758	Loss	-0.4845093
	chr11	303837	51289318	p15.5 - p11.12	50985.482	Loss	-0.43178362
	chr11	54893780	134373617	q11 - q25	79479.838	Loss	-0.44214866
	chr13	18194544	80808086	q11 - q31.1	62613.543	Loss	-0.48328465
	chr14	60251793	105399038	q23.1 - q32.33	45147.246	Loss	-0.5139322
	chr16	45122058	88563897	q11.2 - q24.3	43441.84	Loss	-0.47541764
	chr17	87009	17592700	p13.3 - p11.2	17505.692	Loss	-0.51419544
	chr18	61643342	76083117	q22.1 - q23	14439.776	Loss	-0.4453836
	chr19	278273	14941011	p13.3 - p13.12	14662.739	Loss	-0.5560811
	chr20	15647327	25235554	p12.1 - p11.21	9588.228	Gain	0.7656255
	chr20	29716147	62363633	q11.21 - q13.33	32647.487	Gain	0.4488149
	chr21	13783127	46433516	q11.2 - q22.3	32650.39	Loss	-0.4872396
	chr22	22736990	48667712	q11.23 - q13.33	25930.723	Loss	-0.25175157
	chrY	2754871	10511314	p11.31 - p11.2	7756.444	Gain	0.8496273
	chrY	12571053	22395855	q11.21 - q11.223	9824.803	Gain	0.5880962
059	chr1	749625	120138229	p36.33 - p12	119388.605	Loss	-0.31057873
	chr1	143639135	246505088	q21.1 - q44	102865.954	Gain	0.6853669
	chr2	32444	89223022	p25.3 - p11.2	89190.579	Gain	0.42684618
	chr2	95145279	241301905	q11.1 - q37.3	146156.627	Gain	0.31856224
	chr3	99317876	144031315	q11.2 - q23	44713.44	Loss	-0.32385394
	chr4	61552	48758058	p16.3 - p11	48696.507	Loss	-0.31689167
	chr4	52383858	190706331	q11 - q35.2	138322.474	Loss	-0.3050357
	chr5	204737	46136124	p15.33 - p11	45931.388	Gain	0.7857007
	chr5	52022330	54067232	q11.2	2044.903	Gain	1.1100473
	chr5	178922234	180598584	q35.3	1676.351	Gain	0.9041691
	chr6	26085971	26393392	p22.2 - p22.1	307.422	Gain	0.5360878
	chr6	30342921	58722020	p21.33 - p11.1	28379.1	Gain	0.5282561
	chr6	62040890	170707926	q11.1 - q27	108667.037	Gain	0.5289044
	chr7	524935	57327440	p22.3 - p11.2	56802.506	Gain	0.38259748
	chr7	63087010	158781397	q11.21 - q36.3	95694.388	Gain	0.37306425
	chr8	53770229	63008458	q11.23 - q12.3	9238.23	Loss	-0.8511059
	chr8	115354884	146250824	q23.3 - q24.3	30895.941	Gain	0.5476073
	chr9	319684	30737639	p24.3 - p21.1	30417.956	Loss	-0.80757695
	chr9	31134867	39146954	p21.1 - p13.1	8012.088	Gain	1.1395985
	chr9	70225166	74312976	q13 - q21.13	4087.811	Gain	0.5554988
	chr10	27915022	38468053	p12.1 - p11.21	10553.032	Gain	0.34570345
	chr10	49125130	91166310	q11.22 - q23.31	42041.181	Loss	-0.3166706
	chr11	208365	21582758	p15.5 - p15.1	21374.394	Loss	-0.31153816
	chr11	22093724	31284473	p14.3 - p13	9190.75	Gain	0.4870492
	chr11	55316677	80494261	q11 - q14.1	25177.585	Gain	0.33869633
	chr12	1089733	33527983	p13.33 - p11.1	32438.251	Gain	0.31858888
	chr13	18194544	103076962	q11 - q33.1	84882.419	Loss	-0.30466536
	chr14	19323579	105399038	q11.2 - q32.33	86075.46	Loss	-0.337501
	chr15	44653913	100282878	q21.1 - q26.3	55628.966	Gain	0.36389598
	chr16	27646290	34903000	p11.2 - p11.1	7256.711	Loss	-0.78567445

	chr16	45122058	52440914	q11.2 - q12.2	7318.857	Loss	-0.37845767
	chr16	53523174	76571323	q12.2 - q23.1	23048.15	Gain	0.30456227
	chr16	79654949	88638764	q23.2 - q24.3	8983.816	Loss	-0.37766448
	chr17	22879058	26339208	q11.1 - q11.2	3460.151	Gain	0.28082064
	chr18	23010698	75905054	q11.2 - q23	52894.357	Gain	0.33495593
	chr19	1856897	13337228	p13.3 - p13.13	11480.332	Loss	-0.34424216
	chr20	5943588	26023841	p12.3 - p11.1	20080.254	Gain	0.3342922
	chr21	13562263	46591026	q11.2 - q22.3	33028.764	Loss	-0.3249455
	chrX	1091	31325624	p22.33 - p21.2	31324.534	Loss	-0.26580557
	chrX	36180542	58068490	p21.1 - p11.1	21887.949	Gain	0.3532365
	chrX	91767271	154561665	q21.31 - q28	62794.395	Gain	0.44911867
	chrX	145516682	153943360	q27.3 - q28	8426.679	Gain	0.7748103
	chrY	2716461	10511314	p11.31 - p11.2	7794.854	Deletion	-1.6780531
	chrY	12593244	27176992	q11.21 - q11.23	14583.749	Deletion	-1.0913929
	chrY	16764847	19900891	q11.221 - q11.222	3136.045	Loss	-0.42298663
060	chr5	38542440	39391355	p13.1	848.916	Gain	0.602132
	chr8	47987961	146250824	q11.1 - q24.3	98262.864	Gain	0.85348785
	chr9	1356167	7598084	p24.3 - p24.1	6241.918	Loss	-0.29632533
	chr9	36153040	37988839	p13.3 - p13.2	1835.8	Loss	-0.419867
	chr11	26677538	28867117	p14.2 - p14.1	2189.58	Gain	0.4957817
	chr11	68654447	69297077	q13.2 - q13.3	642.631	plification	2.1698868
	chr11	74557838	77881810	q13.4 - q14.1	3323.973	Gain	1.1793231
	chr15	47463650	47739194	q21.1 - q21.2	275.545	plification	2.7158678
	chr15	50856559	51695705	q21.2 - q21.3	839.147	plification	2.6690295
	chr19	557030	17125814	p13.3 - p13.11	16568.785	Loss	-0.29685244
	chr19	18379148	23924914	p13.11 - p12	5545.767	Gain	0.27992743
	chr19	59097593	63784382	q13.41 - q13.43	4686.79	Gain	0.28859565
	chrX	62775409	153656695	q11.1 - q28	90881.287	Gain	0.27211887
061	chr2	32444	22453474	p25.3 - p24.1	22421.031	Gain	1.5789202
	chr2	134727485	200937988	q21.2 - q33.1	66210.504	Loss	-0.5705012
	chr2	211013677	241558319	q34 - q37.3	30544.643	Gain	0.31434253
	chr4	53755607	191004108	q12 - q35.2	137248.502	Loss	-0.58294535
	chr7	78474418	86827197	q21.11 - q21.12	8352.78	Gain	0.38241705
	chr8	617655	43452795	p23.3 - p11.1	42835.141	Loss	-0.6453068
	chr8	67076431	145748725	q13.1 - q24.3	78672.295	Gain	0.54356337
	chr9	319684	39146954	p24.3 - p13.1	38827.271	Loss	-0.60353047
	chr9	70327267	139972732	q13 - q34.3	69645.466	Loss	-0.6110417
	chr11	56948057	63669241	q12.1 - q13.1	6721.185	Gain	0.77491325
	chr11	66780416	68654505	q13.1 - q13.2	1874.09	Gain	0.73289686
	chr11	68684682	70012823	q13.2 - q13.3	1328.142	plification	2.4457629
	chr11	70110393	72079612	q13.3 - q13.4	1969.22	Loss	-0.60222244
	chr12	13416672	25048970	p13.1 - p12.1	11632.299	Gain	0.25682303
	chr13	18194544	114077122	q11 - q34	95882.579	Loss	-0.61524105
	chr14	77678605	82567116	q24.3 - q31.1	4888.512	Gain	0.42272347
	chr16	45122058	88638764	q11.2 - q24.3	43516.707	Loss	-0.6179804
	chr17	87009	21857788	p13.3 - p11.2	21770.78	Loss	-0.66378003
	chr17	23115968	78623230	q11.1 - q25.3	55507.263	Gain	0.3240431
	chr18	4316	12464137	p11.32 - p11.21	12459.822	Gain	0.44441682
	chr18	17701331	76083117	q11.2 - q23	58381.787	Gain	0.54189485
	chrY	2716461	10511314	p11.31 - p11.2	7794.854	Deletion	-2.0397034
	chrY	12593244	27176992	q11.21 - q11.23	14583.749	Deletion	-1.227741
062	chr1	144219515	247179291	q21.1 - q44	102959.777	Gain	0.3835262

	chr3	49382828	89920060	p21.31 - p11.1	40537.233	Gain	0.3439781
	chr5	204737	45681293	p15.33 - p12	45476.557	Gain	0.60756564
	chr6	77499382	102689059	q14.1 - q16.3	25189.678	Gain	0.2715387
	chr9	8303246	36090542	p24.1 - p13.3	27787.297	Gain	0.26962852
	chr12	6204888	28301445	p13.31 - p11.22	22096.558	Loss	-0.26451448
	chr12	46755516	116711461	q13.11 - q24.23	69955.946	Gain	0.26245922
	chr20	57950899	62320720	q13.33	4369.822	Gain	0.6844187
	chrX	9422784	57943289	p22.31 - p11.1	48520.506	Gain	0.44222692
	chrX	62078907	154885408	q11.1 - q28	92806.502	Gain	0.5271964
	chrY	13409104	57744608	q11.21 - q12	44335.505	Gain	0.56971925
063	chr1	9426108	11641053	p36.22	2214.946	Loss	-0.615529
	chr1	144127702	246630459	q21.1 - q44	102502.758	Gain	0.4281011
	chr2	32444	46267073	p25.3 - p21	46234.63	Gain	0.4310194
	chr4	57536993	64351937	q12 - q13.1	6814.945	Gain	0.4943355
	chr4	66074224	189257416	q13.1 - q35.2	123183.193	Loss	-0.40855375
	chr5	452535	45865412	p15.33 - p11	45412.878	Gain	0.5082525
	chr5	51750061	126627012	q11.2 - q23.2	74876.952	Loss	-0.4131423
	chr8	1307209	35227207	p23.3 - p12	33919.999	Loss	-0.40275684
	chr8	105857203	144753629	q22.3 - q24.3	38896.427	Gain	0.4507403
	chr9	261257	33463272	p24.3 - p13.3	33202.016	Loss	-0.3745413
	chr9	34600488	35804265	p13.3	1203.778	Gain	0.5271808
	chr10	53883356	61282457	q21.1 - q21.2	7399.102	Gain	0.3661435
	chr11	274838	14301149	p15.5 - p15.2	14026.312	Gain	0.39982712
	chr13	39671015	52839623	q14.11 - q21.1	13168.609	Loss	-0.43819728
	chr13	56770498	114029609	q21.1 - q34	57259.112	Gain	0.46643683
	chr16	58897564	64984206	q21	6086.643	Gain	0.49462396
	chr16	78963618	88530033	q23.2 - q24.3	9566.416	Loss	-0.4629108
	chr17	210970	21096276	p13.3 - p11.2	20885.307	Loss	-0.38919827
	chr21	13783127	46479831	q11.2 - q22.3	32696.705	Loss	-0.3891874
064	chr2	208339551	242690037	q33.3 - q37.3	34350.487	Loss	-0.43192
	chr4	103432806	191004108	q24 - q35.2	87571.303	Loss	-0.48207575
	chr10	1875886	11509401	p15.3 - p14	9633.516	Gain	0.39418176
	chr12	50013826	131927866	q13.13 - q24.33	81914.041	Gain	0.40524033
	chr13	34186645	59405843	q13.2 - q21.2	25219.199	Loss	-0.45485887
	chr13	93888855	113904967	q32.1 - q34	20016.113	Gain	0.4293558
	chr14	62759793	105399038	q23.2 - q32.33	42639.246	Loss	-0.3516456
	chr15	72408613	100187420	q24.1 - q26.3	27778.808	Loss	-0.47903883
	chr16	63579600	88156510	q21 - q24.3	24576.911	Loss	-0.46723926
	chr18	38618066	76083117	q12.3 - q23	37465.052	Loss	-0.4872322
	chr19	42850559	63618994	q13.12 - q13.43	20768.436	Loss	-0.470345
	chr22	28972821	48901100	q12.2 - q13.33	19928.28	Loss	-0.38816488
	chrX	2666344	58068490	p22.33 - p11.1	55402.147	Gain	0.8207735
	chrX	61848414	153814457	q11.1 - q28	91966.044	Gain	0.5572226
	chrY	662815	8719517	p11.32 - p11.2	8056.703	Loss	-0.5754444
065	chr1	5414227	93461630	p36.31 - p22.1	88047.404	Loss	-0.34132686
	chr4	7298021	35612378	p16.1 - p14	28314.358	Gain	0.2724987
	chr8	369418	43051302	p23.3 - p11.21	42681.885	Loss	-0.42770052
	chr8	47800500	57576878	q11.1 - q12.1	9776.379	Loss	-0.45038787
	chr8	60180511	131244656	q12.1 - q24.21	71064.146	Gain	1.0838561
	chr8	132115886	143689477	q24.22 - q24.3	11573.592	Loss	-0.38154855
	chr13	18194544	113878310	q11 - q34	95683.767	Gain	0.32783803
	chr16	1463735	34242154	p13.3 - p11.2	32778.42	Loss	-0.26310697

	chr17	264550	21096276	p13.3 - p11.2	20831.727	Loss	-0.38936946
	chr17	47528203	78536478	q21.33 - q25.3	31008.276	Gain	0.502402
	chr19	372537	18531324	p13.3 - p13.11	18158.788	Loss	-0.4230459
	chr20	445015	24920596	p13 - p11.21	24475.582	Gain	0.31865224
	chr20	32335985	62320720	q11.22 - q13.33	29984.736	Gain	0.2688483
	chr22	35311810	49525130	q12.3 - q13.33	14213.321	Gain	0.43253598
066	chr1	144127702	244692977	q21.1 - q44	100565.276	Gain	0.2611555
067	chr1	235417871	235671351	q43	253.481	Deletion	-1.5599763
	chr2	74669	91129998	p25.3 - p11.1	91055.33	Loss	-0.323478
	chr2	94892766	242579551	q11.1 - q37.3	147686.786	Loss	-0.26990658
	chr3	71656519	75850282	p14.1 - p12.3	4193.764	Loss	-0.7262012
	chr4	14125671	39229425	p15.33 - p14	25103.755	Gain	0.30898133
	chr4	52383858	191004108	q11 - q35.2	138620.251	Loss	-0.26933303
	chr5	49725929	97732938	q11.1 - q21.1	48007.01	Loss	-0.6783204
	chr6	149214213	170700061	q25.1 - q27	21485.849	Loss	-0.28507465
	chr7	3625418	6837468	p22.2 - p22.1	3212.051	Loss	-0.5836716
	chr7	62153588	77852987	q11.21 - q21.11	15699.4	Loss	-0.32846308
	chr8	617655	43452795	p23.3 - p11.1	42835.141	Loss	-0.6808458
	chr8	57456630	146250824	q12.1 - q24.3	88794.195	Gain	0.5545529
	chr9	70341555	137898102	q13 - q34.3	67556.548	Loss	-0.30431792
	chr10	126163791	135222482	q26.13 - q26.3	9058.692	Loss	-0.7143052
	chr11	643155	47571420	p15.5 - p11.2	46928.266	Gain	0.6853613
	chr11	66780416	107231749	q13.1 - q22.3	40451.334	Gain	0.5840629
	chr12	100682	32730005	p13.33 - p11.21	32629.324	Loss	-0.4701469
	chr13	18194544	114077122	q11 - q34	95882.579	Loss	-0.28261268
	chr14	20534819	29264535	q11.2 - q12	8729.717	Gain	0.26142892
	chr14	29621098	105399038	q12 - q32.33	75777.941	Loss	-0.34498566
	chr15	18362555	63634367	q11.1 - q22.31	45271.813	Loss	-0.31703416
	chr16	46271	34634745	p13.3 - p11.1	34588.475	Loss	-0.36784852
	chr16	45122058	88565680	q11.2 - q24.3	43443.623	Loss	-0.30706978
	chr18	29493255	31452984	q12.1 - q12.2	1959.73	Gain	0.98489857
	chr19	15440434	23164126	p13.12 - p12	7723.693	Loss	-0.253695
	chr21	19154972	33799862	q21.1 - q22.11	14644.891	Loss	-0.66692585
	chr22	15950807	49230320	q11.1 - q13.33	33279.514	Loss	-0.3521914
	chrX	1753446	56609305	p22.33 - p11.1	54855.86	Loss	-0.29346126
	chrY	6899085	10379571	p11.2	3480.487	Gain	1.0832884
	chrY	12571053	22916805	q11.21 - q11.223	10345.753	Gain	0.79538256
068	Normal						
069	chr1	144127702	190575835	q21.1 - q31.2	46448.134	Gain	0.46698248
	chr2	45611646	52960573	p21 - p16.2	7348.928	Gain	0.5487372
	chr2	100589941	242656032	q11.2 - q37.3	142066.092	Loss	-0.4431958
	chr3	95136554	113025638	q11.2 - q13.2	17889.085	Gain	0.2768122
	chr3	196725191	198149198	q29	1424.008	Loss	-0.53555954
	chr4	64471364	189902947	q13.1 - q35.2	125431.584	Loss	-0.44230416
	chr5	38629838	43591995	p13.1 - p12	4962.158	Gain	0.36832026
	chr5	143174863	180617107	q32 - q35.3	37442.245	Loss	-0.43157
	chr7	82298568	147835182	q21.11 - q36.1	65536.615	Gain	0.6593504
	chr7	148576685	158781397	q36.1 - q36.3	10204.713	Gain	0.3246711
	chr8	211611	42971936	p23.3 - p11.21	42760.326	Gain	0.3939672
	chr8	47800500	140916747	q11.1 - q24.3	93116.248	Gain	0.2589603
	chr9	319684	30134461	p24.3 - p21.1	29814.778	Loss	-0.4479402
	chr9	71552270	122900544	q21.11 - q33.2	51348.275	Gain	0.39724922

	chr9	135646710	139407497	q34.2 - q34.3	3760.788	Gain	0.494015
	chr10	49955253	135222482	q11.22 - q26.3	85267.23	Loss	-0.41925243
	chr11	84217254	134373617	q14.1 - q25	50156.364	Loss	-0.4011219
	chr13	18194544	83973354	q11 - q31.1	65778.811	Loss	-0.44360235
	chr13	86608058	91665650	q31.2 - q31.3	5057.593	Gain	0.48778626
	chr13	93021903	95469334	q31.3 - q32.1	2447.432	Loss	-0.49939838
	chr14	19651899	28217423	q11.2 - q12	8565.525	Gain	0.44732594
	chr15	18362555	100282878	q11.1 - q26.3	81920.324	Loss	-0.43584085
	chr16	45122058	54336480	q11.2 - q12.2	9214.423	Gain	0.5340897
	chr16	54390060	88621982	q12.2 - q24.3	34231.923	Loss	-0.40625796
	chr18	22882431	37961503	q11.2 - q12.3	15079.073	Gain	0.26217058
	chr19	48915972	62879610	q13.31 - q13.43	13963.639	Loss	-0.5236274
	chr21	13562263	46591026	q11.2 - q22.3	33028.764	Loss	-0.4435752
	chr22	22712211	49034395	q11.23 - q13.33	26322.185	Loss	-0.51276803
	chrX	31325565	40615981	p21.2 - p11.4	9290.417	Gain	0.30164257
	chrX	144705564	144933325	q27.3	227.762	Deletion	-1.3277034
070	chr1	144188986	247118482	q21.1 - q44	102929.497	Gain	0.2726323
	chr3	194961377	198149198	q29	3187.822	Loss	-0.30450258
	chr7	78474418	86827197	q21.11 - q21.12	8352.78	Gain	0.32797605
	chr11	21056477	30908237	p15.1 - p14.1	9851.761	Gain	0.4388491
	chr11	65950211	79926646	q13.1 - q14.1	13976.436	Gain	0.46428058
	chr12	64533555	74191063	q14.3 - q21.2	9657.509	Gain	0.31333935
071	chr1	749625	35037052	p36.33 - p34.3	34287.428	Loss	-0.488583
	chr1	35095023	44252210	p34.3 - p34.1	9157.188	Gain	0.36930224
	chr1	144127702	171968284	q21.1 - q25.1	27840.583	Gain	0.3538947
	chr2	74669	18634232	p25.3 - p24.2	18559.564	Gain	0.30396596
	chr2	109395739	186367935	q13 - q32.1	76972.197	Loss	-0.44136354
	chr4	165525969	189986988	q32.3 - q35.2	24461.02	Loss	-0.47594574
	chr5	924638	45865412	p15.33 - p11	44940.775	Gain	0.34803465
	chr6	65760879	170700061	q12 - q27	104939.183	Loss	-0.40907735
	chr7	4082319	6681137	p22.2 - p22.1	2598.819	Loss	-0.68533516
	chr7	31927003	56234855	p14.3 - p11.2	24307.853	Loss	-0.40030026
	chr7	62153588	119689593	q11.21 - q31.31	57536.006	Loss	-0.4290611
	chr7	140266786	157992030	q34 - q36.3	17725.245	Gain	0.35175654
	chr8	211611	43291296	p23.3 - p11.1	43079.686	Loss	-0.45359293
	chr8	47987961	145836174	q11.1 - q24.3	97848.214	Gain	0.8736751
	chr9	319684	38480834	p24.3 - p13.1	38161.151	Loss	-0.37417367
	chr9	70341555	137532016	q13 - q34.3	67190.462	Loss	-0.41813707
	chr12	90084464	101808595	q21.33 - q23.2	11724.132	Loss	-0.39866394
	chr13	18194544	114077122	q11 - q34	95882.579	Loss	-0.42856285
	chr16	94314	34634745	p13.3 - p11.1	34540.432	Loss	-0.42200154
	chr16	45122058	88523261	q11.2 - q24.3	43401.204	Loss	-0.37075537
	chr19	278273	23783753	p13.3 - p12	23505.481	Loss	-0.5004811
	chr22	16023930	49525130	q11.1 - q13.33	33501.201	Loss	-0.46528104
	chrY	2783745	10473850	p11.31 - p11.2	7690.106	Deletion	-1.108763
	chrY	12593244	57586758	q11.21 - q12	44993.515	Loss	-0.7950775
072	chr1	6334098	10363629	p36.31 - p36.22	4029.532	Loss	-0.38279888
	chr1	144124745	244616883	q21.1 - q44	100492.139	Gain	0.29517707
	chr5	3110571	41313053	p15.33 - p13.1	38202.483	Gain	0.3504717
	chr8	49101338	146250824	q11.21 - q24.3	97149.487	Gain	0.5358468
	chr11	1369563	46364611	p15.5 - p11.2	44995.049	Gain	0.258075
	chr15	38906413	42653004	q15.1 - q15.3	3746.592	Loss	-0.29179838

073	chr2	142109308	148447555	q22.1 - q23.1	6338.248	Gain	0.4303915
	chr3	312899	90102363	p26.3 - p11.1	89789.465	Gain	0.46091542
	chr3	95136554	196608477	q11.2 - q29	101471.924	Gain	0.46496555
	chr4	12322518	35612378	p15.33 - p14	23289.861	Gain	0.26394746
	chr5	1852098	45865412	p15.33 - p11	44013.315	Gain	0.26134706
	chr6	13138755	55819395	p24.1 - p12.1	42680.641	Gain	0.2616538
	chr6	62040890	169943849	q11.1 - q27	107902.96	Gain	0.25177935
	chr7	7104046	54583466	p22.1 - p11.2	47479.421	Gain	0.30161706
	chr7	77892776	158781397	q21.11 - q36.3	80888.622	Gain	0.44688606
	chr8	50152959	145748725	q11.21 - q24.3	95595.767	Gain	0.33415928
	chr11	643155	49489219	p15.5 - p11.12	48846.065	Gain	0.5035518
	chr11	55238200	63995143	q11 - q13.1	8756.944	Gain	0.39134112
	chr11	66352349	73036100	q13.1 - q13.4	6683.752	Gain	1.0306814
	chr12	1237942	33527983	p13.33 - p11.1	32290.042	Gain	0.47393382
	chr12	36858944	132217409	q12 - q24.33	95358.466	Gain	0.4393714
	chr14	20534819	21208966	q11.2	674.148	Gain	0.7456491
	chr18	22882431	42429261	q11.2 - q21.1	19546.831	Gain	0.26340023
	chr20	1095541	25227283	p13 - p11.21	24131.743	Gain	0.2832724
	chr20	35839497	62320720	q11.23 - q13.33	26481.224	Gain	0.39906016
	chrX	62775409	153667892	q11.1 - q28	90892.484	Gain	0.26573676
	chrY	12627138	57586758	q11.21 - q12	44959.621	Loss	-0.3372168
074	chr1	143639135	247179291	q21.1 - q44	103540.157	Gain	0.7623458
	chr3	95136554	110539554	q11.2 - q13.13	15403.001	Gain	0.25349945
	chr4	119245596	190706331	q26 - q35.2	71460.736	Loss	-0.6772372
	chr5	924638	29643288	p15.33 - p13.3	28718.651	Gain	0.8168443
	chr6	1051749	31215113	p25.3 - p21.33	30163.365	Gain	0.4482248
	chr7	62471018	90535410	q11.21 - q21.13	28064.393	Loss	-0.73308194
	chr9	261257	14297079	p24.3 - p22.3	14035.823	Gain	0.46240872
	chr9	103127012	103795042	q31.1	668.031	Gain	0.8734968
	chr10	138206	34984198	p15.3 - p11.21	34845.993	Gain	0.449208
	chr10	75692999	135254513	q22.2 - q26.3	59561.515	Loss	-0.63871306
	chr13	25354169	80750869	q12.13 - q31.1	55396.701	Loss	-0.6469784
	chr13	99916079	113904967	q32.3 - q34	13988.889	Gain	0.4802122
	chr16	45122058	88638764	q11.2 - q24.3	43516.707	Loss	-0.6565677
	chr17	87009	16286061	p13.3 - p11.2	16199.053	Loss	-0.68482983
	chr17	35138973	78623230	q12 - q25.3	43484.258	Gain	0.39399114
	chr18	22852184	31758679	q11.2 - q12.2	8906.496	Gain	0.3327163
	chr18	45622276	76083117	q21.1 - q23	30460.842	Loss	-0.69690734
	chr19	278273	11296163	p13.3 - p13.2	11017.891	Loss	-0.6616492
075	chr1	49240765	67943795	p33 - p31.3	18703.031	Loss	-0.45043164
	chr1	96405184	102264532	p21.3 - p21.1	5859.349	Loss	-0.5275994
	chr1	193490287	246505088	q31.3 - q44	53014.802	Gain	0.44634238
	chr5	88268343	180617107	q14.3 - q35.3	92348.765	Loss	-0.45656142
	chr6	8811135	56155401	p24.3 - p12.1	47344.267	Gain	0.38226658
	chr6	62448434	170382576	q11.1 - q27	107934.143	Loss	-0.44631967
	chr8	211611	43291296	p23.3 - p11.1	43079.686	Loss	-0.4421216
	chr8	48067862	146250824	q11.1 - q24.3	98182.963	Gain	0.4192093
	chr11	208365	51289318	p15.5 - p11.12	51080.954	Loss	-0.4354295
	chr11	55002923	64542307	q11 - q13.1	9539.385	Loss	-0.5233245
	chr12	9274400	34236852	p13.31 - p11.1	24962.453	Loss	-0.2772534
	chr12	102862386	132278059	q23.3 - q24.33	29415.674	Loss	-0.3105815
	chr13	18194544	114077122	q11 - q34	95882.579	Loss	-0.47626483

	chr16	94314	34634745	p13.3 - p11.1	34540.432	Loss	-0.42803276
	chr16	45174916	88563897	q11.2 - q24.3	43388.982	Loss	-0.39916563
	chr17	87009	17359944	p13.3 - p11.2	17272.936	Loss	-0.41903993
	chr17	38167926	78586290	q21.31 - q25.3	40418.365	Gain	0.34135374
	chr18	20264309	67431922	q11.2 - q22.3	47167.614	Loss	-0.46637988
	chr19	278273	18512927	p13.3 - p13.11	18234.655	Loss	-0.45161715
	chr19	18545314	24132581	p13.11 - p12	5587.268	Gain	0.5739976
	chr21	13562263	46433516	q11.2 - q22.3	32871.254	Loss	-0.4723002
076	chr1	143706582	246630459	q21.1 - q44	102923.878	Gain	0.5890351
	chr3	180303358	198161253	q26.32 - q29	17857.896	Loss	-0.29033908
	chr4	52383858	191004108	q11 - q35.2	138620.251	Loss	-0.27189562
	chr5	137805956	176748903	q31.2 - q35.3	38942.948	Gain	0.3074118
	chr6	88509378	170700061	q15 - q27	82190.684	Loss	-0.26758358
	chr7	673544	55936992	p22.3 - p11.2	55263.449	Loss	-0.32681513
	chr7	77892776	158018100	q21.11 - q36.3	80125.325	Gain	0.33464086
	chr8	617655	43291296	p23.3 - p11.1	42673.642	Loss	-0.3221669
	chr8	49101338	146250824	q11.21 - q24.3	97149.487	Gain	0.57531446
	chr9	490525	39146954	p24.3 - p13.1	38656.43	Loss	-0.25826296
	chr12	159909	34236852	p13.33 - p11.1	34076.944	Loss	-0.25632873
	chr13	18194544	114077122	q11 - q34	95882.579	Loss	-0.30596593
	chr14	19323579	105399038	q11.2 - q32.33	86075.46	Loss	-0.3187426
	chr16	45122058	88565680	q11.2 - q24.3	43443.623	Loss	-0.29477313
	chr17	87009	20589757	p13.3 - p11.2	20502.749	Loss	-0.32326612
	chr22	16023930	27707102	q11.1 - q12.1	11683.173	Loss	-0.3196139
077	chr1	144124745	244692977	q21.1 - q44	100568.233	Gain	0.39070973
	chr6	1051749	57183221	p25.3 - p12.1	56131.473	Gain	0.5915187
	chr7	7104046	55502877	p22.1 - p11.2	48398.832	Gain	0.36071604
	chr7	78474418	156281959	q21.11 - q36.3	77807.542	Gain	0.32592753
	chr8	84183236	145782038	q21.13 - q24.3	61598.803	Gain	0.61528206
	chr16	94314	34739434	p13.3 - p11.1	34645.121	Loss	-0.3962729
	chr16	45172598	88563897	q11.2 - q24.3	43391.3	Loss	-0.36009175
	chr17	87009	21857788	p13.3 - p11.2	21770.78	Loss	-0.38486278
	chr17	23115968	78623230	q11.1 - q25.3	55507.263	Gain	0.27112415
078	chr1	213927497	224318524	q41 - q42.12	10391.028	Gain	0.2953263
079	chr1	6334098	10518862	p36.31 - p36.22	4184.765	Loss	-0.3693026
	chr1	144188986	244692977	q21.1 - q44	100503.992	Gain	0.3242527
	chr6	25677110	29686713	p22.2 - p22.1	4009.604	Gain	0.29078814
	chr7	78692422	86480289	q21.11 - q21.12	7787.868	Gain	0.3150598
	chr8	617655	43291296	p23.3 - p11.1	42673.642	Loss	-0.32611796
	chr8	49101338	145836174	q11.21 - q24.3	96734.837	Gain	0.35293388
	chr18	38121554	76083117	q12.3 - q23	37961.564	Loss	-0.40700564
	chr21	13562263	46433516	q11.2 - q22.3	32871.254	Loss	-0.3894352
	chrX	77969673	154117857	q21.1 - q28	76148.185	Gain	0.7744805

Supplemental Table 2: List of Candidate and key genes in the recurrent CNAs for HCC*

**Refs**	Midorikawa et al., 2006	Schlaeger et al., 2008	Chochi et al., 2009	Jia et al., 2011	Guichard et al., 2012**	Wang et al., 2013	Gu et al., 2013	Qi et al., 2013	Liu et al., 2014
**Case No.**	36 resected HCC tumors	63 HCC samples	42 resected tumors	58 pairs of tumor/nontumor	125 tumor/nontumor	285 pairs of tumor/nontumor	Cancer Databae of HCC	32 HCC patients	16 HCC FFPE
**Methods**	SNP array	aCGH, quantitative RT-PCR	aCGH	SNP array, differentially	SNP array, whole exome sequencing	SNP array for CNAs and gene expression	OncoDB, HCC, HCC.net, COSMIC,	aCGH, iTRAQ, targeted mRNA and western blot	aCGH
**CNA gains**									
**1q**		BAT2D1, CFHR4, KIAA1096, **MDM4**	**AKT3**, ASTN, CD48, DIS204,DKFZPp434No250, ITPKB, LBR, M69199, M-ABC2, MAFG, NEK2, PBX1, PRCC, RYR2, SLC30A1, TGFB2, TAZ1	ARNT, LASS2, MCL1, MPZL1	**AKT3, ATF6**	ABL2, ARNT, BCL9	ADAM15, CKS1B, CREB3L4, LMCD1, mir190b, RAB1, S100A14, SHC1,	PDZK1, MCL1, ARNT, AF1Q, TPM3, ADAR, RPS27, HAX1, PYGO2, CKS1B, ADAM15, MUC1, HDGF, CCT3, PRCC, IFI16, AIM2, USF1, SELP, SELE, LAMC2, TPR, PTGS2, KIF14, ELF3, **MDM4**, **ATF3**, TGFB2, WNT3A, **AKT3**, EPHX1	PDE4DIP, BCL9, ARNT, TPM3, MUC1, PRCC, NTRK1, SDHC, FCGR2B, PBX1, ABL2, TPR, **MDM4,** ELK4, SLC45A3, H3F3A, FH
**2q**					**DNAJB3, NFE2L2**				AGAP1, LRP18
**5p**	ADCY2, MGC5297, MTRR	CDH6, CDH10		TERT			AMACR, DAB2, CDH12, LPCAT1, SEMA5A, TRIO	AMACR	IL7R, LIFR
**8q**		GLI4, MYC, PTK2, TATDN1, WISP1	D8S1108, EXT1, RECQ4, WI-13991	BOP1, DDEF1, HRY1, SCRIB		COX6C, MTDH		PRKDC, MCM4, SNAI2, LYN, MOS, PLAG1, COPS5, TPD52, E2F5, MMP16, NBS1,EIF3S3, C-MYC, KCNK9, PTK2, EIF2C2, CCNE2	CHCHD7, TCEA1, PLAG1, NCOA2, NBS1, HEY1, CBFA2T1, UBR5, COX6C, EXT1, MYC, NDRG1, RECQL4
**20p**									
**CNA losses**									
**1p**		COL11A1			**ARID1A, CDKN2C, CDK11A, CDK11B**	**ARID1A, CDKN2C**, TNFRSF14		AKR7A2, PRDM2, RIZ, RAD54L, FAF1, STIL, **CDKN2C**, TTC4, JUN, ARHI, PRDM2, RIZ, CASP9, PGM1, ENO1	TNFRSF14, PRDM16, RPL22, CAMTA1, SDHB, PAX7, MDS2
**2q**					**HAT1, MCM6**		LRP1B	AOX1, CYP27A1, HSPD1, HSPE1	BIN1
**4q**		CLDN22, FAT			**IRF2, SMARCAD1**			ADH4, ADH5, ADH6, ADH7, ADH1A, ADH1B, ADH1C, HADH, ACSL1, FGA, FGB, ACSL1 , CASP3, FAT, FSTL5, VEGFC	RAP1GDS1, TET2
**8p**		XKR6	EXTL3, WS-3	**DLC1**, TRIM35	**DLC1, BAG4**	WRN		EPHX2, CSMD1, DEFB1, NAT1, NAT2, PSD3, TNFRSF10A, TNFRSF10B,	PCM1
**10q**					**JMJD1C, MAPK8, PTEN, CYP2E1**	**PTEN**		**PTEN, CYP2E1**, ECHS1	CFL1P1, KLLN, **PTEN**
**13q**		P2RY5, SETDB2			**RB1**	BRCA2, **RB1**	TPTE2, Tg737	**RB1**, BRCA2, XPO4,CCNA1, RFP2, DDX26, DLEU1, DLEU2	
**16q**		NQO1						ARG1, CDH1, CDH3, CDH13,BCAR1, WWOX, WFDC1	
**17p**		ALDH3A1, MFAP4	ABR, ALOX12, GAS7, SCO1	ENO3, PER1, **TP53**	**CHD3, TP53**			**TP53** , MYH10	USP6, TP53, PER1, GAS7, MAP2K5

*Candiate genes in other CNAs from the literature were not included

Key genes and pathways for HCC by Guichard et al., 2012 [** 21 key genes (**in bold**) located in the recurrent CNAs out of the 56 genes]

beta-catenin		p53/cell cycle control		chromatin remodeling		PI3K/Ras signaling		Oxidative and endoplasmic reticulum stress	
FZR1	19p13.3	**IRF2**	**4q35**	SMARCA2	9p24.3	KRAS	12p12	EIF2AK3	2p11.2
CSNK1E	22q13	MDM2	12q15	SMARCA4	19p13.2	PIK3CA	3q26	ERN1	17q23.3
CTNNB1	3p22	**TP53**	**17p13.1**	SMARCB1	22q11.23	**MAPK8**	**10q11.22**	**ATF6**	**1q23.3**
AXIN1	16p13.3	**RB1**	**13q14.2**	**ARID1A**	**1p36**	MAP3K12	12q13.13	**NFE2L2**	**2q31**
APC	5q22.2	CDKN2A	9p21.3	ARID2	12q12	PIK3CG	7q22.3	KEAP1	19p13.2
MCC	5q22.2	**CDKN2C**	**1p32.3**	PBRM1	3p21.1	**PTEN**	**10q23.31**	EDEM1	3p26.1
		CDKN1B	12p13.1	SMARCA1	Xq25	PRKCB	16p12.2	PDIA2	16p13.3
		**CDK11A**	**1p36.33**	**SMARCAD1**	**4q22.3**	RPS6KA3	Xp22.11	PARK7	1p36.23
		**CDK11B**	**1p36.33**	ARID4A	14q23.1	STK11	19p13.3	CCT6B	17q12
		PAK2	3q29	JMJD8	16p13.3	**DLC1**	**8p22**	**DNAJB3**	**2q37.1**
				**JMJD1C**	**10q21.3**	STRADA	17q23.3	DNAJC22	12q13.12
				EP300	22q13.2	AKT1	14q32.33	CCT8L2	22q11.1
				**CHD3**	**17p13.1**	AKT2	19q13.2	BAG3	10q26.11
				CHD4	12p13.31	**AKT3**	**1q43**	**BAG4**	**8p11.23**
				**HAT1**	**2q31.1**			NOX5	15q23
				HADC9	7p21.1			NOS3	7q36.1
				HIST1H2BF	6p22.2			SOD1	21q22.11
				**MCM6**	**2q21.3**			**CYP2E1**	**10q26.3**
								CYP2F1	19q13.2
